# A ferroelectric-ionic-trapping transistor for low power and secure neuromorphic computing

**DOI:** 10.1038/s41467-026-72971-y

**Published:** 2026-05-29

**Authors:** Changhyeon Han, Youngchan Cho, Dongbin Kim, Se-Hyun Hwang, Minsuk Song, Been Kwak, David Radermacher, Min Wook Kang, Sangwan Kim, Jangsaeng Kim, Wonjun Shin, Daewoong Kwon

**Affiliations:** 1 Department of Electrical Engineering, Hanyang University, Seoul, Republic of Korea; 2 Department of Semiconductor Convergence Engineering, Sungkyunkwan University, Suwon, Republic of Korea; 3 Department of Semiconductor Engineering, Sogang University, Seoul, Republic of Korea; 4 Department of Electrical and Computer Engineering, Sungkyunkwan University, Suwon, Republic of Korea; 5 Department of Electronic Engineering, Sogang University, Seoul, Republic of Korea; 6 Department of System Semiconductor Engineering, Sogang University, Seoul, Republic of Korea

**Keywords:** Electrical and electronic engineering, Electronic devices

## Abstract

The convergence of artificial intelligence and pervasive data analytics has created an urgent demand for energy-efficient and secure computing hardware. Neuromorphic synaptic devices emulate the human brain with high parallelism and 3D connectivity to reduce power consumption, yet they lack intrinsic mechanisms to protect stored information from malicious read-out. Here we report a ferroelectric–ionic–trapping field-effect transistor (FITFET) that integrates ferroelectric polarization, oxygen-vacancy migration, and charge trapping to enable both synaptic and secure functionality. The FITFET operates in two programmable regimes: a plain mode combining fast, low-power ferroelectric switching with analog weight modulation, and a secure mode in which the memory window collapses under specific read biases, concealing stored states and suppressing read attacks. This reversible, voltage-controlled transition provides a hardware-native data protection mechanism. Multiscale analyses demonstrate reliable switching between learning and concealment, while system-level simulations show reduced model inversion attacks with minimal accuracy loss.

## Abstract (web summary)

Computing efficiency and data security are two critical demands in the AI era. Han et al. report a ferroelectric transistor with controllable synaptic and secure functionalities. It physically hides stored data to block read attacks. Simulations show its array effectively reduces model inversion attacks.

## Introduction

**Fig. 1:**
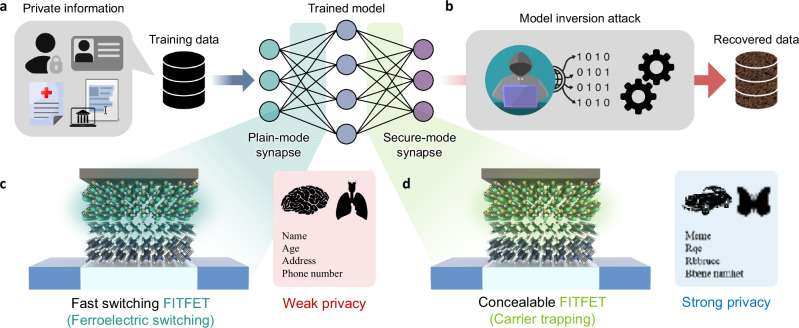
Hardware protection against privacy leakage in neuromorphic computing. **a** Training phase encoding sensitive private information (medical and financial data) into the neural network. **b** Illustration of a model inversion attack recovering original training data. **c** Plain mode operation based on fast ferroelectric switching. Plain mode supports efficient computation but leaves the system susceptible to inversion, resulting in feasible reconstruction and revealed input information. **d** Secure mode operation utilizing carrier trapping, rendering the system resistant to inversion with suppressed reconstruction and concealed input information. The crystal structure of FET was visualized using VESTA^60^.

Rapid advances in artificial intelligence are driving an unprecedented growth in computational demand, raising serious concerns over the energy footprint of modern information technology. As workloads scale from edge devices to hyperscale data centers, simply improving the efficiency of computation based on conventional von Neumann architecture is no longer sufficient^1–4^. Future hardware should not only deliver energy-efficient computing but also integrate multiple advanced functions—notably on-chip security—to protect the increasingly sensitive data processed by artificial intelligence (AI) systems against adversarial threats: model inversion (MI) attacks, where private information can be reconstructed from trained synaptic weights (Fig. 1a–b). This dual requirement motivates the development of device platforms that can concurrently support neuromorphic computing beyond von Neumann and hardware-level secure computing, rather than treating these as separate, disjoint technology tracks.

However, in state-of-the-art systems these capabilities are typically implemented by distributing them across distinct semiconductor materials and device types, because the physical requirements for high-fidelity neuromorphic computation and robust hardware-level security are markedly different. For example, neuromorphic accelerators commonly employ devices that are specifically optimized for low-power analog weight storage and update—such as ferroelectric^5,6^, phase-change^7,8^, or oxide-based synaptic transistors^9,10^—whereas hardware security primitives are instead realized using separate structures, including variability-exploiting memristor-based physical unclonable functions (PUFs)^11,12^ random telegraph noise^13,14^ and polymorphic or camouflaged logic elements^15,16^ for obfuscation and authentication. As a result, most emerging devices are tailored either for energy-efficient analog computing or for dedicated security functions, but rarely integrate all of these functionalities within a single platform. Instead, a functionally diverse material system is required, in which multiple physical mechanisms can be co-engineered and selectively addressed within one device architecture, so that these heterogeneous neuromorphic and security functions can be monolithically integrated.

In this context, HfO_2_ stands out as a versatile material platform to meet these demands. When appropriately doped or compositionally engineered, as demonstrated in HfO_2_–ZrO_2_ (HZO), these films exhibit robust ferroelectricity in thin layers, enabling low-voltage polarization switching^17,18^. At the same time, ionic species such as oxygen vacancies (V_O_s) inherent to HfO_2_ underpin resistive switching behavior^19,20^. Although ferroelectric switching and vacancy-mediated resistive switching originate from different mechanisms and their coexistence has often been regarded as detrimental due to mutual performance degradation^21^, rational materials and stack engineering can instead turn this incompatibility into a benefit. Moreover, hafnia-based gate stacks in field-effect transistors (FETs) invariably incorporate an adjacent dielectric interlayer, such as SiO_2_, forming a ferro–dielectric interface that hosts a rich landscape of trap states^22,23^. When the thickness, composition, and band alignment of this interlayer are systematically engineered, controlled charge trapping dynamics at the ferro–dielectric interface can electrostatically screen or modulate the ferroelectric polarization, introducing a third, trap-mediated switching degree of freedom. By deliberately controlling this coupled ferro-ionic–trapping complex, one can not only enhance the intrinsic performance of each individual switching mode, but also harness their distinct electrical signatures as orthogonal degrees of freedom to implement multiple device functions within a single material stack. Crucially, this multifunctional ferro-ionic–trapping landscape is available on a platform that is already deeply embedded in industrial processes: hafnia constitutes the gate stack in advanced high-k/metal-gate logic^24–26^, while zirconia is adopted in DRAM capacitors^27^, collectively demonstrating full complementary metal-oxide-semiconductor (CMOS) and very-large-scale integration (VLSI) scalability.

Building on this opportunity, we propose a ferroelectric–ionic–trapping field-effect transistor (FITFET) that leverages the synergistically coupled ferro-ionic–trapping interaction as a unified device platform for both neuromorphic computing and hardware-native security (Fig. 1c–d). By engineering a thin SiO_2_–TiO_2_ interfacial stack on HZO, the FITFET operates in three bias-addressable regimes: ferroelectric polarization switching at low program bias, trap-mediated memory-window (MW) collapse by the ferro-dielectric interface, and oxygen-vacancy (V_O_)–driven ionic switching, thereby functioning as a bias-programmable synapse with a built-in concealable readout mode. We demonstrate that in the plain mode (Fig. 1c), the device supports low-voltage, high-fidelity analog weight modulation, while in the secure mode (Fig. 1d), the MW collapses under specific read biases, physically hiding the stored conductance states without destructive erasure. Through combined structural, spectroscopic, and low-frequency noise (LFN) spectroscopy, we elucidate the microscopic origin of evolution in switching behaviors. Furthermore, it is confirmed that the same engineered stack reliably scales to a wafer-level 24 × 12 FITFET array that demonstrates voltage-controlled image conceal–reveal operations. Finally, system-level simulations using experimentally calibrated device characteristics establish that FITFET-based synaptic arrays can substantially mitigate MI attacks on both benchmark and medically relevant datasets, with only marginal impact on inference accuracy. These results position the FITFET as a CMOS-compatible, multifunctional synaptic transistor that unifies energy-efficient neuromorphic computing and hardware-level data protection within a single, industrially relevant material platform.

## Results and discussion

### A bias-programmable synapse with concealable readout

The synapse is the central hardware primitive of neuromorphic computing, storing and continuously modulating analog weights that govern signal transmission between neurons. In this study, we implement the synapse with a FITFET that operates through three bias-addressable mechanisms: ferroelectric switching in HZO for fast, low-voltage weight updates; interface-trap–induced MW collapse, whereby trap charging screens the ferroelectric polarization and transiently suppresses MW; and V_O_–based ionic switching that broadens the analog tuning range. A thin SiO_2_ interface layer (IL) is pivotal to both switching efficiency and concealable readout. In the plain mode synapses, operated under a low read bias regime corresponding to the ferroelectric operating region, the minimal thickness of the IL ensures that the applied gate bias couples almost entirely into the HZO layer, yielding low-voltage synaptic weight updates (Fig. 1c). Furthermore, when the read bias is elevated, the device transitions into the secure mode (ii), in which charging traps at the IL to collapse the MW and thereby conceal the stored weight during readout without perturbing the non-volatile state (Fig. 1d). This bias-only toggle enables erase-free, damage-free switching between accurate inference and protected readout within a single device, thereby circumventing the endurance penalties inherent to erase–reprogram–based concealment.

### Device structure of transistor

The devices were fabricated on 150 mm silicon-on-insulator (SOI) wafer using a gate-first process with fully CMOS-compatible materials and processes, ensuring extendibility to 300 mm wafers. The fabrication process is illustrated and explained in Supplementary Fig. 1 and Methods. The gate stack consists of HfO_2_–ZrO_2_ (HZO, 6.4 nm) as the ferroelectric layer, as detailed in Supplementary Note 1, and a thin SiO_2_–TiO_2_ interfacial layer (1.33 nm in total), in which the oxygen-scavenging TiO_2_ sublayer promotes controlled V_O_ migration while the thin SiO_2_ layer promotes trapping dynamics. Here, it is important to note that, in order to systematically identify the role of the engineered interfacial stack, we additionally fabricated two control devices with the same HZO thickness: a conventional FeFET with a SiO_2_ (1.73 nm) –HZO (6.4 nm) gate stack and a TiO_2_-interfaced ferro-ionic duality (FIDFET) with a SiO_2_–TiO_2_ (1.92 nm)–HZO (6.4 nm) stack. Systematic structural and electrical comparisons among these devices confirm that the non-conventional characteristics of the proposed FITFET arise from deliberate interface engineering rather than unintended process-induced artifacts.

**Fig. 2:**
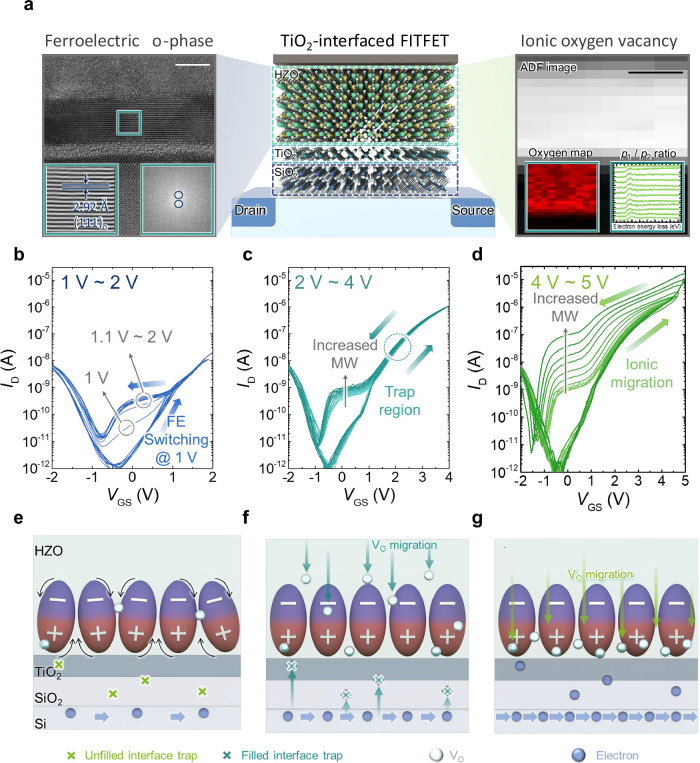
Structural and switching mechanisms of the TiO_2_ interfaced FITFET. **a** Atomic-scale structure images showing the orthorhombic ferroelectric phase in HZO (left, scale bar 5 nm), the TiO_2_-interfaced gate stack of the FITFET (center), and the V_O_ related map obtained by EELS (right, scale bar 5 nm), **b** *I*_D_-*V*_GS_ transfer characteristics measured at different *V*_GS_ regimes: **c** ferroelectric polarization switching at 1–2 V, **d** trap-dominated screening and MW collapse at 2–4 V, and **e** ionic migration assisted MW expansion at 4 – 5 V. Schematic illustrations of the corresponding mechanisms: **e** ferroelectric dipole switching, **f** interface trap filling and charge screening, and **g** field-driven V_O_ migration.

The elemental compositions of the gate stacks for each transistor were confirmed through energy-dispersive X-ray spectroscopy (Supplementary Fig. 2 and Methods). To elucidate the structural origin of ferroelectricity in the HZO layer, atomic-scale imaging was performed using transmission electron microscopy (TEM), which revealed well-defined lattice fringes consistent with the ferroelectric orthorhombic phase (Fig. 2a and Supplementary Fig. 3)^28^. To further confirm the phase formation, grazing-incidence X-ray diffraction (GIXRD) measurements were conducted, showing distinct diffraction peaks corresponding to the orthorhombic phase of HZO (Supplementary Fig. 4)^29^, together with reference data from the FeFETs and FIDFETs confirming consistent crystallinity across different interface configurations. These results verify that the HZO layer in the FITFET maintains stable orthorhombic ferroelectric crystallinity under the proposed interface engineering.

In addition, the introduction of a TiO_2_ interlayer induces partial oxygen scavenging at the HZO interface, leading to a gradient in the concentration of V_O_s within the HZO layer. The spatial distribution and chemical states of V_O_s within the HZO layers in each transistor were characterized by electron energy-loss spectroscopy (EELS) and X-ray photoelectron spectroscopy (XPS) (Supplementary Notes 2 and 3)^30–32^. EELS analysis revealed a clear reduction in the *p*_1_/*p*_2_ intensity ratio near the HZO/IL interface, indicating a higher relative concentration of V_O_s in the transistors with TiO_2_ layer compared to the FeFET without TiO_2_ (Fig. 2a, Supplementary Figs. 5 and 6). Complementary XPS depth-profiling further confirmed this behavior, showing an increased oxide-deficient fraction in the TiO_2_-interfaced gate stacks relative to the FeFET without TiO_2_ (Supplementary Fig. 7). These observations demonstrate that the TiO_2_ layer functions as an oxygen-scavenging interlayer, facilitating directional oxygen migration and modifying the interfacial switching dynamics. The proposed stack enables a balanced coexistence of ferroelectric dipoles and mobile ionic species, enabling hybrid ferro-ionic switching. This coupling allows simultaneous control of dipole orientation and V_O_ movement, broadening the MW beyond purely ferroelectric operation.

Expanding upon this concept, the proposed FITFET further refines the interfacial state through further thinning of the SiO_2_ interlayer. In conventional FeFETs, the SiO_2_ layer acts as a passive interface that induces ferroelectric dipole shielding due to interface traps (*Q*_it_), thereby reducing the MW. In contrast, the proposed FITFET integrates a TiO_2_ layer together with SiO_2_ interlayer thinning, transforming the interface from a passive barrier into an active functional element. This modification facilitates low-voltage ferroelectric switching and, via controlled MW collapse under appropriate biases, enables physical concealment of the stored synaptic weights.

### Bias dependent switching dynamics

Figure 2b–d shows the transfer characteristics (*I*_D_–*V*_GS_s) of the proposed FITFET measured under various *V*_GS_ values, and the corresponding switching behaviors are illustrated in Fig. 2e-g. Unlike conventional FeFETs that exhibit a monotonic widening of the MW with increasing bias, the FITFET shows distinct bias-dependent transitions of its *I*_D_–*V*_GS_ and MW behavior. The device operation can be divided into three distinct regions according to the applied *V*_GS_.

The operation can be divided into three regions. Region I correspond to ferroelectric switching. At low *V*_GS_ ( ≈ 1–2 V, Fig. 2b, e), the FITFET exhibits stable ferroelectric hysteresis governed by polarization switching within the HZO layer. In this regime, the ferroelectric polarization effectively modulates the channel potential through the thin SiO_2_–TiO_2_ interfacial stack, establishing an asymmetric electrostatic potential distribution across the gate stack and producing distinct On/Off conduction states. Owing to the thin SiO_2_–TiO_2_ (~ 1.33 nm) interfacial layers, the ferroelectric switching occurs at a very low *V*_GS_ ( ≈ 1.0 V), which is significantly lower than the FeFET that begins to exhibit a MW only above 3.5 V (Supplementary Fig. 8). This demonstrates that interlayer thinning substantially enhances the field efficiency for ferroelectric polarization switching, reducing the required switching voltage.

Region II corresponds to MW collapse. When the *V*_GS_ is increased to an intermediate range (≈ 2–4 V, Fig. 2c, f), the forward and reverse sweep curves overlap, indicating the collapse of the MW. This behavior originates from the electron trapping at SiO_2_–TiO_2_ interfacial traps. Electrons injected from the channel are captured by these sites, forming a negatively charged layer that electrostatically screens the ferroelectric polarization. Consequently, the ferroelectric polarization–channel coupling is substantially weakened, leading to a temporary decoupled state where the ferroelectric polarization remains active, but its modulation of the channel potential is effectively inhibited. In conventional FeFETs, polarization compensation by the trapped charges and decrease or collapse of MW are considered negative effects of the interface dielectric^33,34^. However, in the proposed FITFET, it is important to note that this MW collapse at low *V*_GS_ sweep is intentionally exploited to realize the concealable synaptic behavior of the FITFET.

Region III corresponds to ionic switching. When a higher *V*_GS_ ( ≈ 4–5 V, Fig. 2d, g) is applied, the MW reopens and expands beyond the initial ferroelectric-driven range. Under this condition, migration of positively charged V_O_s (V_O_^1+^ or V_O_^2+^) becomes the dominant switching mechanism of the HZO layer^35,36^. As these V_O_s drift toward the channel, V_O_s introduce an additional positive electrostatic field that further modulates the channel potential^37^. This field-driven ionic distribution results in a progressive increase in both the MW and the on-state current (*I*_ON_).

These results collectively demonstrate that the FITFET undergoes an *open–collapse–reopen* transition of the MW, corresponding to three bias-dependent regimes of ferroelectric polarization, trap-mediated ferroelectric screening, and ionic V_O_ migration. Supplementary Fig. 9 further corroborates these bias-dependent transitions by illustrating the corresponding gate current (*I*_G_) evolution across the three regimes. Distinct polarization switching peaks are observed at low *V*_GS_ ( ~ 1.0 V), while a rapid increase in *I*_G_ appears at higher *V*_GS_ due to field-driven ionic migration. These behaviors are elucidated through comparison with previously reported FeFET and FIDFET structures (Supplementary Note 4, Supplementary Fig. 10), providing a broader insight into how interface engineering—particularly the incorporation of the thin SiO_2_–TiO_2_ stack—modulates the interplay among ferroelectric, trap-mediated, and ionic processes.

### In situ low frequency noise analysis

We conducted shows LFN spectroscopy across the three operating regimes, elucidating the underlying switching dynamics. LFN serves as a sensitive, nondestructive probe of defect dynamics and trap–channel coupling in nanoscale transistors^38,39^. In particular, LFN is highly advantageous for elucidating switching mechanisms with in-situ and non-destructive properties. Unlike in-situ TEM, which requires complex sample preparation and is challenging to apply to nanoscale devices, LFN measurements can directly probe the underlying physical processes under actual bias conditions, providing practical insight into device operation in real time^40–42^.

**Fig. 3:**
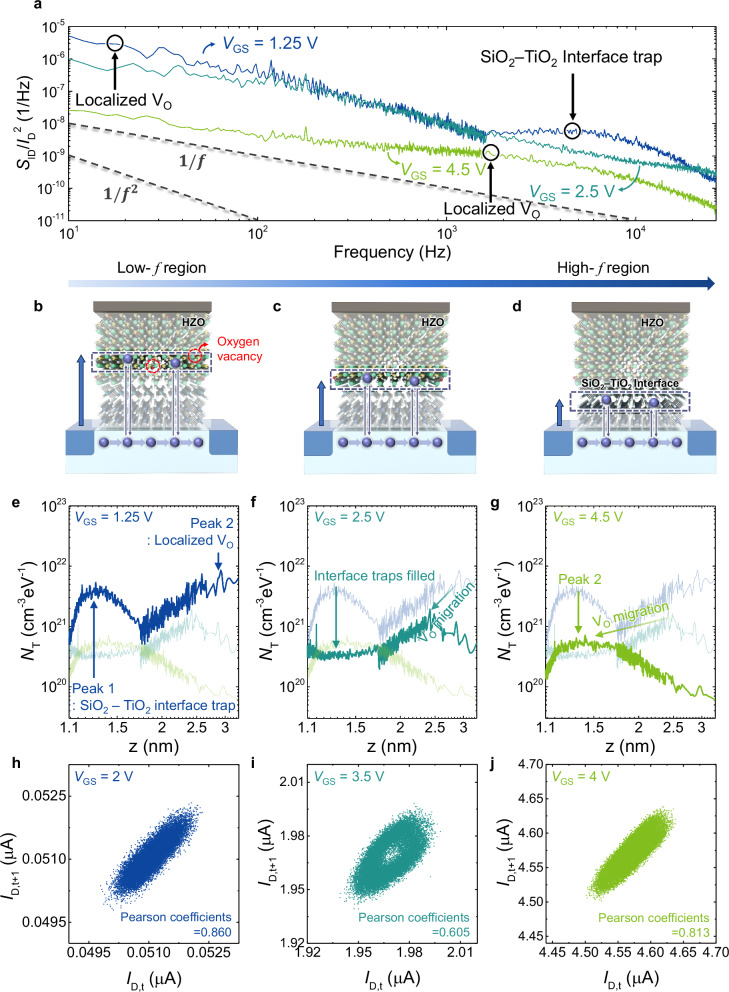
LFN and autocorrelation analyses across the operating regimes in the FITFET. **a** *I*_D_ normalized power spectral density (PSD) (*S*_ID_/*I*_D_^2^) of the FITFET under different *V*_GS_ values **b**–**d** Schematic illustrations of position-dependent trapping/detrapping dynamics across the gate stack as a function of frequency, from V_O_ migration (low-frequency regions) to SiO_2_-TiO_2_ interface (high-frequency regions). **e**–**g** Depth-resolved *N*_T_ profiles extracted by LFN measurements under different *V*_GS_ values. **h**–**j** Scatter plots of *I*_D,t_ versus *I*_D,t+1_ for the different *V*_GS_ conditions, showing the corresponding Pearson correlation coefficients.

Figure 3a shows *I*_D_-normalized power spectral density (PSD) (*S*_ID_/*I*_D_^2^) at three representative *V*_GS_ values for the ferroelectric switching (*V*_GS_ = 1.25 V), trap-mediated MW collapse (*V*_GS_ = 2.5 V), and ionic switching (*V*_GS_ = 4.5 V) regimes. At *V*_GS_ = 1.25 V, the spectrum shows a comparatively steep low-frequency slope and a distinct hump at a frequency of 5 kHz. The hump in the PSD indicates that there are localized traps that govern the conduction of the transistor, which will be elaborated in more detail later. Note that this bias-dependent LFN behavior differs from conventional FeFETs, which typically exhibit a gate-bias-insensitive 1/*f* ^γ^ spectrum with *γ* = 1, consistent with standard interface-trap-dominated fluctuations in a SiO_2_–HZO stack (Supplementary Fig. 11). At *V*_GS_ = 2.5 V, the PSD exhibits an approximately constant slope over the measured band with no discernible hump. At *V*_GS_ = 4.5 V, a single corner frequency is observed near 1.8 kHz, beyond which the slope increases noticeably toward higher frequencies. These spectral characteristics can be analyzed within the carrier-number-fluctuation (CNF) framework, which enables the transformation of frequency-dependent spectra into spatially resolved depth profiles (Supplementary Note 5 and Supplementary Fig. 12)^43,44^. Through this approach, the bias-dependent evolution of defect dynamics within the gate stack can be examined, providing insight into how the switching mechanism modulates the interfacial and bulk defect distributions.

Figure 3b–d shows the depth-resolved defect mapping of the gate stack, where the noise spectra are decomposed into three frequency regions in LFN measurements corresponding to different trap locations across the SiO_2_–TiO_2_–HZO stack. The low-frequency regime (~ 100 Hz) corresponds to traps with long time constants located at the bulk region of the HZO layer. The mid-frequency regime (100 ~ 4 kHz) probes traps with intermediate time constants, primarily distributed toward the bottom region of the HZO layer adjacent to the TiO_2_ layer. The high-frequency regime (4 kHz~ 26.6 kHz) is associated with fast traps residing near the SiO_2_–TiO_2_ interfacial region, where capture and emission of carriers occur rapidly under strong local fields. Further methodological details on the frequency-to-depth conversion procedure are provided in Supplementary Note 5.

Figure 3e–g shows the corresponding depth profiles of trap density (*N*_T_) extracted at three representative *V*_GS_. At *V*_GS_ = 1.25 V (Fig. 3e), two distinct peaks are observed: Peak 1 appears at ≈ 1.3 nm, attributed to traps located near the SiO_2_–TiO_2_ interface, while Peak 2, emerging beyond 3 nm, corresponds to localized V_O_ states within the HZO layer. When the *V*_GS_ increases to 2.5 V (Fig. 3f), Peak 1 disappears and Peak 2 shifts toward a shallower depth, implying that electrons in the channel fully fill the traps at the SiO_2_–TiO_2_ interface and V_O_s originating from the HZO layer migrate toward the bottom region of the HZO layer. At higher bias (*V*_GS_ = 4.5 V) (Fig. 3g), V_O_s further migrate to the TiO_2_–HZO interface, aggregating into a new cluster of defects and demonstrating a new trap distribution that reflects the field-driven ionic movement across the stack. This sequential evolution of the *N*_T_ profile confirms the field-assisted electron filling at the SiO_2_–TiO_2_ interface and the subsequent redistribution of V_O_s across the stack, highlighting the coupling between electronic trapping and ionic migration that governs the ferroelectric-ionic-trapping dynamics in the FITFET structure.

Figure 3h–j shows the evolution of the Pearson correlation coefficient (*ρ*) extracted from the autocorrelation between consecutive current traces (*I*_D,t_ and *I*_D,t+1_) under different gate biases (Supplementary Figs. 13 and 14). Autocorrelation analysis serves as a time-domain indicator of how the conduction-governing defects are spatially localized. A higher *ρ* corresponds to a larger population of clustered defects that induce temporally correlated carrier fluctuations, whereas a lower *ρ* indicates more uniformly distributed traps leading to uncorrelated noise behavior^45^. The evolution of *ρ* with *V*_GS_ was examined to trace how defect localization changes across different switching regimes. At 2 V (Fig. 3h), SiO_2_–TiO_2_ interface traps and localized V_O_ in the top HZO region form clustered centers, giving a high *ρ* = 0.860. At 3.5 V (Fig. 3i), interfacial trap filling and partial V_O_ delocalization reduce correlation (*ρ* = 0.605). At 4 V (Fig. 3j), V_O_ re-aggregate near the TiO_2_–HZO interface, raising *ρ* to 0.813. This trend remains consistent across different lag times, indicating that the correlation behavior is robust and not sensitive to the specific temporal window used for analysis (Supplementary Fig. 15).

### Secure and plain operation

**Fig. 4:**
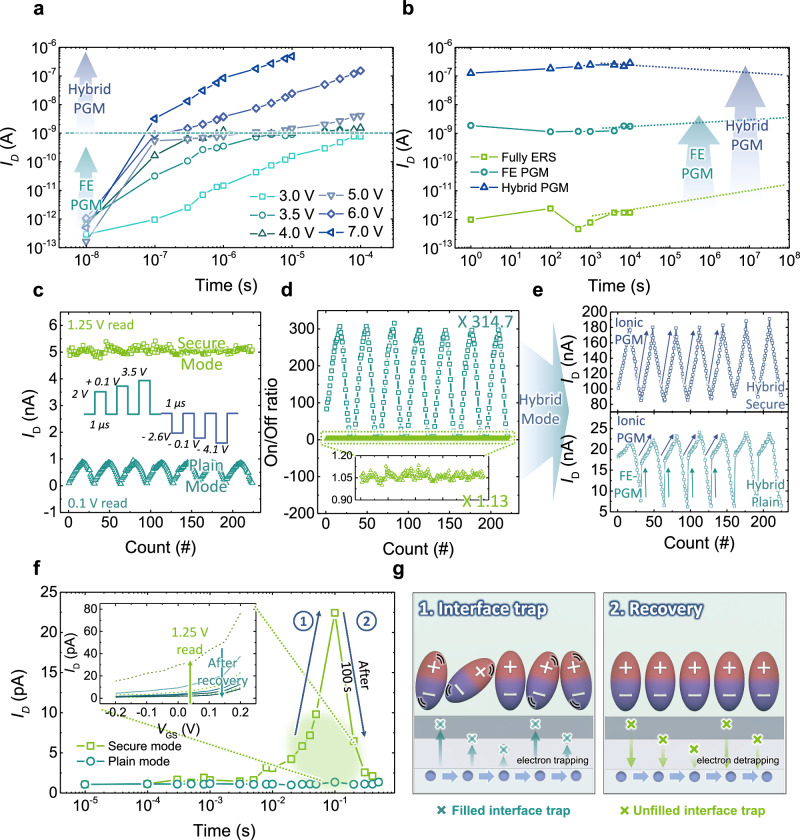
Bias programmable plain and secure mode operation of the FITFET. **a** Transient PGM characteristics under various *V*_PGM_s, showing the transition from ferroelectric PGM at low *V*_PGM_s to hybrid ferro-ionic PGM at higher *V*_PGM_s. **b** Retention characteristics of fully ERS, FE-PGM, and hybrid-PGM states, confirming nonvolatile stability across operating regimes. **c** Incremental conductance update measured under low *V*_read_ (0.1 V, plain mode) and high *V*_read_ (1.25 V, secure mode), demonstrating MW collapse and weight update suppression during secure mode readout. **d** Evolution of on/off ratio in plain and secure modes. **e** High-voltage incremental pulse response (5.5 – 7 V), showing enhanced linearity in hybrid PGM and the emergence of ionic contributions at higher *V*_PGM_s. **f** Read-state stability test under plain and secure modes. Secure mode read induces a temporary polarization disturbance followed by full recovery after removing the read bias. **g** Schematic interface traps and recovery processes during secure mode.

To evaluate the synaptic functionality, we examined the temporal and dynamic behavior of the FITFET. The switching speed and retention characteristics, shown in Figs. 4a, 4b and Supplementary Fig. 16, confirm the reliability and dynamic duality of the ferro-ionic operation. Figure 4a shows that polarization-driven switching occurs at low *V*_GS_ with a fast timescale (≈ 100 ns), while the ionic contribution emerges at higher *V*_GS_ with a slower response, verifying the coexistence of fast ferroelectric and slow ionic dynamics within the FITFET (Supplementary Fig. 17). Meanwhile, Fig. 4b shows both ferroelectric (FE PGM) and hybrid (Hybrid PGM) states exhibit excellent nonvolatile retention without noticeable degradation, validating the long-term reliability of the ferro-ionic operation. Both polarization- and ionic-driven switching behaviors exhibit high uniformity and reproducibility across the FITFET wafer, as confirmed by the statistical distributions in Supplementary Fig. 18. Specifically, the MW collapse associated with the trap-mediated secure mode occurs reproducibly at *V*_GS_ ≈ 1.18–1.21 V across devices, while the onset of ionic contribution is observed beyond ~3.9 V, clearly defining the bias boundaries between the ferroelectric, trapping, and hybrid regimes. The robustness of the bias-controlled mode transition was further confirmed through repeated mode-toggling measurements and long-term endurance measurements in Supplementary Figs. 19 and 20.

Figure 4c–e demonstrate how the FITFET exploits this coupling to realize reversible, bias-controlled data concealment. Incremental pulse measurements were conducted using positive pulses ranging from 2 V to 3.5 V for potentiation and negative pulses from −2.6 V to −4.1 V for depression, each applied with a pulse width of 1 µs under varying read voltages (*V*_read_) (plain and secure modes). Under a low *V*_read_ (0.1 V), the device operates in a plain mode characterized by stable polarization switching and consistent on/off states across repeated incremental pulses. The read-out current changes linearly with the number of applied pulses, indicating uniform polarization accumulation and excellent analog linearity during potentiation and depression. In contrast, when the *V*_read_ is raised to 1.25 V, the device enters the secure mode, in which the on/off distinction disappears, and the read-out current response becomes highly distorted (Fig. 4c). Figure 4d compares the corresponding on/off ratios in the plain and secure modes, clearly showing the abrupt suppression of the memory margin under secure mode.

At higher pulse voltages (Fig. 4e), incremental pulse measurements were performed using 5.5 V to 7 V for potentiation and −3.5 V to −5 V for depression to probe the hybrid ferro-ionic switching operation beyond the purely ferroelectric switching observed in Fig. 4c. It should be noted that this hybrid ferro-ionic regime is not intended to enable information masking, but rather to explicitly probe the contribution of ionic species under high-bias conditions and to demonstrate an additional synaptic modulation— potentially useful for applications requiring a wider dynamic range—that is accessible only in this elevated-bias regime. Under these conditions, the FITFET exhibits linear and symmetric current responses even within the secure mode region, which is attributed to excessive field-driven V_O_ migration across the HZO. In the plain mode region, the first pulse induces a steep current increase due to ferroelectric polarization switching, followed by a gradual and nearly linear evolution, indicating a transition toward the ferro-ionic hybrid state. These results confirm that when the applied bias is low, the FITFET can reliably store and conceal synaptic weights, whereas under sufficiently high bias, V_O_ migration becomes activated—serving as a supplementary high-bias operation mode rather than a concealment mechanism and providing direct physical evidence of the underlying ionic contribution to the encryption mechanism.

To verify the data integrity during operation, transient current measurements were performed under plain (0.1 V) and secure (1.25 V) reading conditions (Fig. 4f). At plain mode, the transient current remains stable over time without any *I*_D_ drift. In contrast, under the secure mode, a slight increase in current appears when a 100 ms read pulse is applied, followed by a gradual recovery once the bias is removed. This transient response does not originate from ferroelectric switching but rather from a temporary perturbation of dipole alignment near the SiO_2_–TiO_2_ interface, where the polarization momentarily fluctuates under the elevated *V*_read_. After bias removal, the dipoles relax back to their original upward-oriented configuration, confirming that the secure mode operation induces a reversible polarization disturbance rather than permanent data loss, as schematically illustrated in Fig. 4g. Following this reversible polarization disturbance, the FITFET can fully restore its original conductance state, ensuring error-free retrieval during the operation. This behavior confirms that the concealable readout is governed not by destructive charge-loss mechanisms but by reversible dipole-trap interaction intrinsic to the engineered SiO_2_–TiO_2_ interface. This stability is further supported by the negligible current evolution under repeated secure-mode pulse stress (Supplementary Fig. 21). Importantly, this bias-programmable transition does not induce degradation in either the ferroelectric or ionic components, enabling repeated toggling between plain and secure modes without endurance penalties.

### Hardware demonstration of concealable readout

**Fig. 5:**
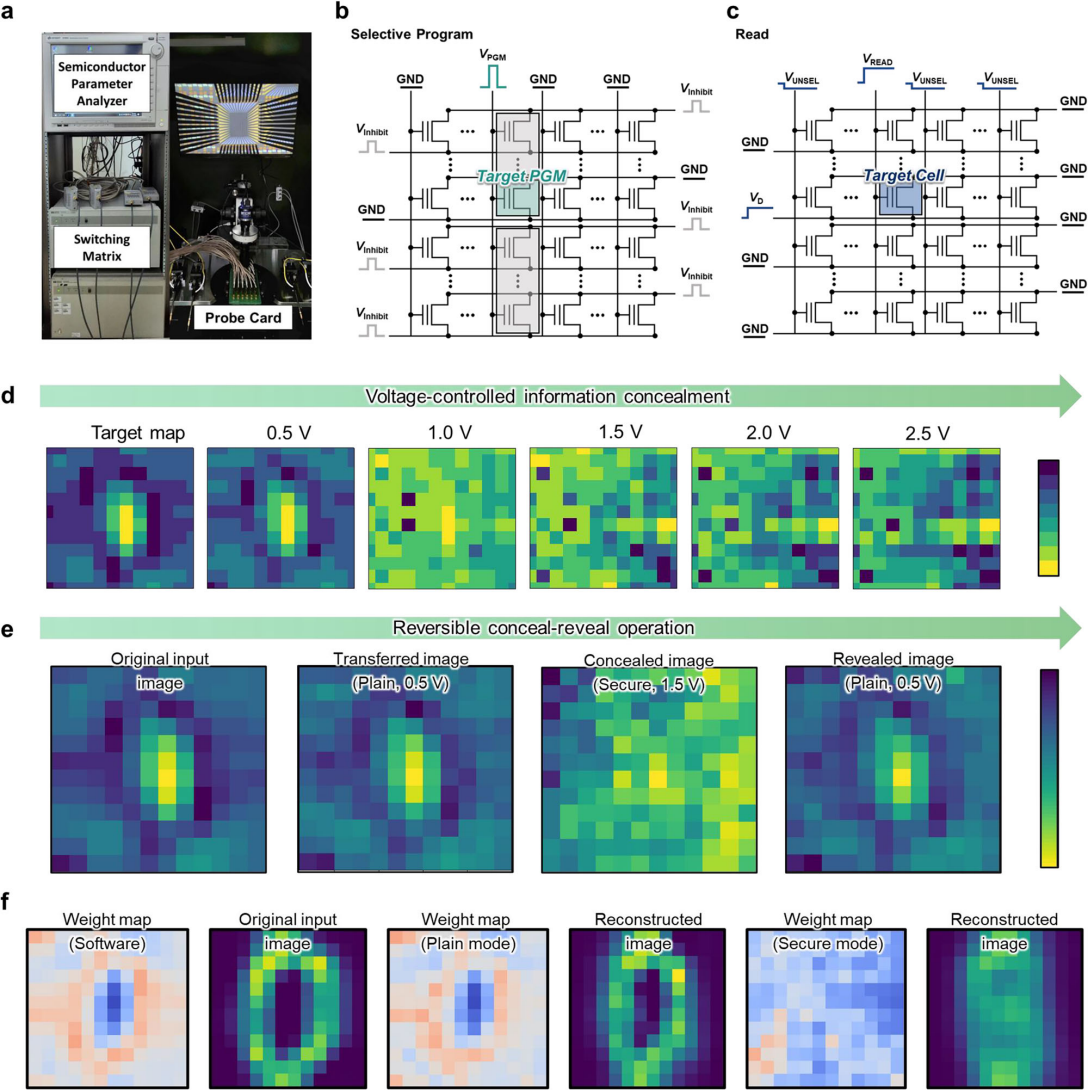
Array level verification of voltage-controlled conceal reveal operation in arrays. **a** Measurement setup using a semiconductor parameter analyzer, switching matrix, and probe card. Bias schemes for **b** selective program and **c** read operations. **d** Voltage-controlled data concealment: a 12 × 12 input image transferred at *V*_read_ = 0.5 V becomes gradually concealed as the *V*_read_ increases. The color scale represents conductance ranging from 0.106 to 0.652 µS. **e** Reversible conceal-reveal operation showing image hiding at secure mode (1.5 V) and recovery at plain mode (0.5 V). The color scale represents conductance ranging from 0.45 to 2.3 nS. **f** Comparison of reconstructed images and corresponding weight maps between plain and secure modes.

The above-mentioned device characteristics establish the FITFET as a synaptic element capable of simultaneously supporting reliable analog learning and hardware-level data protection. We further explored whether such secure neuromorphic computation can be extended to large-scale systems, demonstrating array-level information concealment and recovery in a FITFET array. Figure 5 shows the array-level demonstration of the voltage-controlled conceal–reveal operation in a 24 × 12 AND-type FITFET array. The array was fabricated using the same gate stack as the single-cell devices to validate large-scale functionality. Figure 5a shows the experimental setup, consisting of a Keysight B1500A semiconductor parameter analyzer and an E5250A switching matrix connected to a 48-pin probe card (Methods). This configuration enables automated addressing of individual cells through flexible routing of word lines (WLs), bit lines (BLs), and source lines (SLs). The *I*_D_-*V*_GS_ and pulsed-based measurements with vector-matrix multiplication (VMM) for the array confirm uniform FITFET behavior consistent with the single-device results (Supplementary Figs. 22 and 23, Note 6)^46,47^. Figure 5b–c summarize the bias schemes for selective program and read operations. High-voltage pulses and inhibit biases are applied to ensure cell selectivity, while a small negative bias on unselected WLs minimizes parasitic read currents. Full bias conditions are provided in Supporting information (Supplementary Fig. 22, Note 7, and Table S1).

The voltage-controlled data concealment of the FITFET array is demonstrated in Fig. 5d. Before image transfer, all cells are fully erased to establish a uniform initial state. A 12 × 12 pixel image is then transferred to the array by tuning conductance of each cell at 0.5 V (plain mode) such that the measured current matched the intended pixel intensity within a 10 % margin (Supplementary Note 8). After transferring, the *V*_read_ was increased from 0.5 to 2.5 V in 0.5 V increments to visualize the concealment dynamics. As *V*_read_ exceeded approximately 1.0 V, the visible features of the stored image gradually disappeared, reflecting a transition into the trap-dominant regime in the *I*_D_-*V*_GS_ characteristics where the FITFET exhibits temporary MW collapse. This voltage-dependent loss of visibility confirms that the same mechanism governing single-device concealment—interfacial trap-induced MW collapse—scales to array operation. However, at higher *V*_read_s (> 2 V), partial reprogramming occurs due to polarization reversal; thus, secure mode should be restricted to this *V*_read_ range to maintain data integrity.

The reversible nature of this concealment is verified in Fig. 5e and supplementary Fig. 24. After image transfer, the programmed conductance map was first read at 1.5 V (secure mode) and then re-read at 0.5 V (plain mode). The image became fully concealed at 1.5 V and was subsequently restored upon returning to 0.5 V, confirming reliable and reversible recovery without degradation of the stored data. Furthermore, array-level disturbance measurements confirm that repeated read operations do not induce unintended state changes in neighboring or unselected cells, demonstrating disturbance-free operation at the array levels (Supplementary Fig. 25). Finally, Fig. 5f provides a visual comparison of the reconstructed images under plain and secure modes. In the plain mode, the array faithfully reproduces the input patterns, whereas in the secure mode the conductance distribution becomes randomized, concealing the stored information from read-out.

Furthermore, the FITFET-based security remains robust even in the presence of minor operational inconsistencies, such as a subset of devices failing to transition into Secure Mode. This resilience is fundamentally rooted in the fact that input information is spatially distributed across a multitude of synaptic weights within the network architecture. Consequently, even if individual weights are partially leaked due to device-level limitations, the incomplete information is insufficient for an adversary to reconstruct the full structural features of the original input. The inherent architectural robustness allows the system to provide reliable data protection while maintaining high operational stability, effectively mitigating the impact of physical device-level non-idealities.

### System level validation of secure neuromorphic computing

Building upon the array-level demonstration of reliable voltage-controlled conceal–reveal behavior, we further investigated whether such device-level and array-level security primitives can be translated into large-scale neuromorphic systems. We examined how the secure mode FITFETs affect learning accuracy, reconstruction vulnerability, and layer-wise sensitivity in neural network inference tasks. System-level simulations confirmed that the reversible MW collapse of FITFETs serves not only as a device-level phenomenon but also as an effective hardware-native security mechanism at algorithmic and architectural levels.

**Fig. 6:**
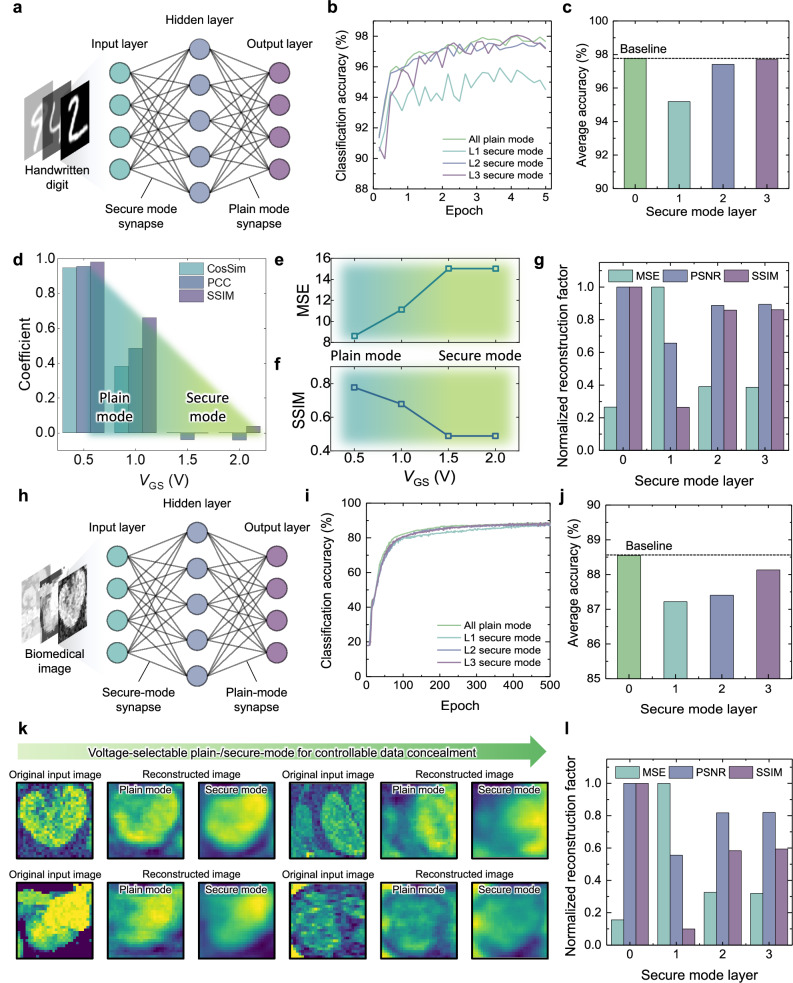
System level validation of privacy preserving neuromorphic computing. **a** Schematic illustration of the FITFET-based neural network architecture for handwritten digit recognition. **b** Evolution of classification accuracy over training epochs and **c** comparison of final average accuracy under different layer-wise concealment conditions, demonstrating a marginal performance trade-off (~3% degradation) induced by securing the first layer (L1) while maintaining high baseline accuracy. **d** Suppression of attack efficacy (coefficients of CosSim, PCC, SSIM), **e** increase in reconstruction error (MSE), and **f** reduction in reconstruction fidelity (SSIM) under varying gate voltages (*V*_GS_), confirming the transition from recognizable plain mode to protected secure mode. **g** Privacy leakage analysis under inversion attacks, evaluating reconstruction feasibility across conceal configurations, with normalized factors identifying L1 as the privacy-critical layer yielding maximum reconstruction error (MSE) and minimum reconstruction fidelity (PSNR, SSIM). **h** FITFET-based neural network architecture adapted for medical image classification to perform extended validation on sensitive biomedical datasets. **i** Classification accuracy evolution and **j** average accuracy at the final training epoch, showing robust learning convergence with negligible performance degradation (<2%) even in secure modes. **k** Visual demonstration of voltage-selectable data concealment, contrasting the plain mode having reconstructible anatomical features with the secure mode inducing significant distortion to render patient data unidentifiable. **l** Normalized reconstruction metrics for individually concealed layers, reaffirming the first layer as the most effective defense against privacy leakage in medical diagnostics.

To quantitatively evaluate this system-level security behavior, pre-trained synaptic weights were transferred to the FITFET array. Figure 6a shows the network architecture of the FITFET-based handwritten digit recognition system (Methods). To ensure a realistic prediction of hardware-level performance, our system-level simulation framework comprehensively incorporates measured device non-idealities, including non-linearity and device-to-device (D2D) variations. Rather than relying on literature-sourced values, all simulation parameters were directly extracted from experimental measurements of the fabricated FITFET devices. Specifically, D2D variations were modeled using a statistical distribution based on the coefficient of variation ($\sigma /\mu$) derived from the measured conductance states (see Supplementary Fig. 18 for details). This measurement-driven approach confirms that the reported recognition accuracies faithfully reflect the actual physical capabilities of the FITFET array under real-world constraints. The baseline recognition performance of the system was evaluated when all layers operated in plain mode without concealment. In addition, the network performance was assessed while selectively activating the secure mode in each layer to examine the impact of information concealment on network performance. As activating the secure mode of the FITFETs inevitably degrades recognition performance, the sensitivity of each layer to performance degradation was analyzed in a layer-wise manner.

Figure 6b shows the classification accuracy of the proposed system as a function of the training epoch for each layer-wise concealment condition. Regardless of the layer-wise concealment status, accuracy consistently increases as training progresses for all evaluated cases. The average accuracy at the final training epoch is presented in Fig. 6c for each layer-wise concealment condition. The baseline accuracy, with all layers operating in plain mode, achieves a high recognition performance of approximately 98%. On the other hand, systems employing layer-wise secure mode FITFETs exhibit slight performance degradation. Significantly, the extent of degradation varies depending on which specific layer of the network is operated in secure mode. Concealing the first layer (L1) caused the most significant accuracy degradation of about 3%, indicating that L1 is the most sensitive layer governing the final inference performance.

Beyond a singular metric like classification accuracy, rigorous quantitative evaluations with a MI attack were conducted to assess the concealing efficiency of the secure mode FITFETs. In the MI attack, the original input images were reconstructed from the trained synaptic weight maps in the FITFET array. The evaluations quantitatively assessed how effectively the trained model was protected against privacy leakage under various perspectives. Various image quality evaluation metrics were employed to measure the similarity and error between the original and reconstructed data. Cosine similarity (CosSim) and Pearson correlation coefficient (PCC) measure the pattern similarity and linear correlation between two data vectors (weight maps), respectively^48^. The structural similarity index measure (SSIM) evaluates the structural similarity between two images, reflecting human visual perception, while the mean squared error (MSE) quantifies the average pixel-wise error. The peak signal-to-noise ratio (PSNR) measures the quality of reconstruction by comparing the maximum possible power of a signal to the power of the distorting noise^49,50^. These metrics enable a comprehensive assessment of the reconstructed image through concealing operations in secure mode FITFETs.

Figure 6d shows the degradation of weight map similarity and image reconstruction fidelity as a function of the applied *V*_GS_. As *V*_GS_ increases beyond 1.0 V, the CosSim, PCC, and SSIM values all significantly decrease to near zero, indicating that the original information is effectively obscured and the efficacy of reconstruction attacks is degraded. Figure 6e–f shows that the MSE value rapidly increases as *V*_GS_ increases. In contrast, the SSIM value decreases, empirically confirming the increasing dissimilarity between the original and concealed weight maps induced by the secure mode FITFETs.

Furthermore, the vulnerability of each layer to an MI attack was analyzed to identify which layers contained the most critical information for image reconstruction. Figure 6g shows the normalized MSE, PSNR, and SSIM values when each layer was selectively operated in secure mode. The results indicate that activating the secure mode in any layer increases reconstruction error and reduces similarity metrics, confirming the effectiveness of the concealment in suppressing image reconstruction. The concealing operation was most effective for L1, followed by L2 and L3. Concealing L1 rendered the image reconstruction most inefficient, as evidenced by the highest MSE and lowest PSNR and SSIM values. These results indicate that L1 contains the most critical information for image reconstruction, and concealing this layer provides the most effective security. The analysis quantitatively verifies the efficacy of the proposed secure mode FITFETs in protecting against image reconstruction attacks.

As the most effective layer for security is also the most sensitive to performance degradation, a clear performance-security trade-off exists. Notably, although concealing L1 provides strong protection, the associated classification accuracy degradation is relatively minor—about 3% (Fig. 6c)—and thus negligible in the context of high-security applications. Therefore, a robust FITFET-based hardware security system can be realized with minimal performance overhead. Furthermore, the proposed layer-wise strategy offers design flexibility, enabling system designers to select or combine layers for concealment according to the desired protection level and acceptable performance margin.

Unlike traditional frameworks that require active authentication to distinguish between users, the FITFET-based system offers a ‘Safe-by-Design’ approach that eliminates the need for a complex, real-time security check workflow. This workflow can be achieved by adjusting the relative *V*_SL_ and *V*_BL_ voltages to permanently shift the operating window of sensitive layers (e.g., L1) into Secure Mode (*V*_GS_ ≈ 1.25 − 1.5 V). Such a fixed hardware configuration ensures that synaptic weights remain physically concealed from any adversarial reconstruction for all users, while maintaining high-fidelity authorized inference with negligible computational overhead.

Furthermore, the FITFET array is highly robust against physical adversarial manipulation due to significant information asymmetry regarding the system architecture; a potential attacker lacks critical knowledge of the network size, layer structure, and particularly the exact voltage difference between Plain and Secure Modes. The security is further reinforced by modulating the relative *V*_SL_ and *V*_BL_ voltages, which allows the system to shift the operating window and effectively present a ‘moving target’ for any brute-force attempt. Crucially, such indiscriminate voltage manipulation outside the calibrated range carries the inherent risk of triggering the destructive erasure or perturbation of ferroelectric and ionic states, thereby physically self-protecting the stored information from being compromised.

To validate the generality of the proposed security system, its application was extended to a dataset comprising sensitive standardized biomedical images (Fig. 6h). Figure 6i shows the classification accuracy on the sensitive, real-world medical dataset as a function of the training epoch under each layer-wise concealment condition. Consistent with the handwritten digit recognition results, the accuracy increases as training progresses for all cases. The average accuracy at the final training epoch for each condition is shown in Fig. 6j. These results confirm the earlier findings: the baseline accuracy remains high, whereas systems employing layer-wise secure mode exhibit only marginal performance degradation (within 2%). Notably, L1 again appears as the most sensitive layer, causing the largest accuracy drop when concealed. Figure 6k illustrates the effectiveness of the voltage-selectable plain and secure modes in achieving controllable data concealment. A direct comparison is provided between the original input medical images, such as chest X-rays, and the images reconstructed from the plain mode and secure mode weights. Unlike the plain mode, images reconstructed from the secure mode weights appear blurred and distorted, rendering them effectively unrecognizable. Figure 6l shows the normalized MSE, PSNR, and SSIM values obtained from the reconstruction attack scenario, where each layer was individually concealed. These metrics quantitatively verify the effectiveness of the proposed secure mode FITFETs in resisting image reconstruction attacks, as indicated by high MSE and low PSNR and SSIM values. Consistent with the handwritten digit recognition results, the concealing operation was most pronounced for L1, reaffirming it as the most effective layer for protection. These results demonstrate that the unique physical operating principle of the FITFET—namely, the reversible MW collapse—serves as a scalable hardware security primitive.

Through comprehensive quantitative analyses, the proposed FITFET-based security system provides a robust and effective safeguard for the sensitive personal information implicitly embedded in the input data. By preventing the leakage of such sensitive information at the hardware level, this approach establishes a foundation for reliable and privacy-preserving AI systems, particularly in security-critical applications such as medical diagnostics. As summarized in Supplementary Table 2, the unique combination of voltage-tunable synaptic and trap-mediated data concealment of FITFET distinguishes it from conventional filament-based memories, offering CMOS-compatible platform where computation and protection coexist within the single device^51–56^ (Supplementary Note 9).

In this study, we introduced a FITFET as a unified device platform that combines high-fidelity neuromorphic computation with hardware-native data protection. By integrating a thin SiO_2_/TiO_2_ interfacial stack with an HZO ferroelectric layer, the FITFET exploits a coupled ferro-ionic–trapping complex that is selectively addressable by gate bias. In this architecture, the TiO_2_ interlayer functions as a multifunctional interface that simultaneously maintains effective ferroelectric-to-channel coupling at low bias and enables bias-programmable charge trapping for non-destructive memory concealment. This engineered stack yields three distinct operating regimes—ferroelectric switching, trap-mediated memory-window collapse, and oxygen-vacancy–driven ionic switching—enabling a bias-programmable synapse with a reversible concealable readout. Stored synaptic weights can thus be physically hidden under secure mode read conditions and fully recovered in plain mode, without destructive erasure or endurance penalties. Multiscale characterization, from atomic-resolution imaging and defect profiling to LFN spectroscopy, validates that the unconventional open–collapse–reopen memory behavior originates from deliberate interface and defect engineering. Scaling this mechanism to a FITFET array, we demonstrate voltage-controlled conceal–reveal of stored images, and system-level simulations with experimentally calibrated devices show that FITFET-based synaptic arrays can significantly suppress MI attacks on both benchmark and medical datasets with only minor accuracy loss. Beyond demonstrating a specific device, this work reframes ferroelectric, ionic, and trap-mediated phenomena in hafnia-based stacks—from parasitic nonidealities into co-engineered degrees of freedom—establishing a CMOS-compatible route toward intrinsically secure, energy-efficient, and privacy-preserving neuromorphic hardware.

## Methods

### Fabrication of devices

We engineered hafnia-based transistors that progress from a FeFET to a FIDFET and culminate in the proposed FITFET. All devices were fabricated on 6-inch SOI substrate with a 100 nm-thick buried oxide using a gate-first CMOS-compatible process. The active regions were defined by photolithography and dry etching, followed by sequential surface cleaning with piranha solution (SPM), standard clean-1 (SC-1), and standard clean-2 (SC-2). Ferroelectric gate stacks of SiO_2_/HZO (FeFET), SiO_2_/TiO_2_/HZO (FIDFET and FITFET) were deposited by atomic layer deposition (ALD) at 330 °C using BDEAS, TDMAT, TEMA-Hf, TEMA-Zr and O_3_ as precursors and oxygen sources. The HZO composition was controlled by adjusting the cycle ratio of HfO_2_ and ZrO_2_, employing two cycles of HfO_2_ followed by one cycle of ZrO_2_. Each device incorporated a 6.4 nm HZO ferroelectric layer, while the interfacial stack thicknesses were varied to define three configurations: the FeFET employed a 1.73 nm SiO_2_ interlayer, the FIDFET adopted a 1.92 nm SiO_2_-TiO_2_ bilayer, and the FITFET utilized a thinner 1.33 nm SiO_2_-TiO_2_ bilayer to achieve modulated interface coupling. TiN was sputtered as the gate electrode and patterned by photolithography and reactive ion etching. Source and drain regions were formed by self-aligned phosphorus ion implantation at a dose of 10^15 ^cm^−2^ with 20 keV acceleration energy. Post-metallization annealing (PMA) was performed at 650 °C for 30 s in nitrogen to activate dopants and crystallize the ferroelectric HZO layer. A 300 nm SiO_2_ passivation layer was subsequently deposited by plasma-enhanced chemical vapor deposition (PECVD) and contact vias were opened by RIE. Finally, Ti/TiN/Al/TiN metal stacks were deposited by DC sputtering to form contact pads. The fabricated transistors were structurally and chemically characterized by cross-sectional TEM and EDS mapping (Supplementary Figs. 2 and 3)

### Grazing incidence X-ray diffraction

GIXRD measurements were performed using a high-resolution diffractometer (X’Pert-Pro MRD Phillips) operated at a fixed incident angle of 0.5°, enabling enhanced surface sensitivity for thin-film characterization. The resulting diffraction profiles were examined to distinguish the major crystalline phases—orthorhombic, tetragonal, and monoclinic—and to estimate their relative proportions. These evaluations elucidated the phase composition of the HZO films, offering direct evidence of the ferroelectric phase stability under different interface configurations.

### Electron energy loss spectroscopy

EELS was carried out using a Thermo Fisher Spectra Ultra scanning transmission electron microscope (STEM) equipped with dual Cs correctors for both imaging and probe formation, operated at an accelerating voltage of 300 kV. Low-loss and core-loss spectra were recorded with an energy dispersion of 0.25 eV to ensure high spectral resolution. Quantitative oxygen mapping and energy loss near edge structure (ELNES) analyses of the O-K edge were conducted using Gatan Microscopy Suite 3 (GMS 3) in accordance with the procedure reported by Verbeeck and Van Aert^57^. A power-law model was applied for background subtraction, and Hartree-Slater cross-sections were used to extract oxygen content. The intensity ratio between the *p*_1_ and *p*_2_ peaks of the O-K edge was subsequently evaluated to track spatial variations in V_O_ concentration across the HZO films.

### X-ray photoelectron spectroscopy

XPS was performed using a Thermo Fisher Scientific NEXSA system to characterize the chemical states and V_O_ profiles in the hafnia-based transistors. Depth-resolved XPS spectra were obtained to examine how the interfacial oxygen stoichiometry varies depending on both the presence of the TiO_2_ interlayer and the SiO_2_ thickness modulation. By analyzing the O 1 s core-level spectra, the oxide-deficient components were quantitatively extracted, revealing how the interfacial state evolves from the FeFET to the TiO_2_-interfaced FIDFET and the thinned SiO_2_–TiO_2_ FITFET. This comparative analysis provided direct evidence that interlayer engineering simultaneously regulates V_O_ distribution and interface defect density across the three device architectures.

### Electrical measurement setup

The comprehensive measurement configuration used for electrical characterization is illustrated in Fig. 5a. Ferroelectric switching behaviors of the fabricated transistors were characterized using a probe station integrated with a Keithley 4200-SCS semiconductor parameter analyzer. The DC transfer characteristics (*I*_D_–*V*_GS_s) were measured with a Keysight B1500A system equipped with precision source measurement units (SMUs). For pulse-dependent and high-speed transient measurements, a B1500A analyzer coupled with a waveform generator/fast measurement unit (B1530A, WGFMU) and a high-voltage semiconductor pulse generator unit (B1525A, SPGU) was employed. Vector–matrix multiplication (VMM) operations were evaluated under the same setup to ensure consistent bias control and timing synchronization. For large-scale array characterization, the B1500A was interfaced with a Keysight E5250A switching matrix connected to a custom-designed 48-pin probe card. In this configuration, the SMU and WGFMU outputs served as the input channels to the switching matrix, while the 48 routed outputs were assigned to the 24 word lines (WLs), 12 BLs, and 12 SLs, enabling full 24 × 12 array operation. This structure allowed flexible routing of multiple biasing signals to any selected cell within the array. Device addressing, signal sequencing, and automated bias control were implemented through LabVIEW-based custom scripts in conjunction with Keysight EasyExpert software, allowing programmable PGM/ERS/READ operations and synchronized array-level measurements.

### Low frequency noise

LFN spectra were acquired with a Keysight B1500A to bias *V*_GS_ and *V*_DS_ while monitoring the source current through a low-noise current preamplifier (Stanford Research Systems SR570). The source node was grounded through SR570; drain current fluctuations were converted to voltage, bandwidth-limited by the preamplifier’s built-in low-pass filter and analyzed in the frequency domain with a dynamic signal analyzer (Keysight 35670 A) to obtain PSD. To suppress slow drifts and background offsets, SR570 was operated in Low-Drift mode, and its input-offset current was used to null DC leakage; the unit’s internal bias output and configurable low/high-pass filters (0.03 Hz–1 MHz) facilitated stable transimpedance readout under varying source capacitances. Note that SR570 was run from its internal battery supply to mitigate ground loops. Bias-dependent noise behavior was mapped by changing *V*_GS_ across the device operating regimes and holding each setpoint until the current stabilized, thereby isolating signatures associated with ferroelectric switching (plain mode), interface-trap–induced memory-window collapse engaged by elevated read bias (secure mode), and V_O_-mediated ionic switching. In this way, variations in the 1/*f* noise and Lorentzian components of the PSD are traced to the operative mechanisms without perturbing the device state between measurements.

### System level simulations

All system-level simulations were performed using a custom framework developed in Python. This framework utilized PyTorch to define and train the neural network models, and NumPy was used for numerical analysis and data processing.

#### Datasets

The MNIST and MedMNIST datasets^58,59^, each consisting of 28 × 28 grayscale images, were used for the handwritten digit and medical image recognition tasks, respectively. A 784 × 500 × 150 × 10 fully connected three-layer network was used for both the MNIST and MedMNIST dataset recognition tasks.

Model training and synaptic weight mapping: The network models were first pre-trained on each dataset in software using a standard Adam optimizer. The trained synaptic weights were transferred to the conductance of the fabricated FITFET array.

#### Inference process

To evaluate inference performance, the test set from each dataset was fed into the trained network. The classification accuracy was first obtained from plain-mode FITFET array. To analyze the performance-security trade-off, the FITFET array of each layer (L1, L2, L3) was individually operated in secure-mode, and the corresponding degradation in accuracy was evaluated.

#### Hardware security capability (MI attack)

To evaluate the security capability of the proposed FITFET-based neuromorphic system, the MI attack was conducted. The MI attack aims to reconstruct the original input images from the stored model weights. The attack was implemented using ridge regression and performed in two states: plain mode using the original weights, and secure mode with a specific layer concealed.

### Quantitative evaluation metrics

#### Weight obscuration assessment

To evaluate how effectively the secure mode obscures the weights themselves, the original weight map and the concealed weight map were compared by using CosSim and PCC.

#### Protection performance assessment

The final protection performance against the attack was evaluated by comparing the original input image with the reconstructed image. The assessment included the image quality metrics MSE, PSNR, and SSIM.

The calculation formulas for the five key quantitative metrics used in this study are as follows.

#### CosSim

Measures the similarity of patterns between two vectors X and Y by calculating the cosine of the angle between them.

1 $${{{\rm{CosSim}}}}\left({{{\rm{A}}}},{{{\rm{B}}}}\right)=\frac{{{{\rm{X}}}}\cdot {{{\rm{Y}}}}}{\left|{{{\rm{X}}}}\right|\left|{{{\rm{Y}}}}\right|}=\frac{{\sum }_{{{{\rm{i}}}}=1}^{{{{\rm{n}}}}}{x}_{i}{y}_{i}}{\sqrt{{\sum }_{{{{\rm{i}}}}=1}^{{{{\rm{n}}}}}{x}_{i}^{2}}\sqrt{{\sum }_{{{{\rm{i}}}}=1}^{{{{\rm{n}}}}}{y}_{i}^{2}}}$$

PCC (r): Measures the linear correlation between two vectors X and Y, where ${\mu }_{X}$ and ${\mu }_{Y}$ are the mean values of *X* and *Y*, respectively.

2 $$r=\frac{n{\sum }_{{{{\rm{i}}}}=1}^{{{{\rm{n}}}}}\left({x}_{i}{y}_{i}\right)-\left({\sum }_{{{{\rm{i}}}}=1}^{{{{\rm{n}}}}}{x}_{i}\right)\left({\sum }_{{{{\rm{i}}}}=1}^{{{{\rm{n}}}}}{y}_{i}\right)}{\sqrt{n{\sum }_{{{{\rm{i}}}}=1}^{{{{\rm{n}}}}}{x}_{i}^{2}-{\left({\sum }_{{{{\rm{i}}}}=1}^{{{{\rm{n}}}}}{x}_{{{{\rm{i}}}}}\right)}^{2}}\sqrt{n{\sum }_{{{{\rm{i}}}}=1}^{{{{\rm{n}}}}}{y}_{i}^{2}-{\left({\sum }_{{{{\rm{i}}}}=1}^{{{{\rm{n}}}}}{y}_{i}\right)}^{2}}}$$

MSE: Calculates the average of the squared errors between the *n*-th sample value of the original image $u({x}_{n})$ and the *n*-th sample value of the test image $v({x}_{n})$, over *M* total pixels.

3 $${{{\rm{MSE}}}}=\frac{1}{{{{\rm{M}}}}}{{\sum }_{n=1}^{M}[u({x}_{n})-v({x}_{n})]}^{2}\,$$

PSNR: Based on MSE, represents the ratio of the maximum signal (maximum possible pixel value ${MA}{X}_{I},$ e.g., 255) to the noise (error) on a logarithmic decibel (dB) scale.

4 $${{{\rm{PSNR}}}}=10\cdot {\log }_{10}\left(\frac{{MA}{X}_{I}^{2}}{{MSE}}\right)$$

SSIM: Compares the luminance (μ), contrast (σ), and structure (${\sigma }_{{xy}}$) of two images, x and y. ${C}_{1}$ and ${C}_{2}$ are constants for stability.

5 $${{{\rm{SSIM}}}}\left(x,y\right)=\frac{\left(2{\mu }_{x}{\mu }_{y}+{C}_{1}\right)\left(2{\sigma }_{{xy}}+{C}_{2}\right)}{\left({\mu }_{x}^{2}+{\mu }_{y}^{2}+{C}_{1}\right)\left({\sigma }_{x}^{2}+{\sigma }_{y}^{2}+{C}_{2}\right)}$$

## Supplementary information

Supplementary Information

Transparent Peer Review file

## Source data

Source Data

## Data Availability

The data that support the findings of this study are available in the Supporting Information of this article. The data generated in this study are provided in Source Data files. All other data is available from the corresponding author upon request. Source data are provided with this paper.

## Code availability

All relevant codes used for the simulation are available in the article and Supplementary Information. All other codes are available from the corresponding authors on request.
