# Supplementary material for: A ferroelectric-ionic-trapping transistor for low power and secure neuromorphic computing
Source: Nat Commun. 2026 May 29;17:7831. doi: 10.1038/s41467-026-72971-y (PMC13439170; doi:10.1038/s41467-026-72971-y)
Supplement: Supplementary file 1 [file 41467_2026_72971_MOESM1_ESM.pdf]

A ferroelectric-ionic-trapping transistor for ultra-low power and secure neuromorphic computing

Changhyeon Han[1,†], Youngchan Cho[2,†], Dongbin Kim[1,†], Se-Hyun Hwang[3,†], Minsuk Song[1], Been Kwak[1], David Radermacher[2], Min Wook Kang[4], Sangwan Kim[4], Jangsaeng Kim[3,5,6,], Wonjun Shin[2,4,]*, Daewoong Kwon[1,]**

[1]Department of Electrical Engineering, Hanyang University, Seoul 04763, Republic of Korea

[2]Department of Semiconductor Convergence Engineering, Sungkyunkwan University, Suwon 016419, Republic of Korea

[3]Department of Semiconductor Engineering, Sogang University, Seoul 04107, Republic of Korea

[4]Department of Electrical and Computer Engineering, Sungkyunkwan University, Suwon 016419, Republic of Korea

[5]Department of Electronic Engineering, Sogang University, Seoul 04107, Republic of Korea

[6]Department of System Semiconductor Engineering, Sogang University, Seoul 04107, Republic of Korea

C. Han, Y. Cho, D. Kim, and S.-H. Hwang contributed equally to this work.

* E-mail: kimjs@sogang.ac.kr, swj0107@skku.edu, dw79kwon@hanyang.ac.kr

Contents

Supplementary Figure 1–25

Supplementary Table 1–2

Supplementary Note 1–9

Supplementary Reference 1–11

Supplementary Figures

Supplementary Figure 1. Fabrication process flow for transistors with different interfacial layer configurations

Schematic illustration of the fabrication process flow for the hafnia-based transistors (FeFET, FIDFET, and FITFET). The interfacial layer thicknesses are SiO_2 1.73 nm (FeFET), SiO_2–TiO_2 1.92 nm (FIDFET), and thin SiO_2–TiO_2 1.33 nm (FITFET).

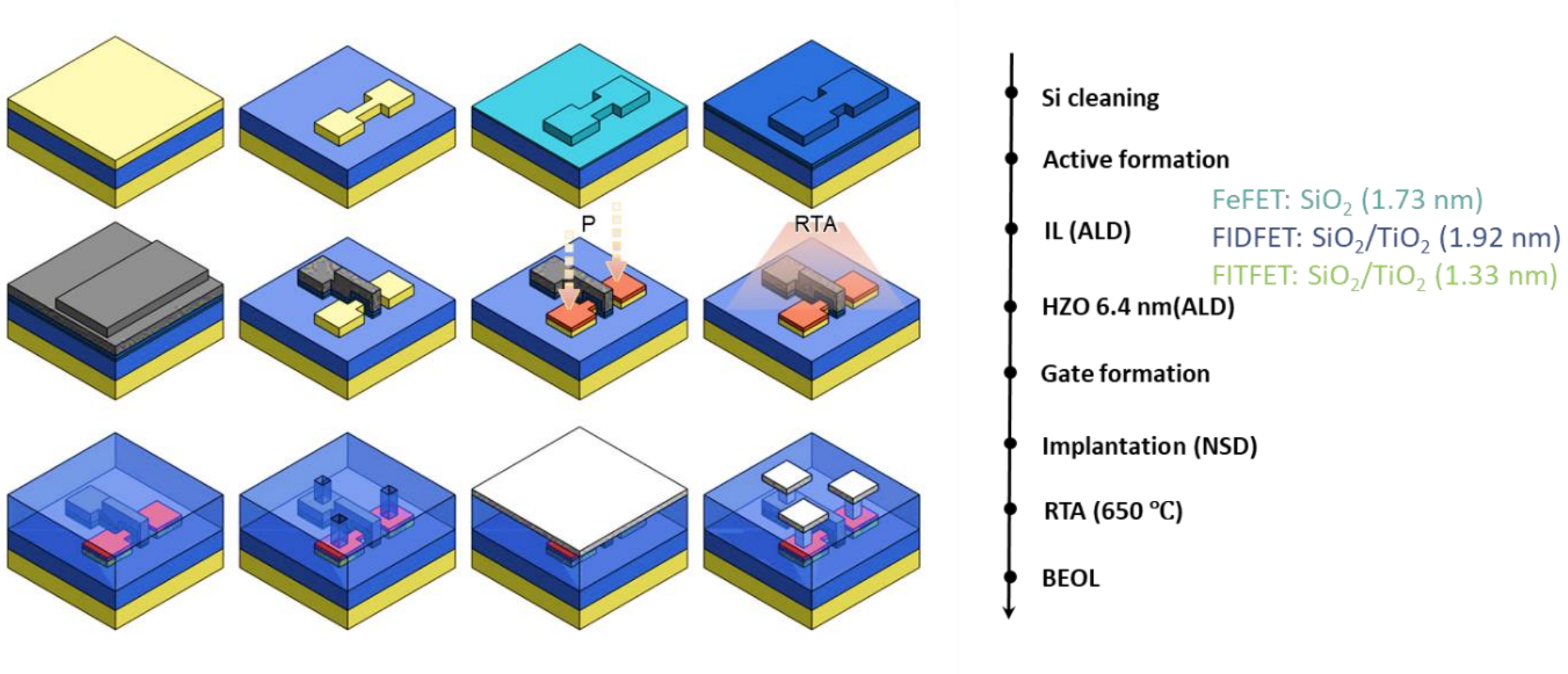

Supplementary Figure 2. Cross sectional EDS elemental mapping of the transistors

Elemental distributions (Si, Hf, Zr, O, Ti) confirm well-defined HZO (6.4 nm) layers over SiO_2–TiO_2 or SiO_2 interlayers with respective thicknesses of 1.92 nm (FIDFET, **a**), 1.33 nm (FITFET, **b**), and 1.73 nm (FeFET, **c**). The clear Ti signal in FIDFET and FITFET verifies the formation of the TiO_2 interlayer, while uniform Hf, Zr, and O signals indicate consistent HZO composition across all devices.

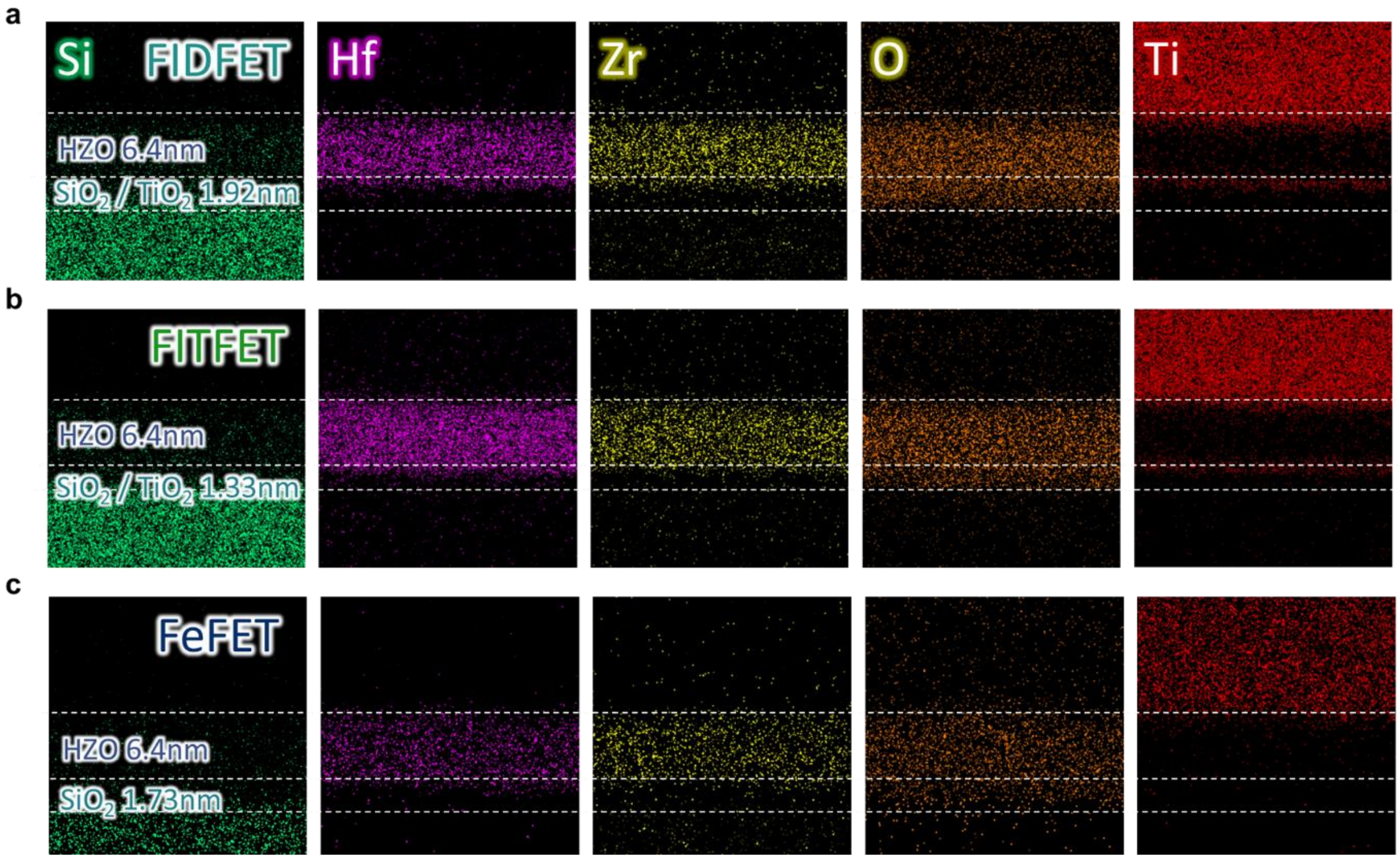

Supplementary Figure 3. Cross sectional TEM and FFT images of transistors

FFT analysis of the lattice fringes (d-spacing ≈ 2.9 Å) confirms the presence of the ferroelectric o-phase in all three types of transistors.

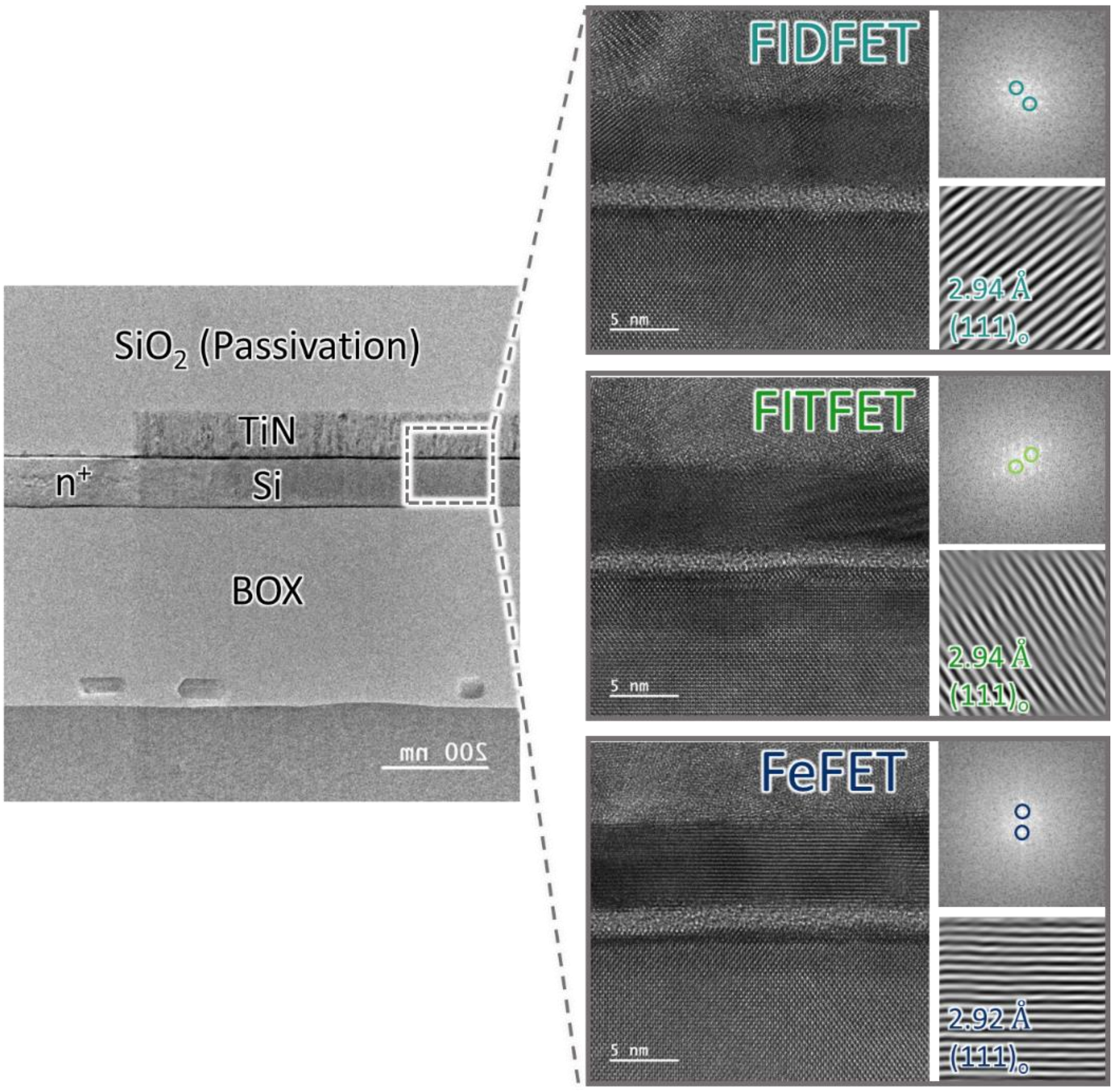

Supplementary Figure 4. GIXRD analysis of transistors

GIXRD patterns of the **a** FIDFET, **b** FITFET, and **c** FeFET structures. The fitted diffraction peaks corresponding to the $(111)_o$, $(011)_t$, and $(111)_m$ planes confirm the coexistence of orthorhombic, tetragonal, and monoclinic phases in all devices. The comparable orthorhombic phase fractions indicate that the insertion of TiO_2 and variation in interfacial layer thickness do not significantly affect the overall crystallinity of the HZO films.

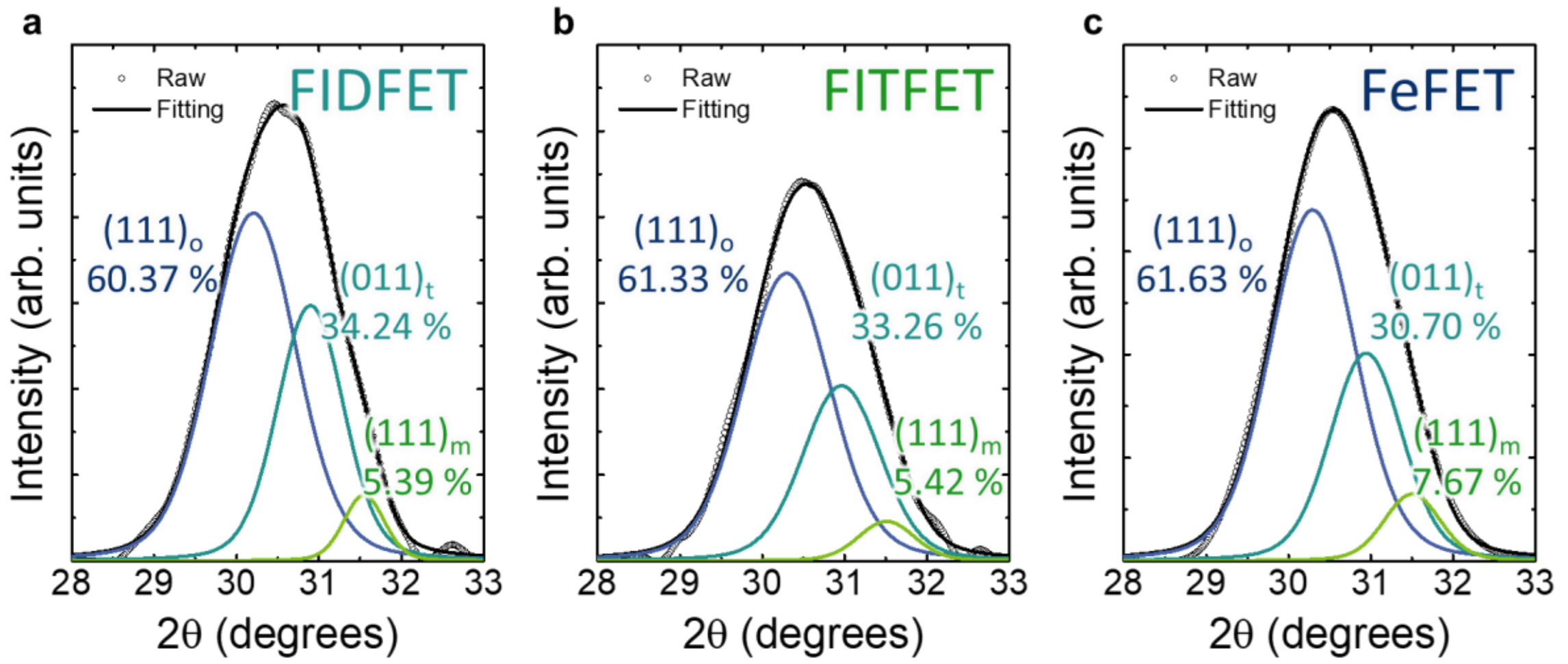

Supplementary Figure 5. STEM and EELS oxygen mapping and analysis of transistors
Cross-sectional STEM-EELS oxygen maps and O-K edge spectra for **a** FIDFET, **b** FITFET, and **c** FeFET. The maps highlight oxygen distribution across the gate stack, and the O-K spectral variations indicate differences in V_O concentration induced by interfacial layer engineering.

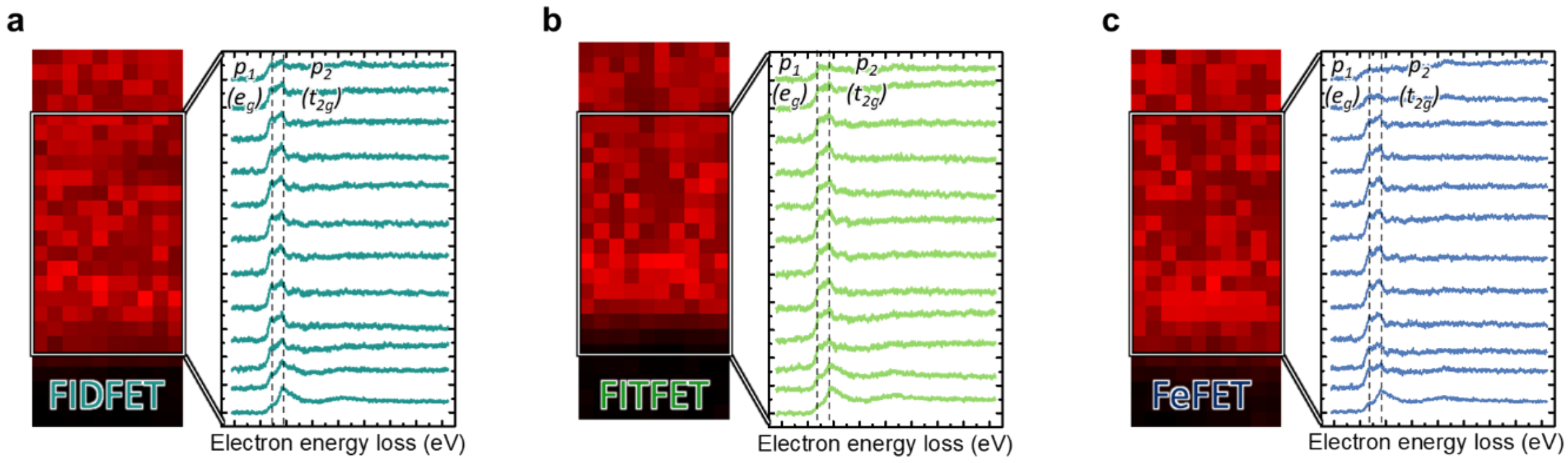

Supplementary Figure 6. Spatial distribution of V_O related O-K edge features

Spatial profiles of the p_1/p_2 intensity ratio extracted from O-K edge EELS spectra. A pronounced reduction in the p_1/p_2 ratio is observed in FIDFET and FITFET compared with FeFET, confirming localized V_O accumulation in the entire HZO layer.

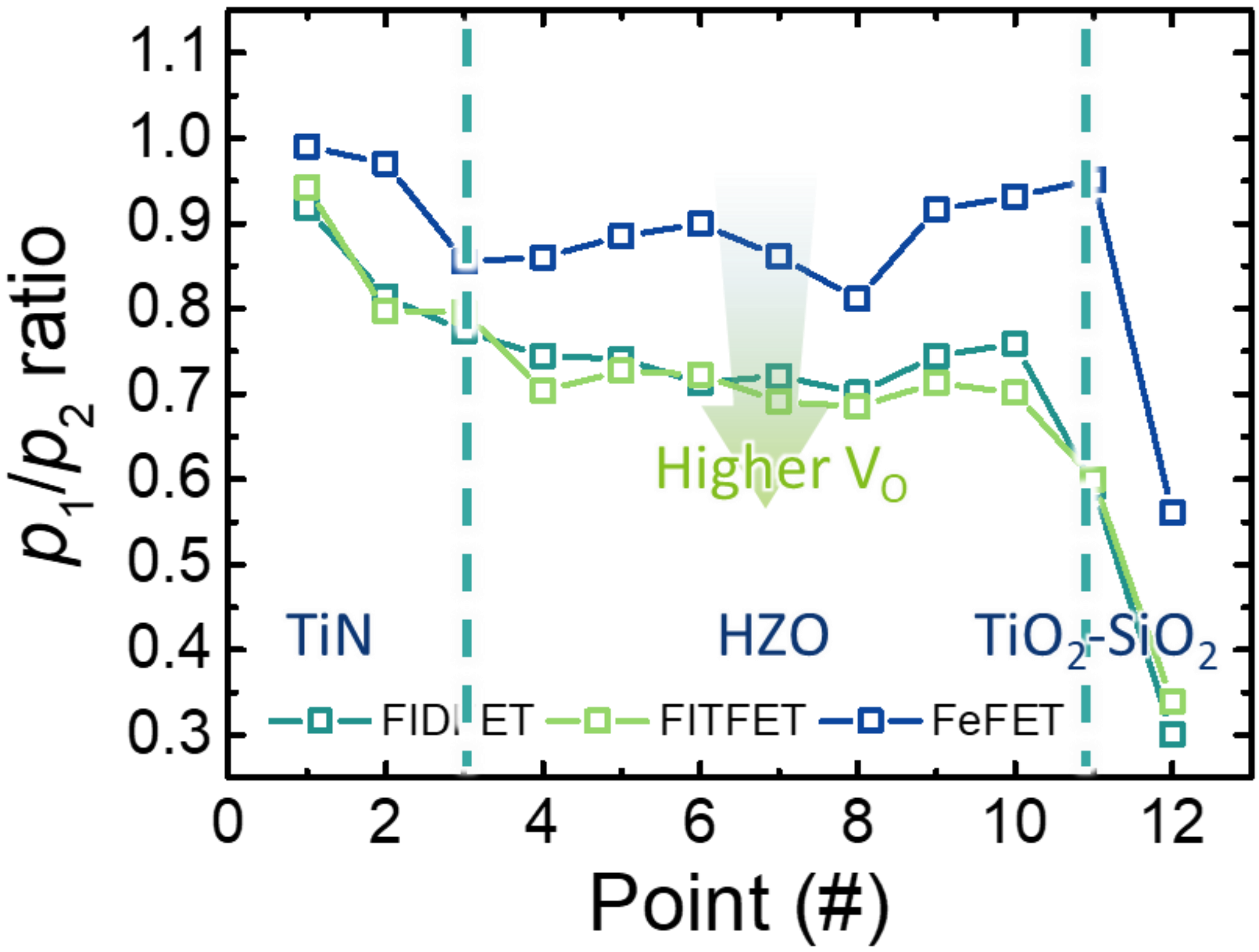

Supplementary Figure 7. XPS analysis of V_O profiles in transistors

XPS O 1s spectra for **a** FIDFET, **b** FITFET, and **c** FeFET reveal the relative fraction of V_O-related components. **d** Depth-dependent V_O ratio extracted from XPS depth profiling shows that both FIDFET and FITFET exhibit higher V_O concentrations across the gate stack compared to FeFET, confirming that the TiO_2 interlayer induces increased oxygen deficiency.

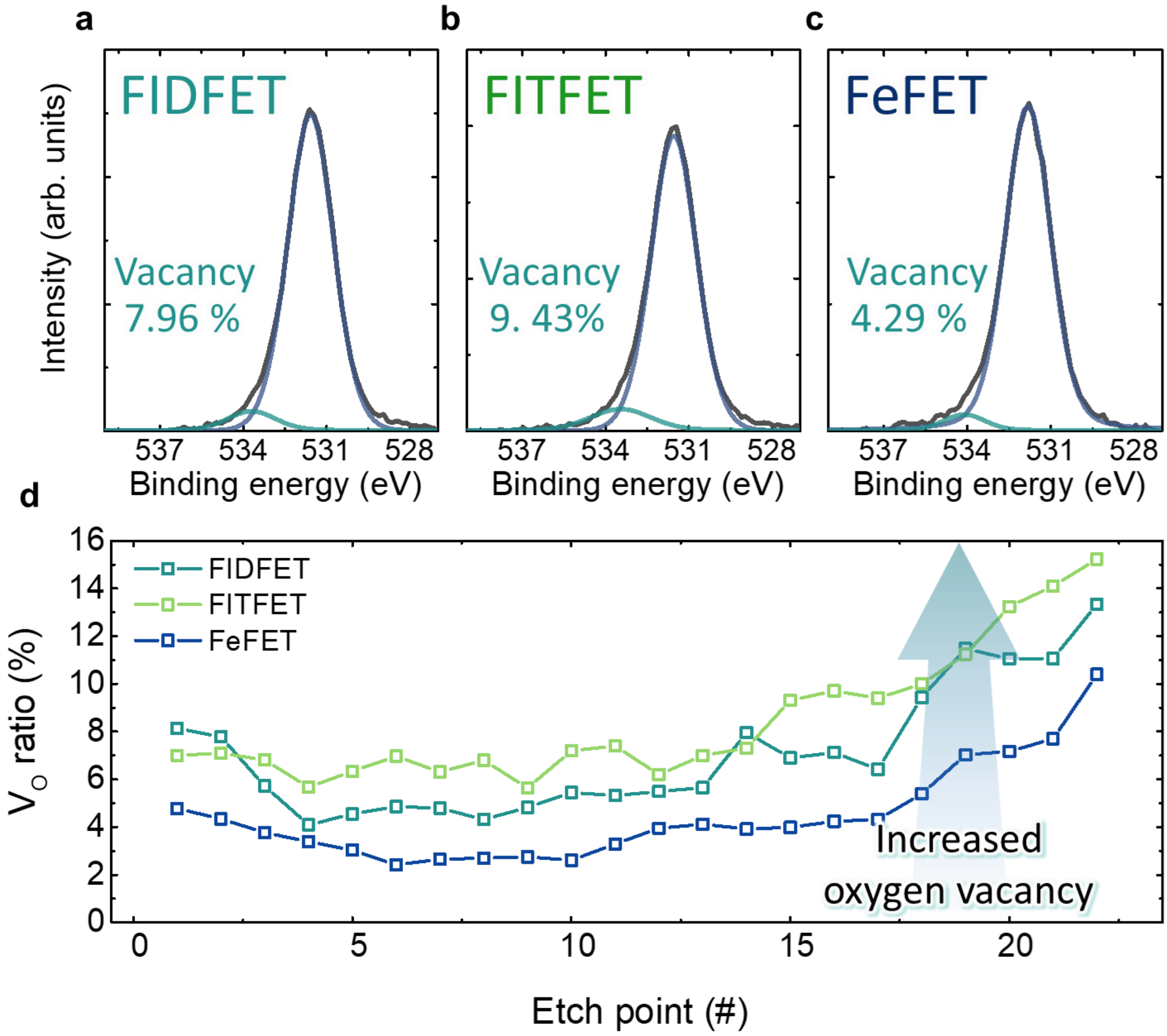

Supplementary Figure 8. Bias-dependent I_D-V_{GS} characteristics of the FeFET

I_D-V_{GS} curves measured under **a** 2–3 V, **b** 3.2–4.2 V, and **c** 4.4–4.8 V, respectively. **d** Extracted MW evolution as a function of V_{GS}, showing that MW appears above ~3.5 V as ferroelectric switching is triggered, followed by trap-driven MW degradation at higher V_{GS}.

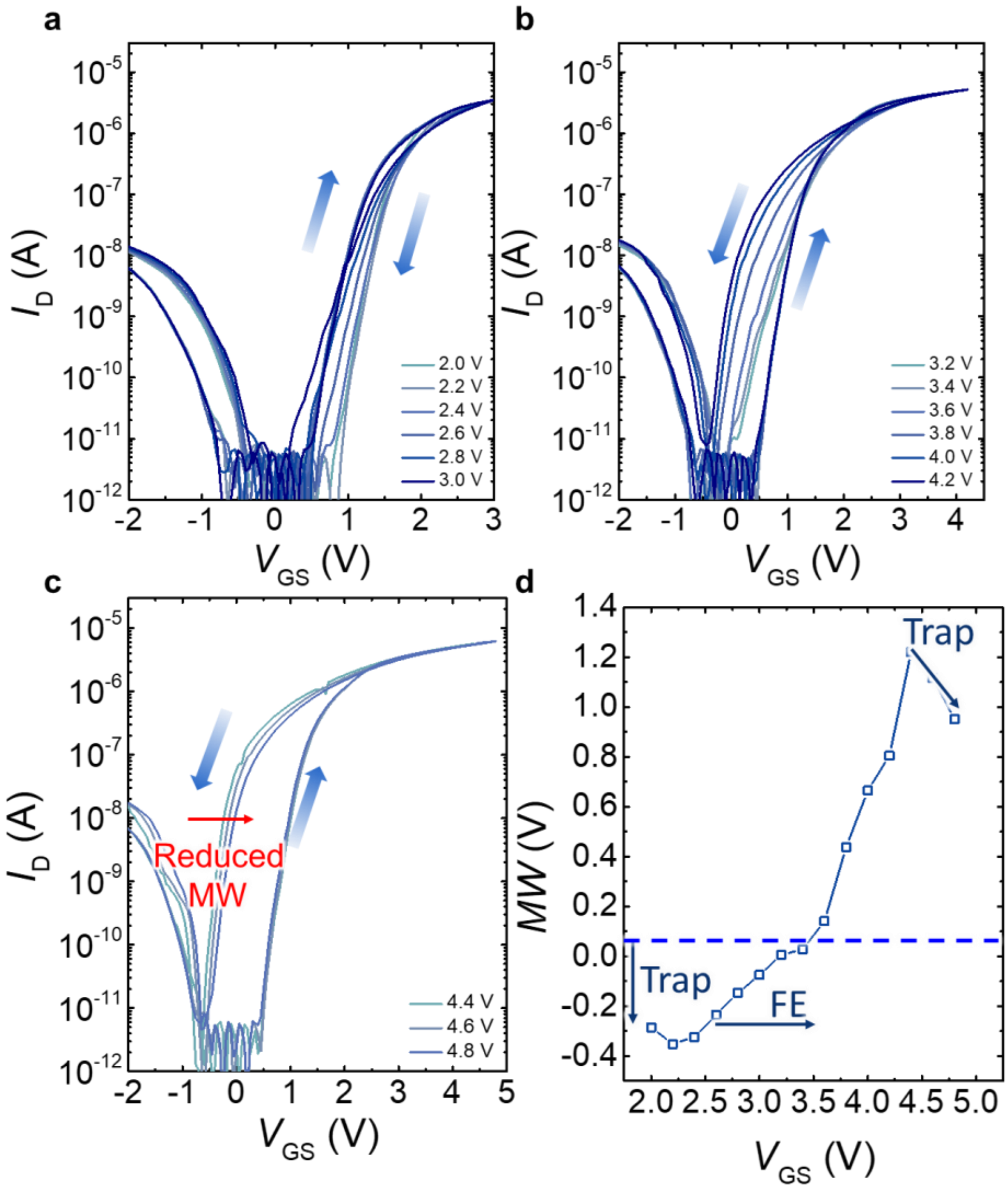

Supplementary Figure 9. Bias dependent I_G-V_{GS} transfer characteristics of the FITFET

I_G-V_{GS} curves of the FITFET measured under varying positive voltages: **a** 1–2 V, **b** 2–4 V, and **c** 4–5 V. At low bias, distinct polarization switching peaks are observed, indicating ferroelectric dipole reversal within the HZO layer. At higher V_{GS}, a steady rise in I_G indicates field-driven ionic migration and the onset of ferro-ionic hybrid conduction.

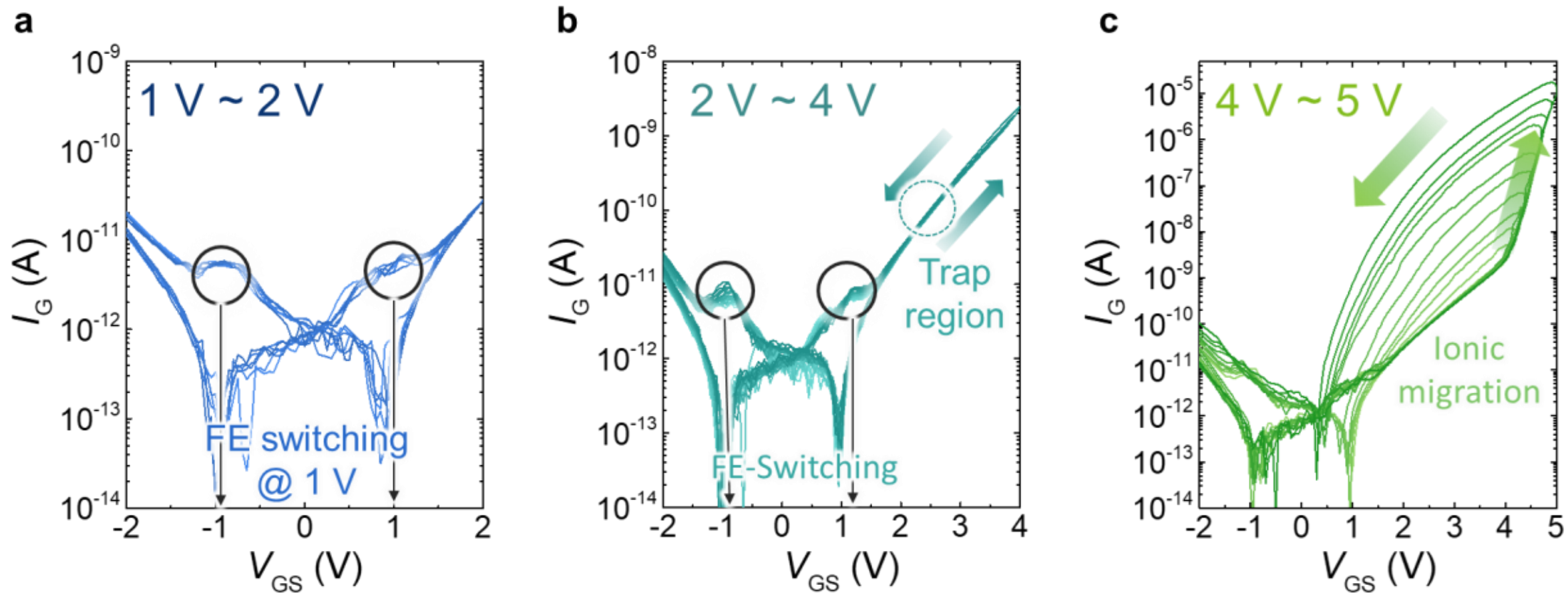

Supplementary Figure 10. Transfer characteristics under V_{GS} conditions of transistors

Transfer characteristics of **a** FeFET, **b** FIDFET, and **c** FITFET measured under low- and high-V_{GS} conditions. The FeFET exhibits purely ferroelectric switching, the FIDFET shows combined ferroelectric and ionic responses, and the FITFET shows ferroelectric, trap-mediated screening, and ionic contributions depending on V_{GS}, reflecting the distinct interfacial layer configurations of the three devices.

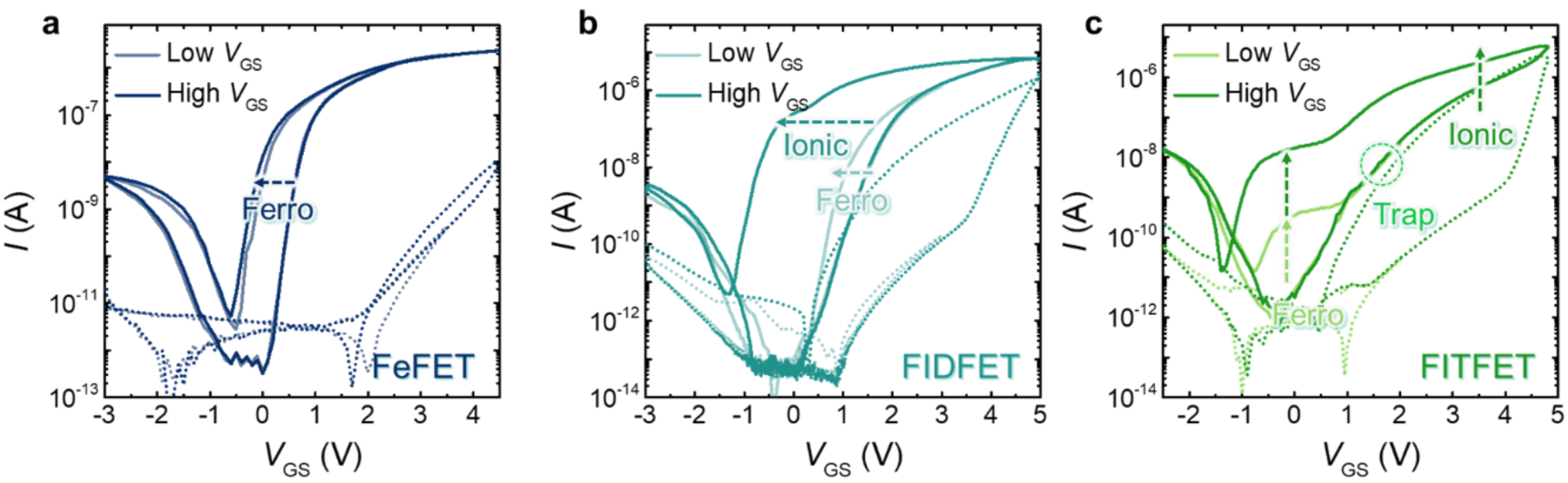

Supplementary Figure 11. LFN characteristics of control SiO_2 HZO FeFET.

a S_{ID}/I_D^2 and **b** $f \times S_{ID}/I_D^2$ versus frequency of the control SiO_2/HZO FeFET with a thicker SiO_2 interlayer and without the TiO_2 insertion.

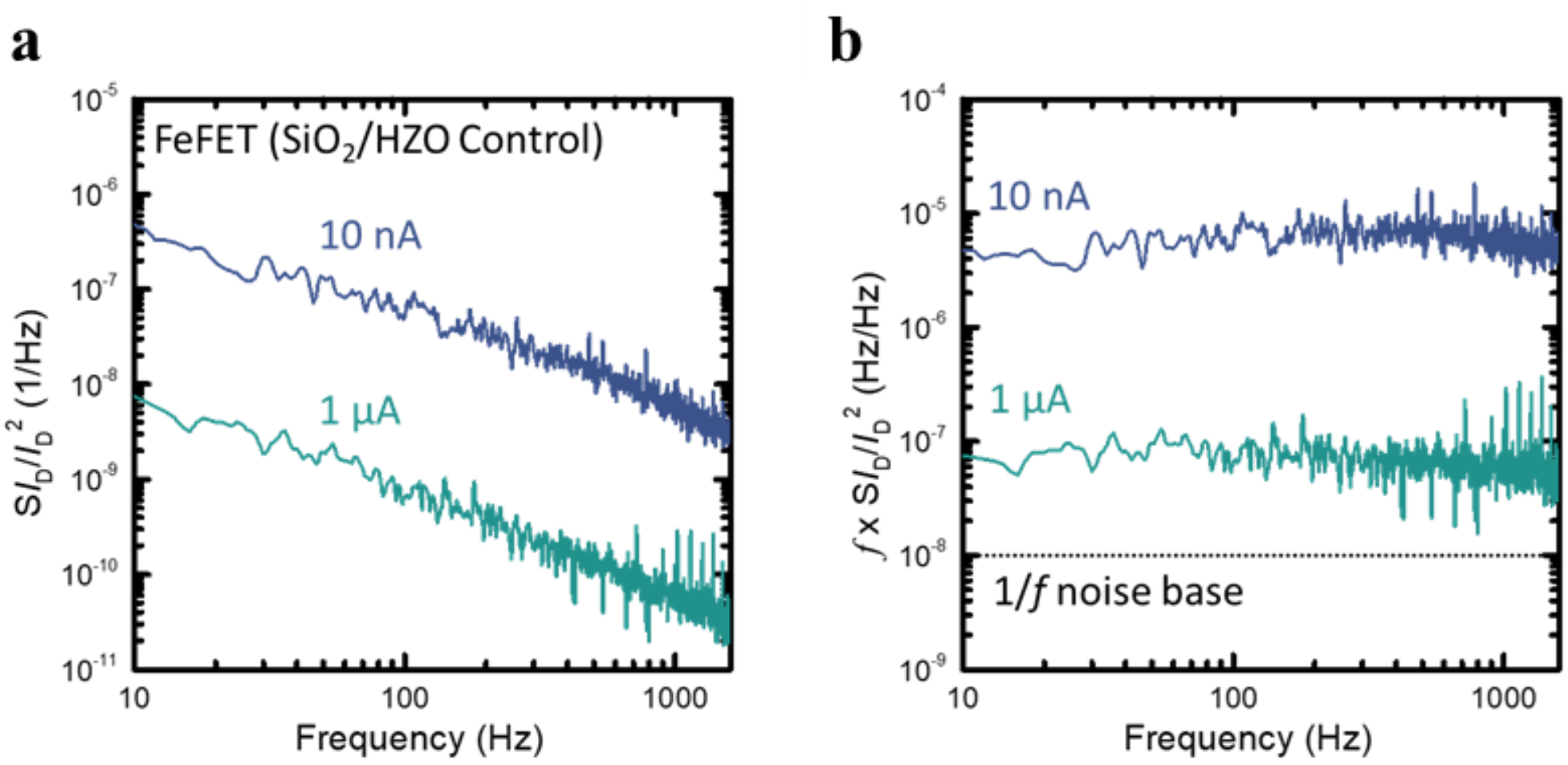

Supplementary Figure 12. S_{ID}/I_D^2 sampled at 10 Hz and constant × $(g_m/I_D)^2$ versus I_D.
The measured normalized drain current noise S_{ID}/I_D^2 (square symbols) is plotted together with the carrier-number-fluctuation (CNF) reference curve represented by constant × $(g_m/I_D)^2$ (circle symbols). Across the entire drain current range, the two trends show strong agreement, indicating that the low-frequency noise behavior is well described by CNF-dominated fluctuations. This consistency confirms that S_{ID}/I_D^2 scales proportionally with $(g_m/I_D)^2$ as expected for CNF-driven LFN mechanisms.

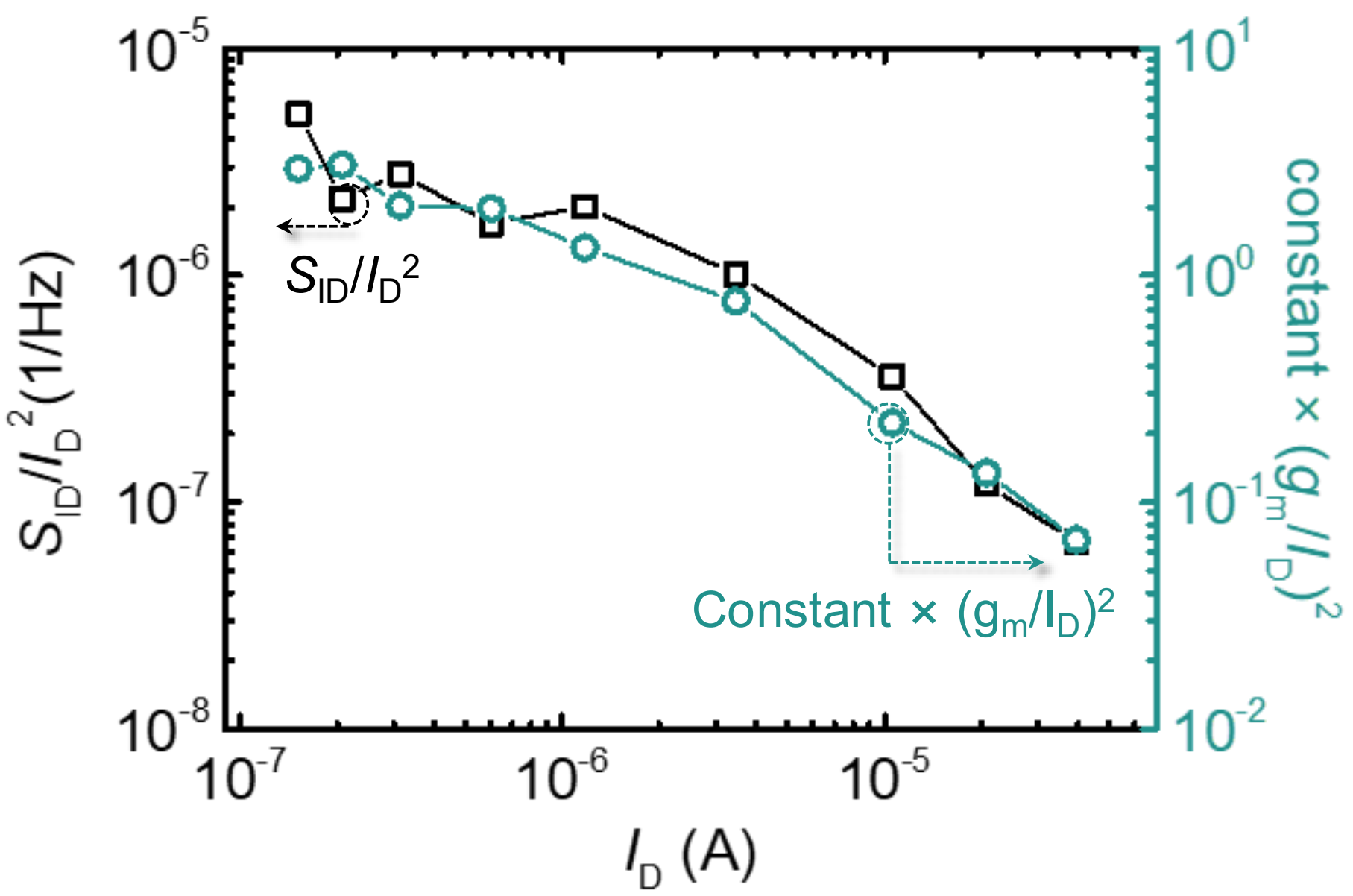

Supplementary Figure 13. Transient measurements under different gate biases

Transient drain current ($I_{D,t}$) traces measured at V_{GS} = 2 V **a**, 3.5 V **b**, and 4 V **c**. All measurements were performed using a Keysight B1500A semiconductor parameter analyzer equipped with the WGFMU module, with a sampling rate of 800 Hz. The time-domain fluctuations captured over a 20 s window provide the raw current data used for autocorrelation analyses.

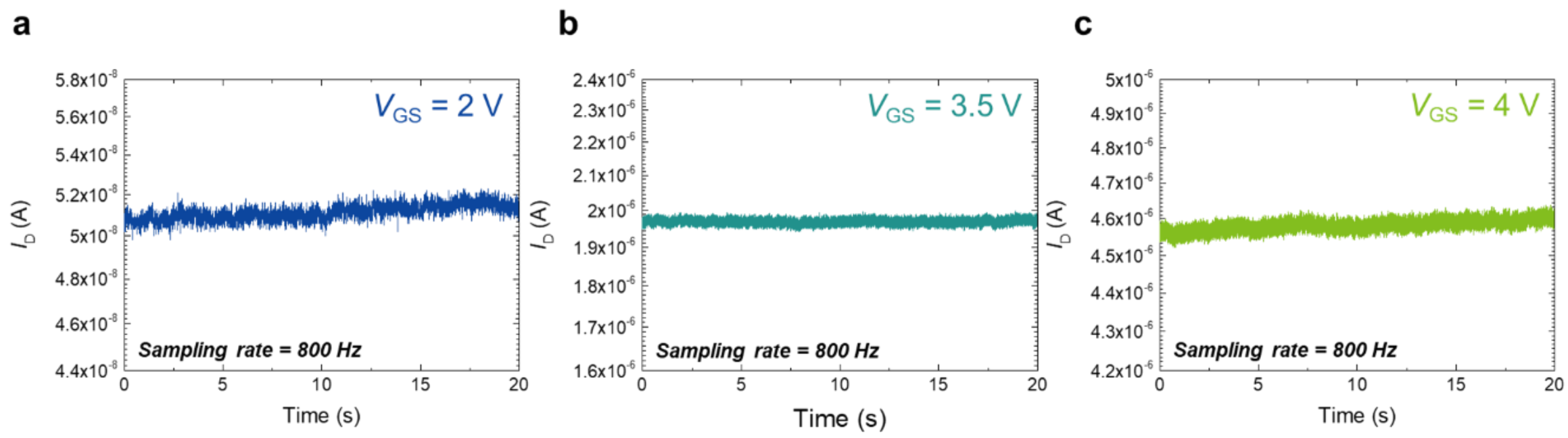

Supplementary Figure 14. Autocorrelation analysis of $I_{D,t}$ at different lag times

Scatter plots of $I_{D,t}$ versus $I_{D,t+\tau}$ for lag times $\tau = 1, 5$ measured at V_{GS} = 2 V, 3.5 V, and 4 V. These plots reveal the evolution of autocorrelation strength across different bias conditions, quantified by the Pearson coefficients shown in each panel.

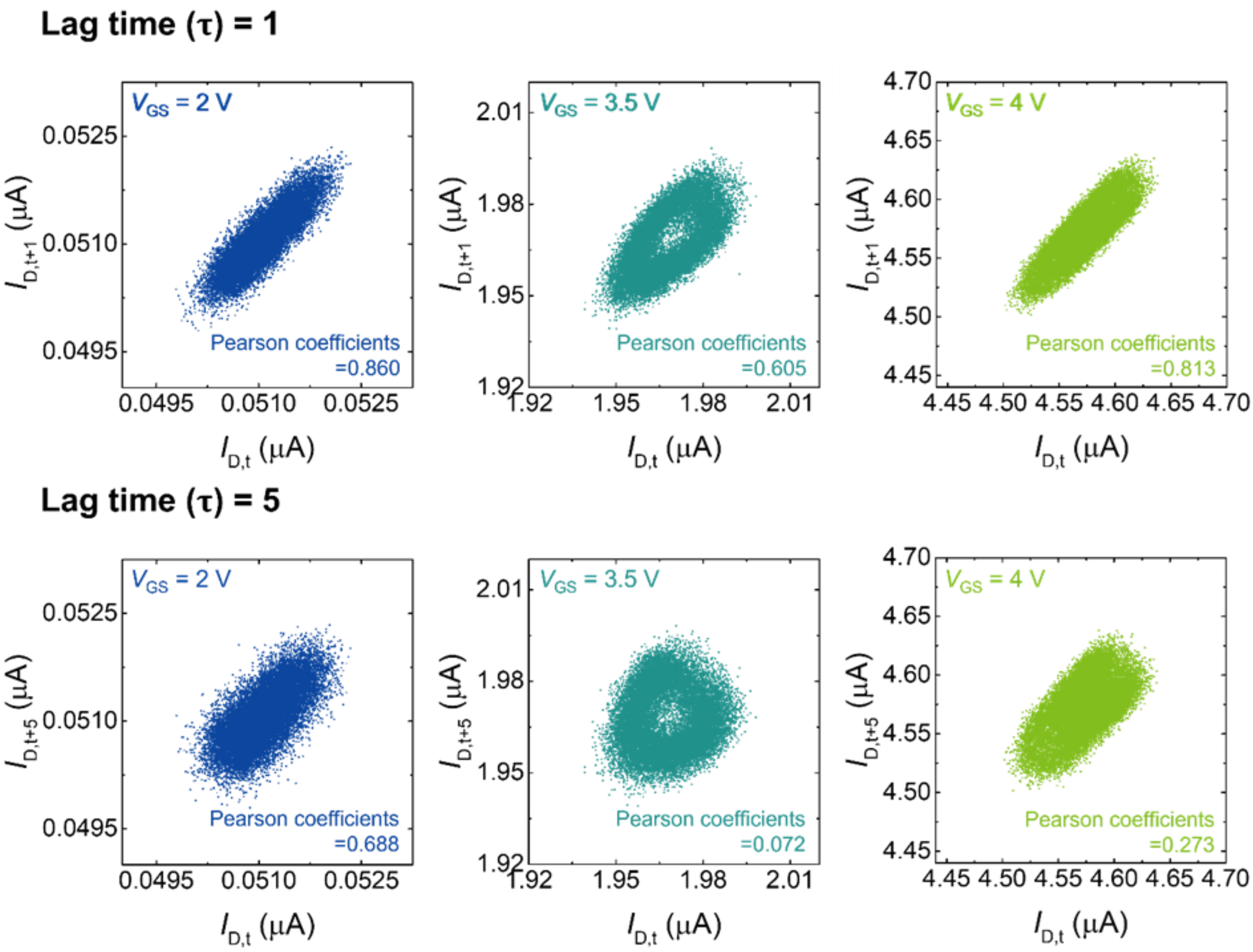

Supplementary Figure 15. Evolution of Pearson correlation coefficients with gate bias

Pearson correlation coefficients (ρ) extracted from ($I_{D,t}$, $I_{D,t+\tau}$) pairs for lag times τ = 1.25 and 6.25 ms as a function of V_{GS}. Across the full V_{GS} range, ρ remains relatively high, indicating strongly correlated defect-driven current fluctuations. A pronounced drop in ρ emerges near V_{GS} = 3.5–3.75 V for both lag conditions, which coincides with the bias region where trap-mediated screening collapses the memory window, weakening temporal correlation. At higher V_{GS}, the correlation increases again as the device enters the regime where V_O migration–induced ionic switching becomes dominant, restoring stronger temporal correlation in $I_{D,t}$.

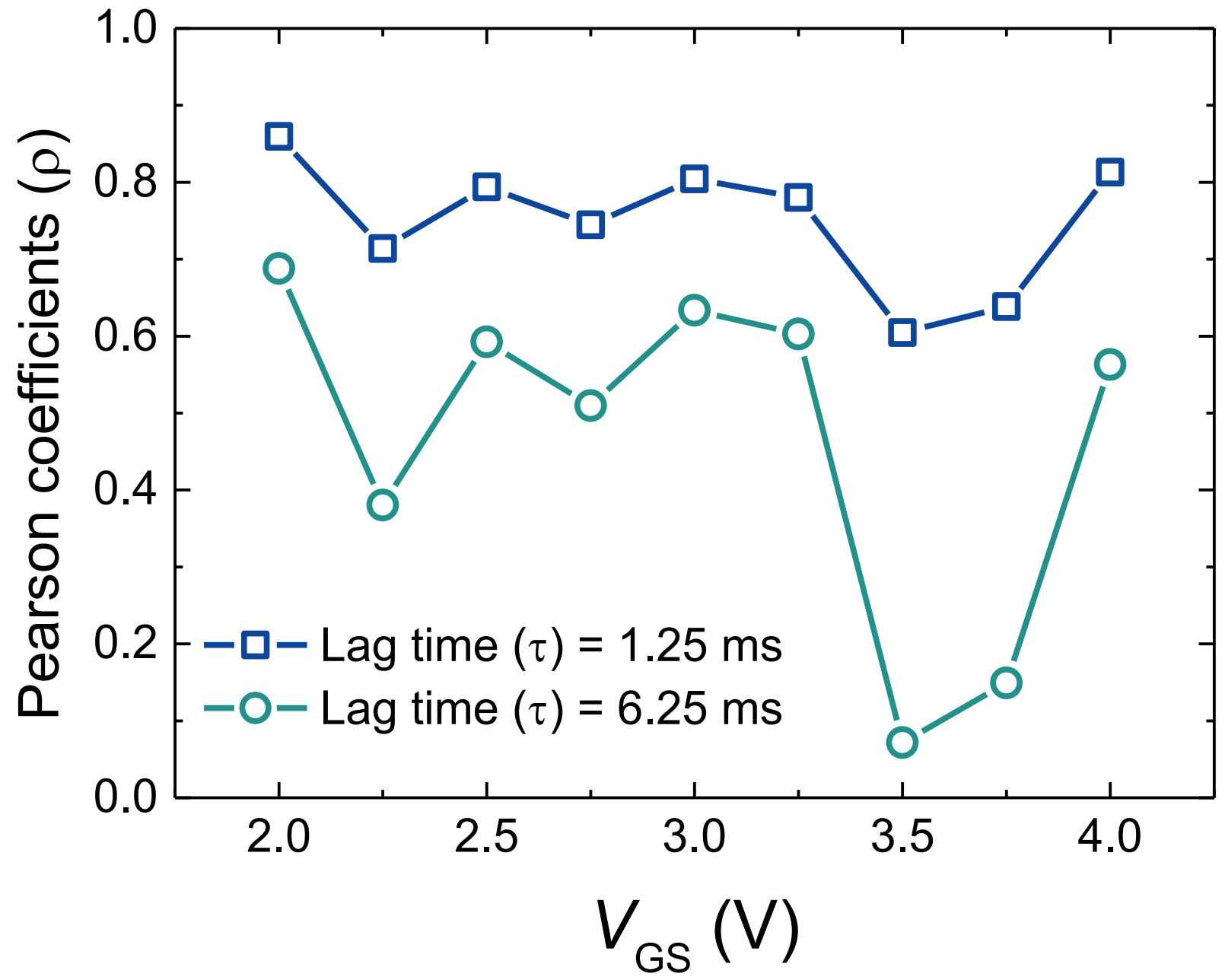

Supplementary Figure 16. Retention characteristics of the FITFET

Retention characteristics of the FITFET measured under erased, ferroelectric and hybrid ferro-ionic programmed states, showing stable characteristics from 10^0 to 10^4 seconds.

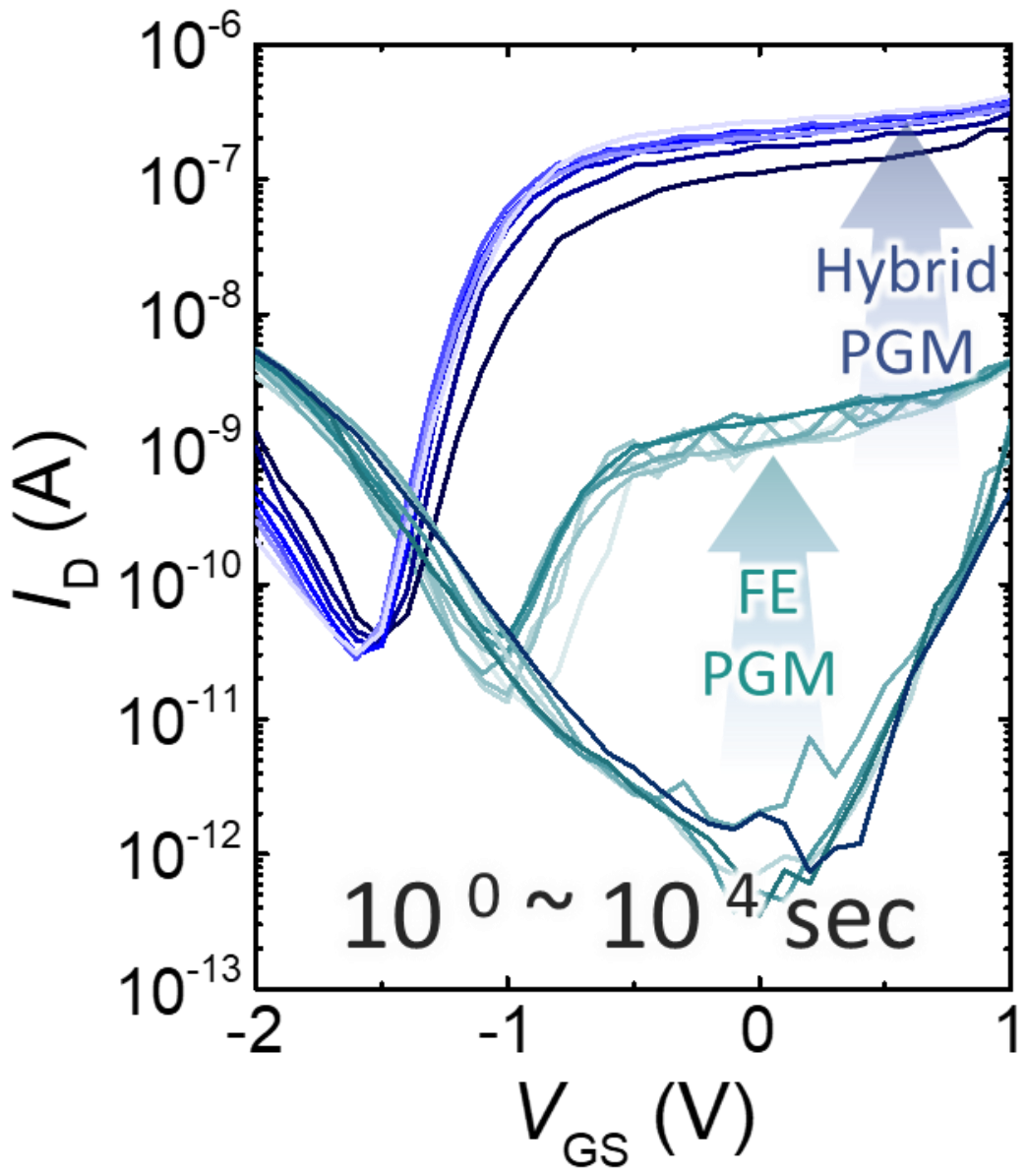

Supplementary Figure 17. Pulse bias dependent transition from ferroelectric to hybrid ferro and ionic switching

Transfer characteristics (I_D-V_{GS}s) of the FITFET measured under V_{PGM} voltages of **a** 3.0 V, **b** 4.0 V, **c** 5.0 V, **d** 6.0 V, and **e** 7.0 V. **f** Transfer characteristics measured over an extended read range, confirming the MW collapse is also observed under pulse-based operation at elevated V_{GS} conditions. The FITFET exhibits a distinct transition from ferroelectric-dominant switching at low V_{PGM}s to hybrid ferro-ionic switching at higher V_{PGM}s, indicating the progressive activation of ionic migration and the coexistence of dipole- and vacancy-driven conduction mechanisms.

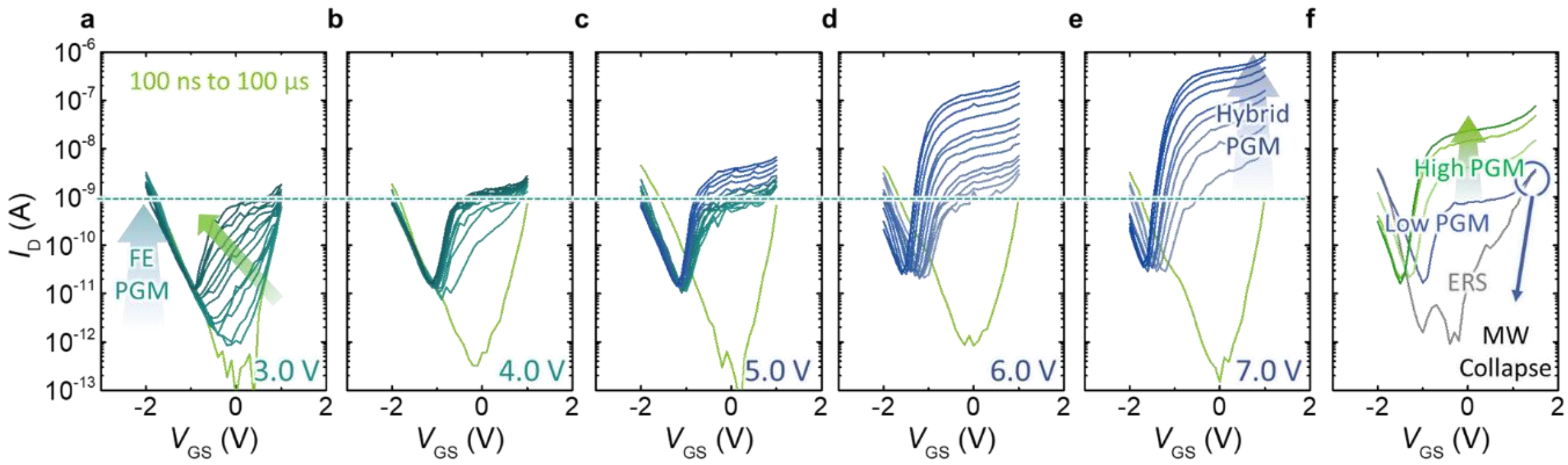

Supplementary Figure 18. Device to device uniformity of ferroelectric and hybrid ferro and ionic switching

Transfer characteristics ($I_{D,\ G}$-V_{GS}s) measured from 30 individual devices under **a, d** the FE-dominant, **b, e** trap-mediated and **c, f** hybrid ferro-ionic regimes, respectively. MW ratio measured at V_{GS} = 0 V under **g** FE-dominant, **h** trap-mediated, and **i** hybrid regime, showing consistent MW collapse at $V_{GS} \approx$ 1.18–1.21 V and re-opening of the MW beyond ~3.9 V across devices.

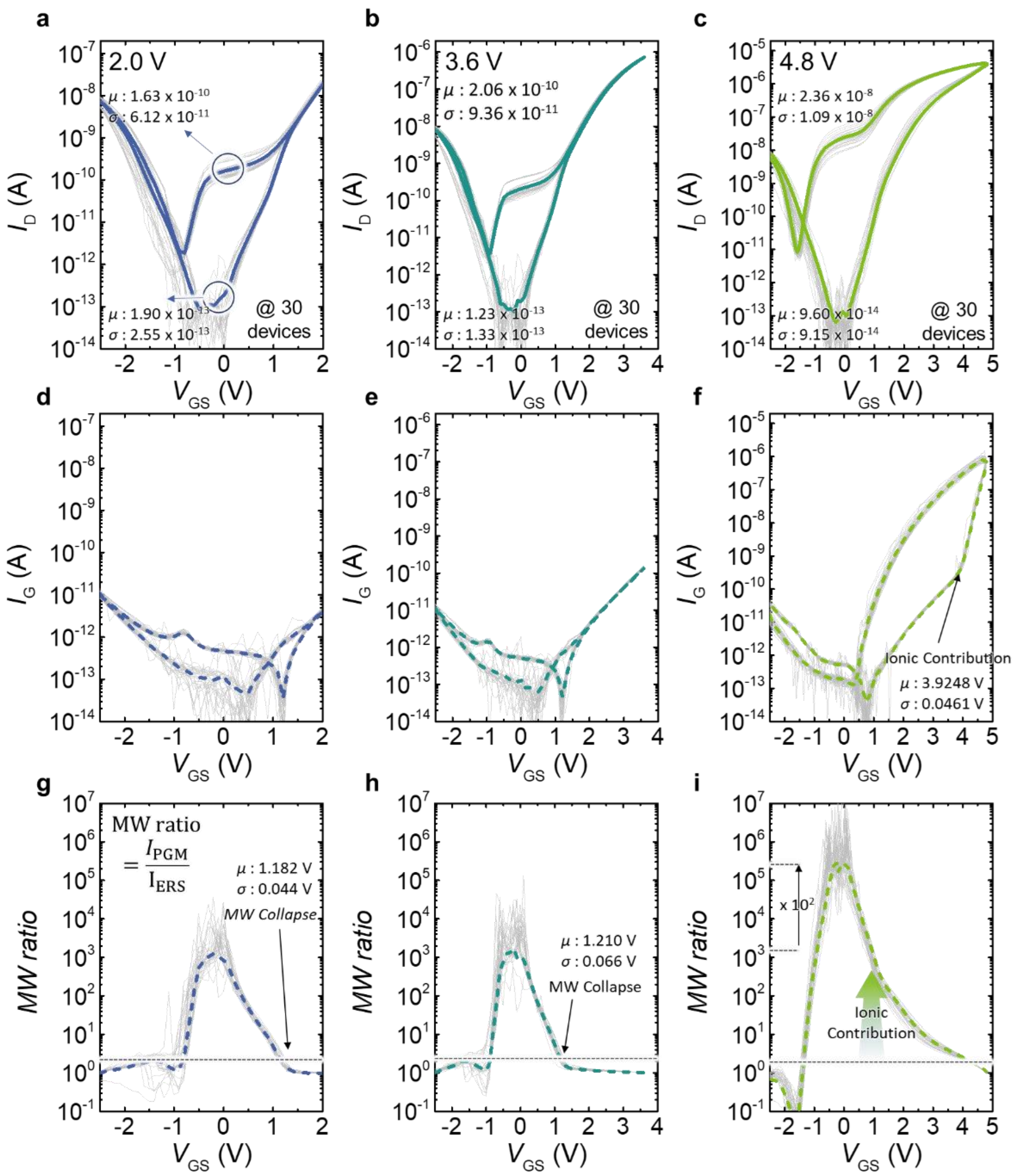

Supplementary Figure 19. Repeated mode toggling characteristics of the FITFET

a-c DC Transfer characteristics measured under repeated toggling between 2.0 V, 3.0 V, and 3.5 V. The bias sequence 2.0 V → 3.0 V → 3.5 V was consecutively applied and repeated 30 cycles. **d-f** Pulse based transfer characteristics measured under repeated toggling between Erase, FE-PGM, and Hybrid-PGM states. Unlike the FE-dominant and trap-mediated states, the hybrid-PGM state involves ionic contributions, making repetitive DC sweeping impractical. Therefore, AC pulse operation was used as the method to repeatedly toggle. The negligible variation among the transfer curves confirms stable and reversible mode transitions under repeated DC and AC modulation.

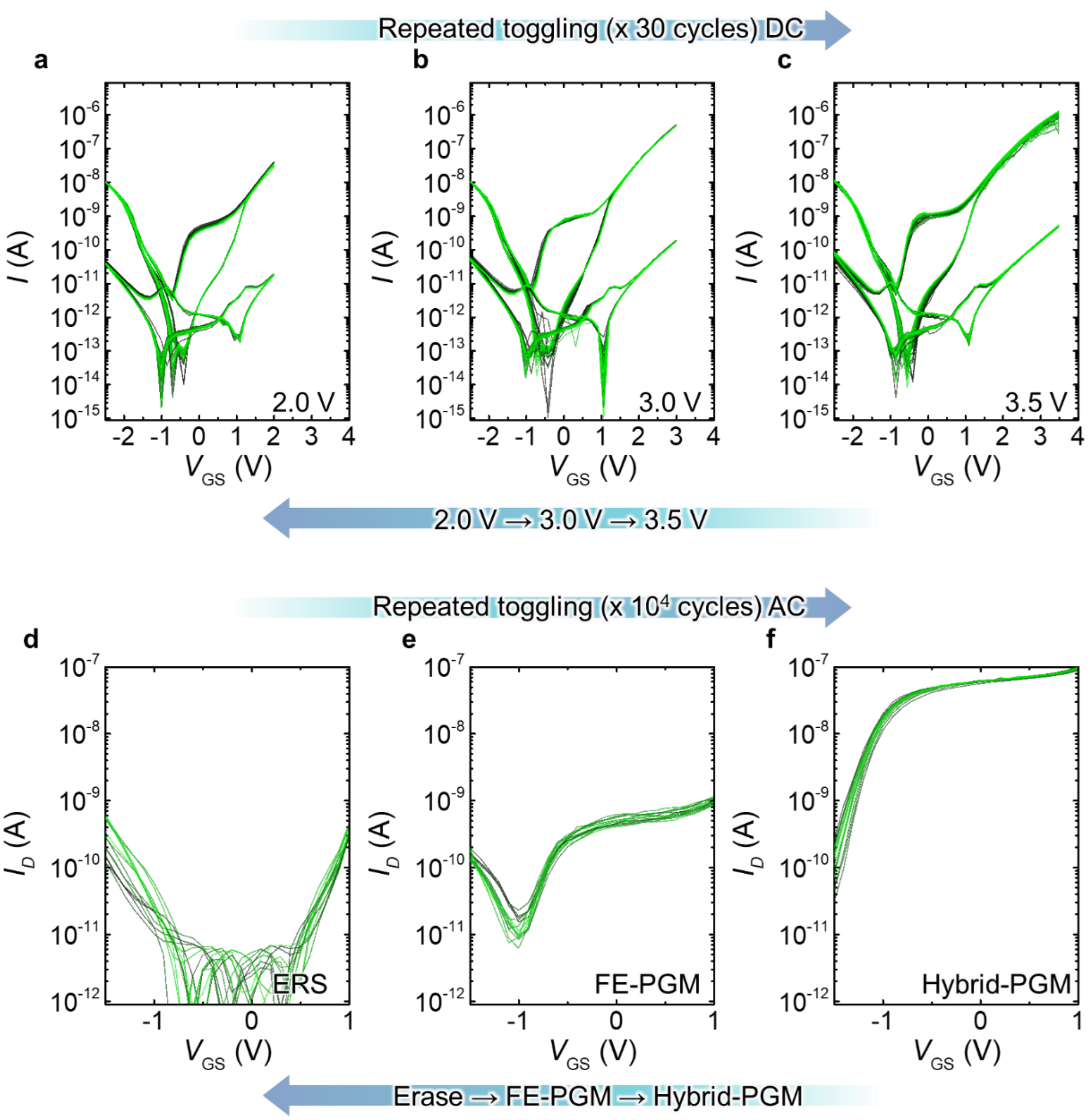

Supplementary Figure 20. Endurance characteristics under different programming

a I_D evolution as a function of the number of switching cycles for fully ERS (-4 V 10 μs), FE PGM (4V 1 μs), and Hybrid PGM (6V 10 μs), demonstrating stable states without noticeable degradation. Corresponding I_D-V_{GS} measured during endurance cycling for **b** fully ERS, **c** FE PGM, and **d** Hybrid PGM states.

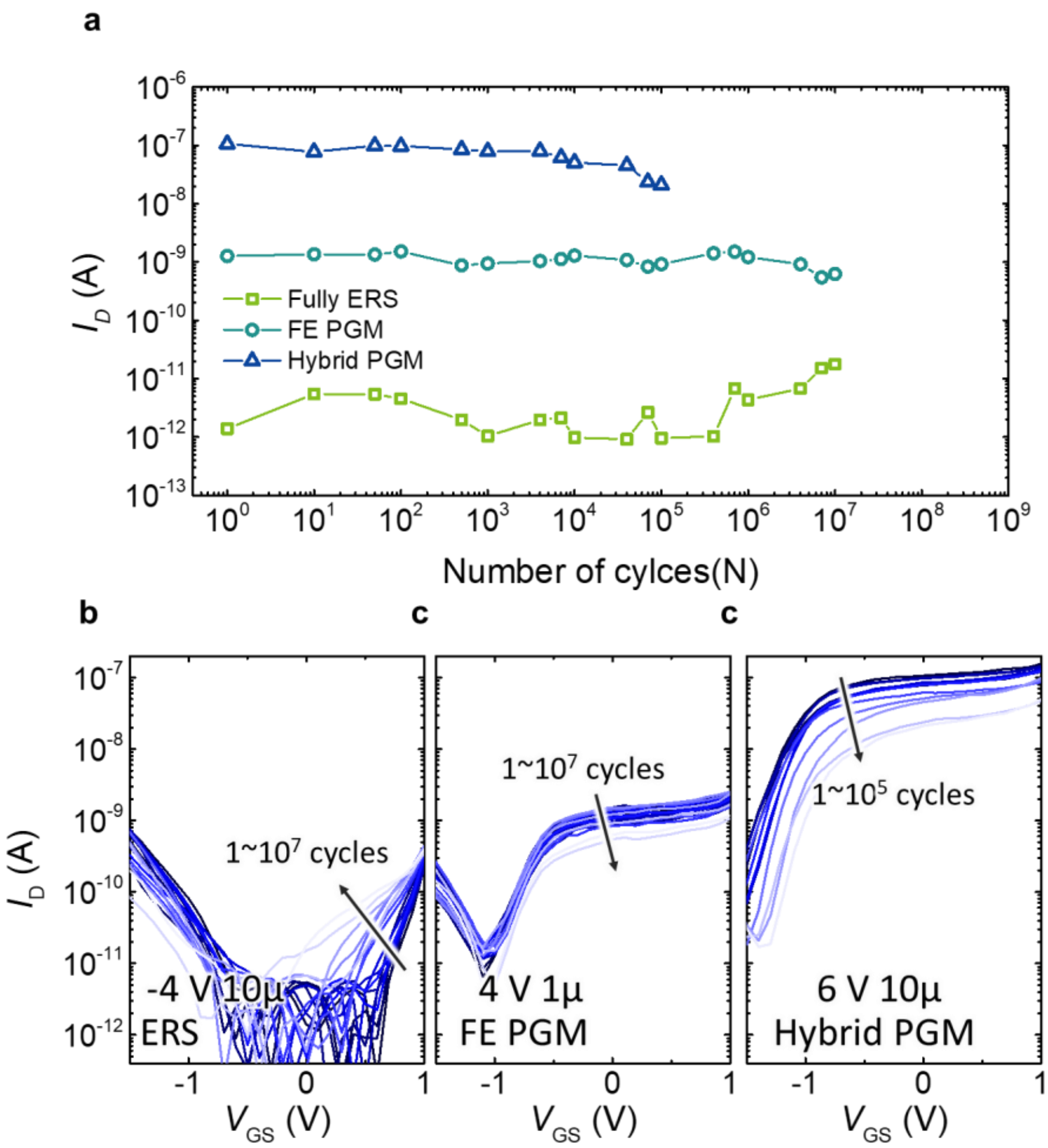

Supplementary Figure 21. Repeated secure mode stress test of the FITFET

Evolution of I_D as a function of pulse count under repeated application of secure mode pulses (1.25 V, 50 ns), showing negligible current variation (~1 pA) and stable operation up to 10^6 cycles without cumulative degradation or unintended programming.

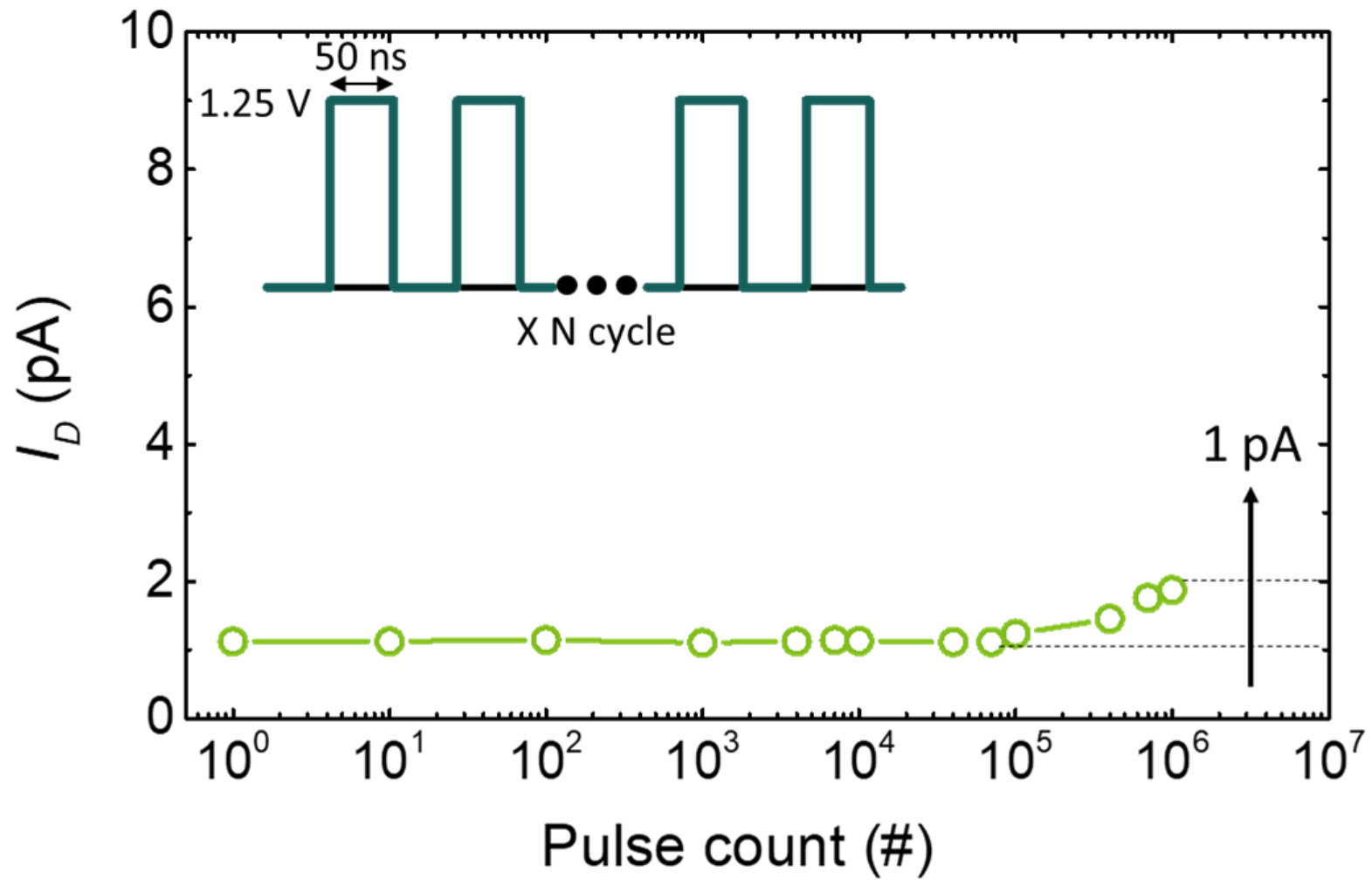

Supplementary Figure 22. Array level electrical characteristics and selective operation schematics of the AND type FITFET array

a DC I_D–V_{GS} double-sweep characteristics of all 288 cells, showing typical counterclockwise hysteresis. **b** I_D–V_{GS} of 120 randomly selected cells under PGM and ERS states. **c** Current distribution at V_{read} = 0.5 V extracted from **b**, highlighting clear separation between PGM and ERS states. **d** Multi-level potentiation and depression characteristics obtained by applying incremental pulse trains (2.6 V, ΔV = 0.04 V, 1 μs for potentiation; -2.6 V, ΔV = -0.045 V, 1 μs for depression; V_{read} = 0.1 V). **e** Selective PGM and **f** ERS operations showing the responses of target and inhibit cells. Only the target cell was successfully programmed or erased, while the unselected cells remained unchanged. **g** Schematic of the selective ERS condition. **h** Schematic illustration of the weight-transfer operation in the FITFET array.

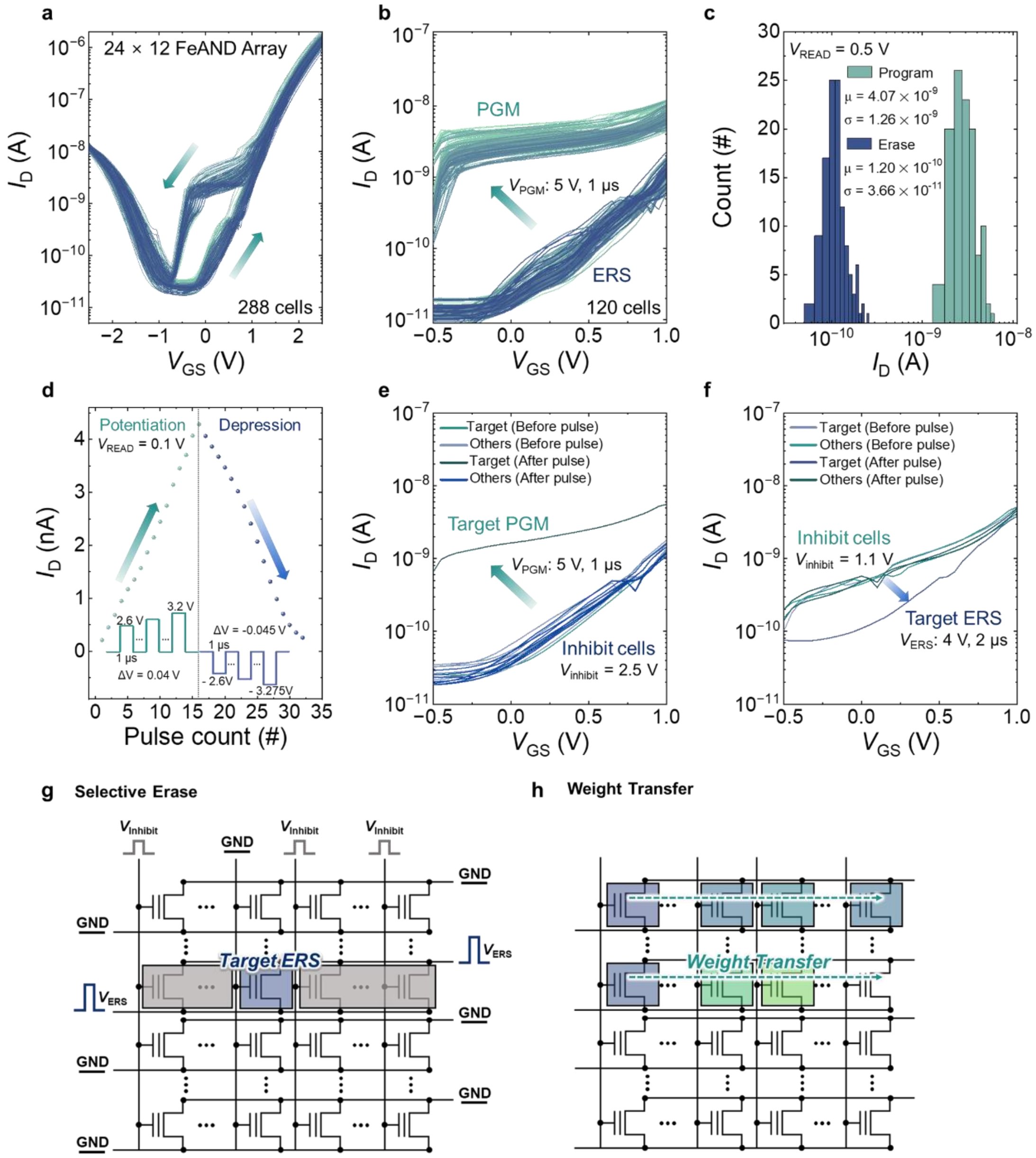

Supplementary Figure 23. Array level vector-matrix multiplication operation of the FITFET array

a Measured and calculated VMM currents from 8000 input patterns at V_{read} = 0.5 V. For each 24-bit input, a logic '1' turns the corresponding cell ON by applying 0.5 V, while a logic '0' applies an unselected bias of -0.5 V. **b** Schematic of the measured VMM current representing the total output current when multiple cells are turned ON simultaneously. **c** Schematic of the calculated VMM current showing the summed ON currents of individual cells under the same input pattern.

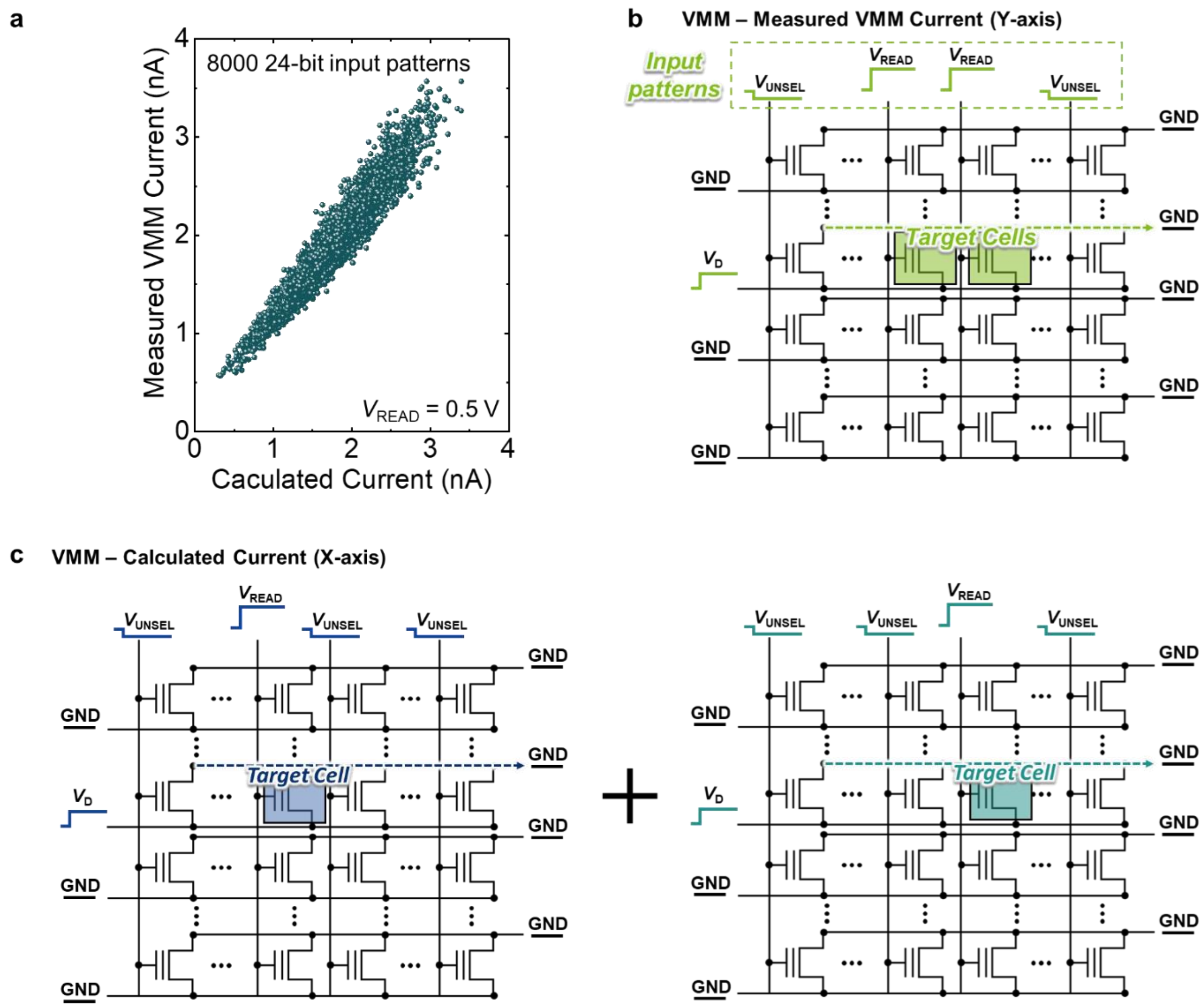

Supplementary Figure 24. Reversible conceal and reveal operation of the FITFET array
Reversible conceal–reveal behavior demonstrated using a different input image, showing image transfer at 0.5 V (plain mode), concealment at 1.5 V (secure mode), and full recovery upon returning to 0.5 V. The color scale represents conductance ranging from 0.4 to 1.8 nS.

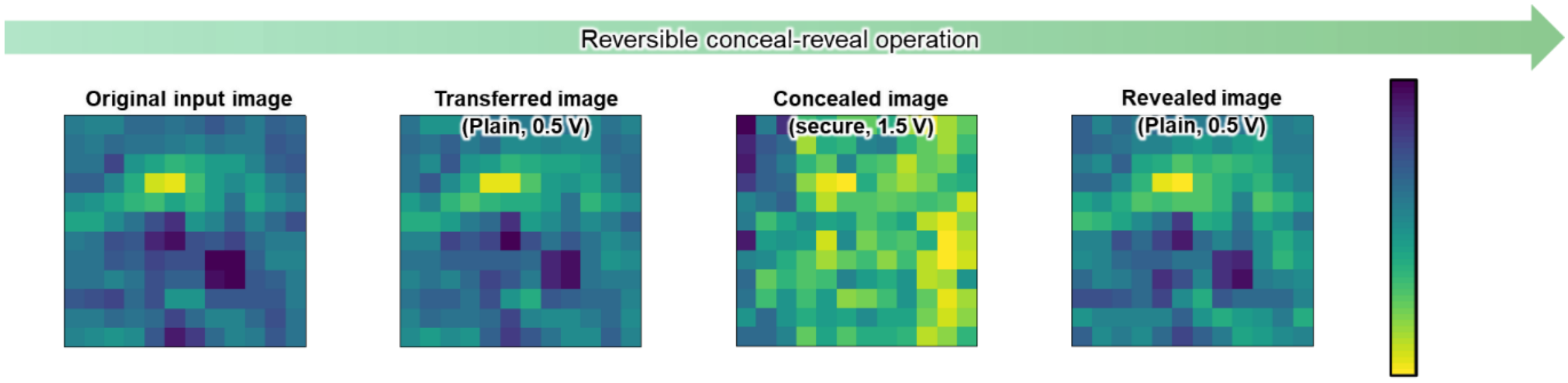

Supplementary Figure 25. Read disturb evaluation under array level read operation

a Schematic illustration showing different read-disturb contributions during the read operation in the FITFET array, including repeated secure-read bias on the selected WL and negative bias on unselected WLs. **b** Drain current (V_{READ} = 0.5 V) stability under repeated pulses (1µs) up to 10^6 cycles, where a secure-read voltage (1.5 V) was applied to the selected WL and a negative bias (V_{UNSEL}= -0.5 V) was applied to the unselected WLs. The results confirm that neither bias condition induces disturbance of the erased or programmed states.

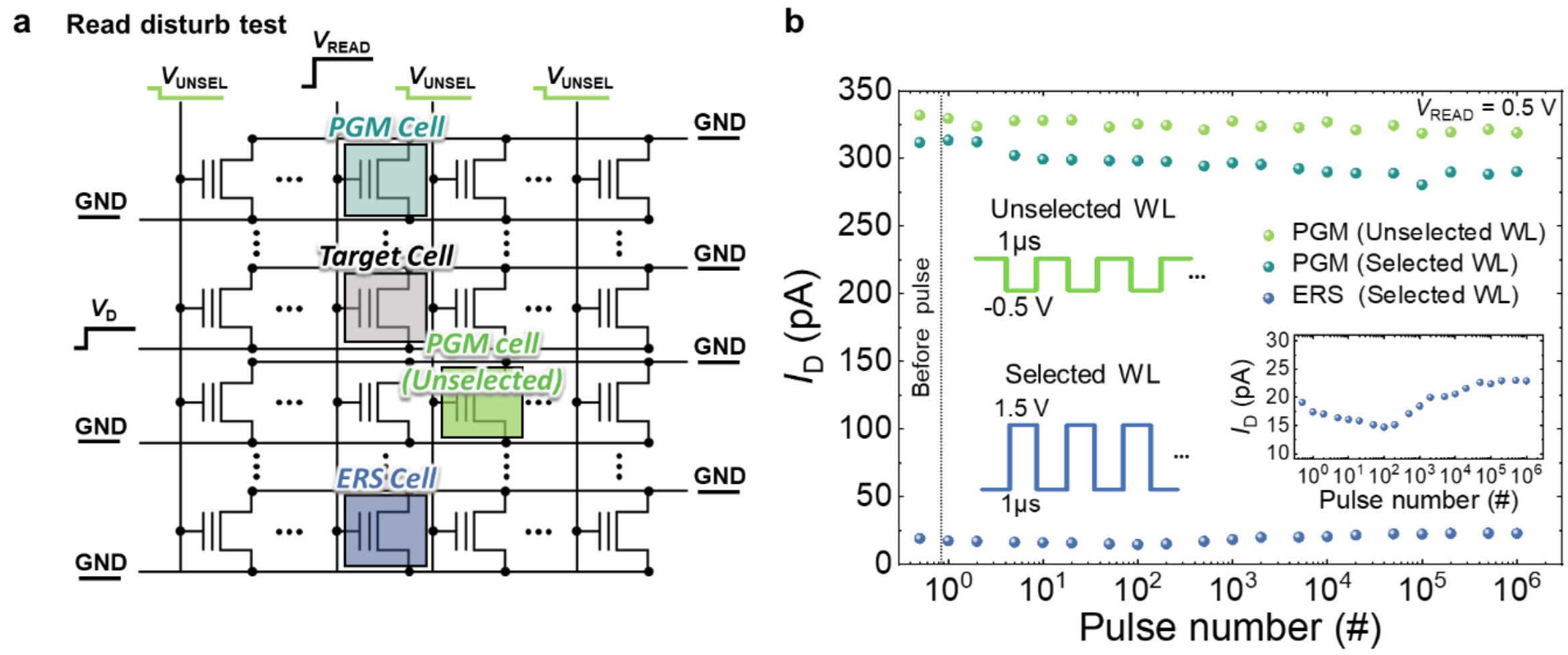

Supplementary Table 1. Bias conditions for selective operations in the FITFET array

Summary of WL, BL, and SL bias conditions used to selectively PGM and ERS the target cell while suppressing unintended state changes in neighboring cells.

Program	WL	BL&SL
Target cell	V_{PGM} (5 V, 1us)	0 V
Other cells	0 V	Inhibit (2.5 V)

Erase	WL	BL&SL
Target cell	0 V	V_{ERS} (4 V, 2us)
Other cells	Inhibit (1.1 V)	0 V

Supplementary Table 2. Benchmark of concealment oriented devices

Device Structure	Material system	Encryption source	Concealment mechanism	CMOS compatibility	Array size	Concealment Voltage	Security	Ref. No
2-terminal memristor	HfO_x/TaO_x	Oxygen vacancies	Filament formation	Good	64 x 128 array	2.2 V	Concealed BER > 30%	[51]
2-terminal memristor	HfO_x	Oxygen vacancies	Filament formation	Good	256 x 64 array	3.0 V	Concealed BER > 40.51%	[52]
2-terminal memristor	HfO_x	Oxygen vacancies	Filament formation	Good	4 x 4 array	-3.5 V	Inter-HD 50.6%	[53]
2-terminal memristor	$R\text{-}(MBA)_2PbI_{4(0.7)}Br_{4(0.3)}$	Halide vacancies	Filament formation	Poor	10 x 10 array	- 2.0 V	Inter-HD 42.3%	[54]
3D vertical RRAM	$Al{:}HfO_2/Ta_2O_5$	Oxygen vacancies	Filament formation	Good	4 x 4 x 3 3D array	12 V	Inter-HD 50.63%	[55]
3D vertical NAND Flash	ONO	BL doping depth	Weak-GIDL erase	Good	> 100 layers	> 15 V	Concealed BER 49.4%	[56]
3-terminal FET	$HfO_2/TiO_2/SiO_2$	Interface traps	Trap-modulated screening	Good	24 x 12 array	1.25 V	CosSim ≈ 0.0	This work

Supplementary Note 1. Ferroelectric platform and material selection of $Hf_xZr_{1-x}O_2$

Hafnium–Zirconium oxide (HZO) has been widely established as a cornerstone material for ferroelectric device engineering, combining strong polarization switching with exceptional compatibility to modern CMOS process flows. The mixed-oxide system of HfO_2 and ZrO_2 allows compositional tuning of polymorphic phases, including orthorhombic (ferroelectric), tetragonal (antiferroelectric), and monoclinic (non-polar) phases. By adjusting the Hf/Zr ratio, the balance among these competing phases can be precisely modulated, enabling optimization of coercive voltage, remnant polarization, and wake-up behavior across diverse device configurations[1,2].

One of the most distinctive advantages of HZO is its robust ferroelectricity at nanometer and even sub-nanometer thicknesses, a regime where conventional perovskite ferroelectrics lose switchable polarization[3,4]. This scalability makes HZO uniquely suitable for ultra-scaled transistors, tunnel devices, and 3D-integrated memory arrays. In addition, its relatively low crystallization temperatures (400 – 600 °C) ensure full compatibility with backend-of-line (BEOL) and monolithic 3D integration schemes without degrading underlying layers[5,6].

From a processing perspective, HZO films can be conformally deposited by atomic layer deposition (ALD) with precise control over stoichiometry and thickness uniformity. The ALD process enables wafer-scale uniformity and intimate interface formation with dielectrics such as SiO_2 and TiO_2, both of which play key roles in modulating oxygen vacancy (V_O) concentration and interfacial dipole coupling.

Collectively, these features—scalability, CMOS integrability, and controllable switching dynamics—make HZO an ideal base material for realizing multifunctional transistors that merge nonvolatile memory, analog modulation, and hardware-level encryption capabilities.

Supplementary Note 2. STEM and EELS characterization of V_O distribution in HZO

The spatial distribution of oxygen within the ferroelectric stack was examined using scanning transmission electron microscopy coupled with electron energy-loss spectroscopy (STEM-EELS). Two-dimensional spectrum images were collected to visualize elemental variations across the SiO_2, TiO_2, and HZO layers. The measurements were performed with an energy dispersion of 0.25 eV per channel to ensure sufficient spectral resolution for quantitative analysis. Both low-loss (-29.85 eV to 256.95 eV) and core-loss (370.15 eV to 656.95 eV) regions were recorded to capture the relevant energy-loss edges.

Quantitative oxygen mapping was carried out using Gatan Microscopy Suite 3 (GMS 3), following an analytical procedure based on the methodology proposed by Verbeeck and Van Aert[7]. A power-law background subtraction was applied prior to integration of the O K-edge, and Hartree-Slater ionization cross-section modeling was used to estimate oxygen content. To enhance the accuracy of the fitting, the first 40 eV of the energy loss near-edge structure (ELNES) region was excluded, and the fitting was performed over the 500.3 – 657 eV range. The resulting oxygen concentration profiles provide spatially resolved information on stoichiometry variation and interfacial oxygen deficiency across the stack.

In addition to compositional quantification, the O K-edge ELNES fine structure was analyzed to probe the local electronic environment associated with V_Os. The O K-edge spectrum typically exhibits two characteristic peaks, labeled p_1 (~534.9 eV) and p_2 (~538.38 eV), corresponding to electronic transitions from O 1s core states into unoccupied hybridized Hf/Zr-O conduction band states. The relative intensities of these peaks are highly sensitive to local bonding configurations and V_O concentration. Previous theoretical EELS works incorporating core-hole effects have shown that the p_1 component weakens in oxygen-deficient regions due to a reduction in unoccupied O 2*p*-Hf/Zr *d* hybrid states[8,9]. In this work, the p_1/p_2 intensity ratio of the O K-edge is employed as a spatial indicator to identify the degree and distribution of V_O across the HZO layer, providing direct insight into the correlation between interfacial defect density and local electronic structure (Supplementary Fig. 6). It should be noted that the p_1/p_2 intensity ratio reflects relative trends in V_O concentration rather than an absolute quantitative measure and is therefore used here as a comparative indicator of spatial variation.

Supplementary Note 3. XPS depth profile analysis of interfacial V_O modulation in HZO

To evaluate the chemical states and depth-dependent oxygen stoichiometry in the fabricated transistors, X-ray photoelectron spectroscopy (XPS) was performed using a Thermo Fisher Scientific NEXSA system. All spectra were calibrated to the C 1s reference peak at 284.8 eV. The O 1s core-level spectra were deconvoluted into two Gaussian components following established fitting procedures reported for hafnia-based oxides[10,11]. The lower binding energy peak represents oxygen in Hf-O or Zr-O coordination, whereas the higher-binding-energy component corresponds to oxygen-deficient or non-lattice oxygen species, which are commonly associated with V_Os.

Depth-profile analyses were conducted to trace the variation of V_O concentration through sequential etching across the SiO_2–TiO_2–HZO gate stacks. As shown in Supplementary Fig. 7, the overall V_O ratio in TiO_2–interfaced devices (FIDFET and FITFET) are higher than that of the FeFET (without TiO_2).

Consequently, the engineered interface produces a graded V_O profile—higher near the TiO_2 interface—demonstrating controlled modulation of V_O density through interlayer and dielectric thickness design. This tailored defect distribution underpins the observed ferro-ionic coupling and bias-dependent switching dynamics in the TiO_2-engineered devices.

Supplementary Note 4. Comparative switching dynamics of transistors

To evaluate the effect of interface engineering on the switching mechanisms and properties, the structural and electrical characteristics of FeFET, FIDFET, FITFET were comparatively investigated (Supplementary Fig. 10).

FeFET (SiO_2–HZO) shows a stable MW that remains nearly unchanged with V_{GS}, signifying pure ferroelectric switching dominated by dipole polarization. However, when V_{GS} exceeds a certain threshold, interfacial traps begin to form, which deteriorate the MW through charge trapping and screening effects—an indication of the inherent limitation of conventional FeFETs (Supplementary Fig. 8).

FIDFET (SiO_2–TiO_2–HZO) introduces an oxygen-scavenging TiO_2 interlayer, producing hybrid ferro-ionic coupling between ferroelectric polarization and ionic migration. This coupling allows a gradual expansion of the MW and a bias-dependent rise in I_G, indicating that field-driven migration of V_O contributes to the additional switching.

FITFET (SiO_2–TiO_2–HZO, thinned SiO_2 interlayer) further refines the interfacial state through SiO_2 thinning and TiO_2 coupling. The device exhibits three distinct regimes:

Region I: ferroelectric polarization-dominated switching at low V_{GS} (1–2 V).

Region II: temporary MW collapse in the intermediate range (2–4 V) due to interfacial traps.

Region III: MW reopening at high V_{GS} (4–5 V) induced by ionic migration.

These transitions are supported by the corresponding I_G behavior, where a polarization peak at low V_{GS} is followed by a continuous ionic-current increase at high V_{GS}.

Collectively, these observations demonstrate an open–collapse–reopen MW transition unique to the FITFET, confirming that interface-engineered dual modulation of SiO_2–TiO_2 layers enables both ferroelectric and ionic functionalities. The systematic transition from FeFET → FIDFET → FITFET represents an evolution from pure ferroelectric to hybrid ferro-ionic and finally dual-mode operation, achieving enhanced tunability and intrinsic hardware-level encryption capability through bias-controlled concealment of ferroelectric and ionic states.

Supplementary Note 5. Carrier number fluctuation-based depth profiling

Within the carrier number fluctuation (CNF) model, fluctuations in the trapped charge density modulate the flat-band voltage, which in turn manifests as low-frequency drain-current noise. The drain current normalized noise spectral density (S_{ID}/I_D^2) is expressed as

$$S_{\mathrm{ID}}/{I_D}^2 = \frac{q^2kT}{C_{ox}^2}\frac{N_t}{f}\frac{{g_m}^2}{{I_D}^2} \quad (1),$$

where q is the elementary charge, k is the Boltzmann constant, C_{ox} is the gate oxide capacitance per unit area, g_m is the transconductance of FET, and N_t is the trap density per unit energy and area. Hence, S_{ID}/I_D^2 is proportional to $(g_m/I_D)^2$, which shows the experimental correlation with this proportionality (Supplementary Fig. 11). Assuming the characteristic time constant of traps depends exponentially on their depth as, the trap depth can be mapped to frequency through:

$$z = \lambda \ln\left(\frac{1}{2\pi f \tau_0}\right) \quad (2)$$

where z is the vertical distance from Si/SiO_2 interface, τ_0 is the time for tunneling into a trap state at the interface ($z = 0$), and λ is the tunneling attenuation factor. This relationship enables the transformation of the measured frequency-dependent spectra into a spatially resolved trap density profile, thereby offering insight into the depth-dependent distribution of active defects depending on the operating regions.

Supplementary Note 6. Array level measurement: vector-matrix multiplication

To verify the vector–matrix multiplication (VMM) capability of the 24 × 12 AND-type FITFET array, the ON-state current of every cell was first extracted by individually turning each cell ON at V_{read} = 0.5 V with V_{DS} = 0.1 V. These individually measured ON-currents served as reference values for subsequent comparison. Next, 8000 random 24-bit input patterns consisting of '0's and '1's were generated. For each pattern, a logic '1' turned the corresponding word line (WL) ON by applying 0.5 V, whereas a logic '0' applied an unselected bias of –0.5 V. Under these conditions (V_{DS} = 0.1 V), the resulting output current represented the measured VMM current (Y-axis), corresponding to the total current flowing when multiple cells were simultaneously turned ON. For the calculated current (X-axis), the ON-state currents of only those cells corresponding to logic '1' were summed using the individually measured reference currents. As shown in Supplementary Fig. 23, the measured currents cluster closely along the $y=x$ line across all 8000 input patterns, demonstrating that the array-level output is highly consistent with the summed individual cell currents. This agreement confirms that reliable VMM operation is achieved in the FITFET-based AND-type memory array.

Supplementary Note 7. Array level measurement for selective program, erase, and read operation

To access only the desired cells within the array, appropriate bias conditions were applied to the word lines (WLs), bit lines (BLs), and source lines (SLs) during each operation. During the selective program operation, a program pulse (5 V, 1 μs) was applied to the WL of the target cell, while an inhibit bias (2.5 V) was applied to the BLs and SLs of unselected cells sharing the same WL to suppress unintended programming. For the read operation, V_{read} was applied to the WL of the target cell, and V_{DS} = 0.1 V was applied to the corresponding BL. A small negative bias (-0.5 V) was applied to unselected WLs to minimize residual current contributions from other cells sharing the same BL, ensuring that the measured current primarily reflects the target cell. In the selective erase operation, a positive pulse (4 V, 2 μs) was applied to the BL and SL of the target cell, while an inhibit bias (1.1 V) was applied to the WLs of unselected cells to prevent unwanted erasing. The inhibit bias was optimized to ensure that it did not induce inadvertent programming in neighboring cells. Additionally, the selective erase operation can also be performed, similar to the selective program case, by applying a negative pulse to the WL of the target cell while applying a negative inhibit bias to the unselected BLs and SLs.

Supplementary Note 8. Array level measurement of weight transfer

For validating voltage-controlled image concealment, weight transfer was performed on the 24 × 12 AND-type FITFET array. Before initiating the transfer process, all cells were fully erased to establish uniform initial states, which was accomplished by applying a -3 to 3 V double sweep across the entire array. The input image was normalized using the current levels corresponding to the fully erased and fully programmed states of the devices, enabling a consistent conversion from pixel intensity to target conductance. Weight programming was conducted sequentially along the BL direction (Supplementary Fig. 24). For each pixel, if the sampled current at V_{read} = 0.5 V was lower than the target current assigned to a given pixel, a program operation (2.3–5 V, ΔV = 0.05 V, 1 μs) was applied; conversely, if the sampled current exceeded the target level, an erase operation (-2.5 to -5 V, ΔV = -0.05 V, 1 μs) was performed. This iterative tuning procedure continued until the deviation between the sampled and target currents at 0.5 V converged within 10%, ensuring accurate reproduction of the intended weight map across the array.

Supplementary Note 9. Methodology for energy benchmarking of concealment-oriented security devices in Supplementary Table 2

The prior works compared in Supplementary Table 2 address heterogeneous device roles, including security-only PUFs, unified PUF/TRNG chips, in-memory encryption engines, and optoelectronic synapse/PUF hybrids. Therefore, a single universal energy number is not physically meaningful. In particular, the proposed FITFET differs fundamentally from most prior works in that synaptic functionality and hardware-level concealment are co-implemented in the same transistor, whereas many previous devices realize security through a dedicated PUF primitive or through an explicit conceal/recover programming sequence, which is separated from synaptic devices. Therefore, the benchmark was organized using two complementary descriptors: synaptic/compute functionality, and additional security-function energy overhead. For PUF-only devices, the synaptic term is not applicable. For each literature entry, we either adopted the energy value directly reported in the primary paper or conservatively reconstructed the governing energy expression from explicitly disclosed operating conditions. When essential parameters such as pulse width, transient current, or active illuminated area were not quantitatively mentioned, we intentionally avoided back-calculating an absolute energy number and reported only the corresponding governing expression.

In general, the operation energy is defined by

$$E = \int_0^{t_{\mathrm{op}}} V(t)I(t)\, dt. \ (3)$$

For a resistive read operation under quasi-static bias, this reduces to

$$E_{\mathrm{read}} \approx VIt = \frac{V^2}{R}t = GV^2t, (4)$$

where R and G denote effective resistance and conductance, respectively. For a multi-element circuit, the total energy can be written as

$$E_{\mathrm{tot}} = \sum_k \int \quad V_k(t)I_k(t)\, dt. (5)$$

If the original paper reports only a power trace, the energetically faithful quantity is

$$E = \int P(t)\, dt. (6)$$

For optoelectronic devices, the total operation energy is more appropriately separated into electrical and optical contributions

$$E_{\mathrm{tot}} = E_{\mathrm{elec}} + E_{\mathrm{opt}}, (7)$$

with

$$E_{\mathrm{elec}} = \int V(t)I(t)\, dt, and\ E_{\mathrm{opt}} = \mathcal{I}_{\mathrm{opt}}At, (8)$$

where $\mathcal{J}_{\mathrm{opt}}$ is the optical intensity and A is the illuminated area. Unless otherwise stated in the original paper, device-level estimates exclude peripheral circuits, line charging, and controller overhead.

This work: FITFET

For the proposed FITFET, the security function is realized by read-bias selection in the same synaptic transistor, rather than by a separate PUF block or by an explicit conceal/recover write sequence. Accordingly, the incremental security-step energy overhead at the device-architecture level is effectively negligible, because no additional SET/RESET-style concealment step is required to enter the secure state. Nevertheless, to provide a device-level number, we estimated the selected-cell drain-side secure-read energy using the experimentally used array-level read condition and the conductance range observed during the conceal–reveal operation. The corresponding expression is

$$E = \int_0^{t_r} V_{DS}\, I_D(t)\, dt \approx G_{\mathrm{sec}} V_{DS}^2 t_r. \;(9)$$

Using V_{DS} = 0.1 V and t_{r} = 1 μs, and G = 0.45 μS, the selected-cell secure-read energy is estimated to be 4.5 fJ. This estimate is intentionally restricted to the selected-cell, drain-side read path and does not include array-size-dependent peripheral contributions such as WL/BL line charging, decoder overhead, or the bias applied to unselected cells. This separation is important because the key architectural advantage of the FITFET lies not in a dedicated security-writing event, but in the fact that the secure state is accessed without a separate security-only operation.

Gao et al. (ref. 12)

Gao et al. reported a concealable memristive PUF in which readout mode and secure mode are achieved by a one-pulse RESET and SET operation, respectively. The paper explicitly presents the power track during the recovery and concealing of a 128-bit ID and the distribution of power consumption for one PUF bit, while also stating that only the power consumed in the memristor array is considered. Therefore, the physically faithful energy expressions for this work are

$$E_{\mathrm{Gao,128\text{-}}bit} = \int P_{\mathrm{array}}(t)\, dt \;(10)$$

and

$$E_{\mathrm{Gao,}bit} = \frac{1}{128} \int P_{\mathrm{array}}(t)\, dt. \;(11)$$

Because the main text does not tabulate the full numerical time-axis data for the power traces, we did not back-calculate a single absolute energy number for Supplementary Table 2. Instead,

we retained the operation principle that the security function requires an explicit SET/RESET-based conceal/recover step.

Li et al. (ref. 13)

Li et al. demonstrated a unified 28-nm 2T2R memristor-based PUF/TRNG chip with a concealable function. The paper explicitly reports that the PUF/TRNG read operation uses V_{read} = 0.2 V and that valid outputs are obtained within 100 ns, while chip-level PUF throughput reaches 67.6 Mbps. It also reports that concealment and recovery are implemented by SET/RESET operations over the entire array, with V_{set} = 2.7 V and V_{reset} = 3.0 V both using 4 μs pulses. On this basis, the pair-read energy of one 2T2R entropy cell can be written as

$$E_{\mathrm{Li,read}} = \int_0^{t_r} V_{\mathrm{READ}}\, I_{\mathrm{pair}}(t)\, dt \approx \frac{V_{\mathrm{READ}}^2}{R_{\mathrm{top}} + R_{\mathrm{bot}}} t_r, (12)$$

where R_{top} and R_{bot} are the instantaneous read-state resistances of the two memristors that define the PUF bit. The explicit security-step overhead is

$$E_{\mathrm{Li,conceal}} = \int_0^{4\mu s} V_{\mathrm{SET}}\, I_{\mathrm{SET}}(t)\, dt, (13)$$

$$E_{\mathrm{Li,recover}} = \int_0^{4\mu s} V_{\mathrm{RESET}}\, I_{\mathrm{RESET}}(t)\, dt. (14)$$

Because the dynamic switching current traces and machine-readable read-state resistance values are not numerically tabulated in the main text, we did not force a single absolute number for the conceal/recover energy. Nevertheless, this paper clearly differs from the FITFET in that concealment incurs a nonzero additional security-operation overhead, since the entire array must be explicitly SET/RESET to conceal and recover the PUF state.

Woo et al. (ref. 14)

Woo et al. reported task-level energies directly for a memristor-based platform combining unclonable key generation, logic, and encryption/decryption. The paper already tabulates total energy and latency for each task. The reported values are 99.9 pJ for encryption/decryption, and 134.3 pJ for SUM. Since the work does not operate as an analog synapse with bias-programmable secure readout, it was classified separately from the FITFET in the monolithic synapse/security descriptor

Kim et al. (ref. 15)

Kim et al. presented a chiroptical synaptic perovskite memristor that functions as a reconfigurable PUF, thus representing the closest prior example of a monolithic

synapse/security concept. However, this platform is fundamentally optoelectronic, and its reconfiguration relies on a simultaneous electrical and polarized-light stimulus. The paper reports that the 10 × 10 CSM array was reset by a 2.0 V electrical sweep and programmed to HRS using a -1.0 V pulse for 0.5 s under a selected polarized-light condition at a constant optical intensity of 100 μW/cm^2. Therefore, the appropriate total reconfiguration energy is 50 μJ/cm^2. However, because the active illuminated area and the full electrical transient during the 0.5-s programming event are not numerically tabulated, we did not extract a per-device or per-bit total energy.

Cho et al. (ref. 16)

Cho et al. introduced a vertical self-rectifying memristor platform in which PUF key generation and XOR-based in-memory encryption are realized in the same V-RRAM structure. Importantly, this study primarily benchmarked spatiotemporal cost, rather than providing a directly tabulated electrical energy value. The paper states that key generation is performed through a voltage-divider stage using a series resistance. The voltage applied across the (i,j)-th device may therefore be written in the standard voltage-divider form as

$$V_{A,i,j} = V_{G,i}\frac{R_{i,j}}{R_s + R_{i,j}}, (15)$$

where $V_{G,i}$ is the generation voltage applied to the i-th WL and $R_{i,j}$ is the resistance of the selected cell. During XOR operation, the paper reports a NIMP voltage V_{NIMP} = 5.0 V, a read voltage of 6 V, and a compliance current of I_{CC} = 50 nA. For the anti-serial NIMP pair, the voltage across the reverse-biased memristor follows the voltage-divider relation

$$V_{M1} = V_{\mathrm{NIMP}}\frac{R_{M1}}{R_{M1} + R_{M2}}. (16)$$

Accordingly, the NIMP energy may be bounded by

$$E_{\mathrm{NIMP}} \leq V_{\mathrm{NIMP}} I_{CC} t_p, (17)$$

where t_p is the NIMP pulse width. For a single XOR pair, the corresponding operation-level expression can be written as

$$E_{\mathrm{XOR,pair}} \approx 2E_{\mathrm{NIMP}} + E_{\mathrm{OR}}. (18)$$

However, because the main text does not numerically tabulate the NIMP pulse width or the OR-operation current/time product, we did not extract a single absolute electrical energy. Moreover, the encryption function in this platform is inherently realized through a multi-step XOR logic sequence rather than a bias-selectable secure-read mechanism. Therefore, a fair energy interpretation should include not only the device-level switching event itself, but also

the associated logic-flow overhead required for XOR evaluation, including intermediate logic operations and their supporting peripheral control. In this sense, the effective system-level overhead for security operation is intrinsically larger than that of a bias-programmable secure-read device such as the FITFET, even though a directly comparable absolute energy value is not explicitly provided in the original paper.

Park et al. (ref. 17)

Park et al. reported a concealable PUF using commercial V-NAND flash memory, in which the entropy source is generated by weak GIDL erase. The paper explicitly shows that a weak GIDL condition around V_{GIDL} = 3.0 V produces a suitable bimodal threshold-voltage distribution for PUF formation and also emphasizes that GIDL erase is a native operation of V-NAND flash memory, such that no additional functionality or circuitry is required. The relevant operation energies can therefore be written generically as

$$E_{\text{Park,GIDL}} = \int V_{\text{GIDL}} I_{\text{BL}}(t)\, dt, \quad (19)$$

$$E_{\text{Park,conceal}} = \int V_{\text{prog}}(t) I_{\text{prog}}(t)\, dt, \quad (20)$$

$$E_{\text{Park,read}} = \int V_{\text{read}} I_{\text{BL}}(t)\, dt. \quad (21)$$

Here, the concealment process corresponds to overwriting the PUF with user data, while the reveal process corresponds to re-establishing the PUF data through GIDL erase. Since the main text does not provide full numerical current transients or pulse widths for these operations, we did not back-calculate a single absolute energy value. The key distinction from the FITFET is that, although no extra circuitry is required, concealment/recovery still relies on an overwrite-and-reveal sequence, rather than on a bias-only secure read condition in the same synaptic state.

Supplementary Reference

1. Weng, Z., Zhao, L., Lee, C. & Zhao, Y. Phase Transitions and Anti-Ferroelectric Behaviors in $Hf_{1-x}Zr_xO_2$ Films. *IEEE Electron Device Lett.* **44**, 1780–1783 (2023).
2. Li, J. et al. Enhancing ferroelectric stability: wide-range of adaptive control in epitaxial HfO_2/ZrO_2 superlattices. *Nat. Commun.* **16**, 6417 (2025).
3. Shin, H. et al. Record-Low EOT(3.6Å) & Jleak(7×10-8A/cm2@0.8V) HZO by Dielectric-Selective Microwave Annealing. I*EEE Int. Reliab Phys. Symp. Proc.* 1–9 (2025) .
4. Zhang, F. et al. The Investigation of Reduced Variation Effect in FinFETs With Ultrathin 3-nm Ferroelectric $Hf_{0.5}Zr_{0.5}O_2$. *IEEE Trans. Electron Devices* **71**, 2876–2880 (2024).
5. Dongsu, K. et al. Low-Temperature Nanosecond Laser Process of HZO-IGZO FeFETs toward Monolithic 3D System on Chip Integration. *Adv. Sci.* **11**, 2401250 (2024).
6. Cho, J. W. et al. Atomic Layer Deposition of Epitaxial Ferroelectric $Hf_{0.5}Zr_{0.5}O_2$ Thin Films. *Adv. Funct. Mater.* **34**, 1–10 (2024).
7. Verbeeck, J. & Van Aert, S. Model based quantification of EELS spectra. *Ultramicroscopy* **101**, 207–224 (2004).
8. Lee, J. et al. Modulating the Ferroelectricity of Hafnium Zirconium Oxide Ultrathin Films via Interface Engineering to Control the Oxygen Vacancy Distribution. *Adv. Mater. Interfaces* **9**, 1–11 (2022).
9. Tang, R. et al. Region-Selective Oxygen Vacancy Engineering for Ferroelectric $Hf_{0.5}Zr_{0.5}O_2$Thin Films Processed at 300 °C. *ACS Appl. Mater. Interfaces* **17**, 53683–53690 (2025).
10. Goh, Y., Cho, S. H., Park, S. H. K. & Jeon, S. Oxygen vacancy control as a strategy to achieve highly reliable hafnia ferroelectrics using oxide electrode. *Nanoscale* **12**, 9024–9031 (2020).
11. Zhang, W. Di et al. Ultrahigh dielectric permittivity in $Hf_{0.5}Zr_{0.5}O_2$ thin-film capacitors. *Nat. Commun.* **16**, 1–10 (2025).
12. Gao, B. et al. Concealable physically unclonable function chip with a memristor array. Sci. Adv. 8, eabn7753 (2022).
13. Li, X. et al. A memristor-based unified PUF and TRNG chip with a concealable ability for advanced edge security. Sci. Adv. 11, 1–10 (2025).
14. Woo, K. S. et al. Tunable stochastic memristors for energy-efficient encryption and computing. Nat. Commun. 15, 1–9 (2024).
15. Kim, H. Y. et al. Chiroptical Synaptic Perovskite Memristor as Reconfigurable Physical

Unclonable Functions. ACS Nano 19, 691–703 (2025).

16. Cho, J. M. et al. Concealable physical unclonable function generation and an in-memory encryption machine using vertical self-rectifying memristors. Nanoscale Horizons 10, 113–123 (2024).

17. Park, S. H. et al. Concealable physical unclonable functions using vertical NAND flash memory. Nat. Commun. 16, 1–11 (2025).
