# Supplementary material for: A ferroelectric-ionic-trapping transistor for low power and secure neuromorphic computing
Source: Nat Commun. 2026 May 29;17:7831. doi: 10.1038/s41467-026-72971-y (PMC13439170; doi:10.1038/s41467-026-72971-y)
Supplement: Supplementary file 2 [file 41467_2026_72971_MOESM2_ESM.pdf]

nature portfolio

Peer Review File

A ferroelectric-ionic-trapping transistor for low power and secure neuromorphic computing

Corresponding Author: Professor Daewoong Kwon

This file contains all reviewer reports in order by version, followed by all author rebuttals in order by version.

Version 0:

Reviewer comments:

Reviewer #1

(Remarks to the Author)
This manuscript presents a ferroelectric-ionic-trapping transistor, featuring a switching between two modes, which enable low power synaptic and security function in one device. This is based on the trapping of charges by the inserted TiO_2 layer to mask the stored information at a specific range of gate voltage. The designed gate structure also allows for ionic migration at elevated gate biases to enhance the memory window. While the proposed device and working mechanism is interesting for the community of ferroelectric memory, the paper currently suffers from several issues, both in application and device characterization perspective, that need to be addressed before publication.

1. It is open to question whether the device can safeguard the stored information, just by masking the MW at a higher gate voltage. The key issue is how the hardware can distinguish between a normal user from an attacker. This probably needs an authentication system that adapts with the proposed hardware. The authors should provide more information on the corresponding security check working flow.

2. Following the question above, even though the hardware can identify a potential attacker and only offers the safe mode, it looks that the attacker can easily extract the stored information by manipulating the gate voltage, or by applying negative pulses. Please discuss on such possibilities.

3. The characterization of the device is mostly based on DC sweeping of the Id-Vg curve. While the hysteresis can reflect the memory behavior, the DC sweeping is still inherently different from the actual operation scheme, which is based on positive and negative gate pulses. The authors need to test more I-V results of the device based on pulsed program and erase, and show whether the shifted I-V curves merge up at higher Vg.

4. It is open to question whether the switching is damage free. By applying a larger read-bias, the erased state of the transistor may be disturbed. The authors need to show tests results of the read disturb.

5. Since the switching of the two modes is modulated by charge trapping, the authors also need to characterize the dynamics and the reliability of the switching. For the dynamics, the stabilization time of the modes after changing the read bias should be checked. For the reliability, the memory property of the device after extended switching cycles (like up to 1e9) should be studied.

6. From Fig.4e, it looks that ionic-enhanced mode cannot enable information masking, is that right? If so, the authors should clarify on this to avoid confusing the readers.

7. The authors need to provide more details for the simulation of the neuro-network. Is the non-linearity and the D2D variation of the device considered? If so, how to decide the parameters? Are they from literatures or extracted from the measurement?

8. The benchmark in Table S2 should be supplemented with more comprehensive evaluations, like area, power efficiency, and security etc.

Reviewer #2

(Remarks to the Author)
This manuscript reports a ferroelectric-ionic-trapping field-effect transistor (FITFET) based on HZO and a SiO_2/TiO_2 interface layer to achieve both energy-efficient neuromorphic computing and hardware-level security. The below questions must be answered:
1.How stable and non-destructive is the memory-window collapse under repeated mode toggling? Please offer evidence and clarify how long the concealed state persists after high-voltage removal.
2.Are the ferroelectric, trapping, and ionic mechanisms truly separable and reproducible across devices? The manuscript implicitly treats the three mechanisms as bias-addressable and independent, yet lacks quantitative analysis of their mutual cross-coupling. Please provide bias-boundary statistics and discuss cross-coupling effects across multiple devices.
3.Can the LFN-based defect depth interpretation be independently validated? Please correlate the LFN analysis with control structures or an additional experimental technique to support the proposed defect depth and mechanism assignment in this mixed ferroelectric–ionic system.
4.Since the rate of ion movement is significantly slower than dipole flipping, the operating speed of the device becomes an issue. What is the minimum pulse width for the Hybrid PGM?
5.The author did not evaluate the consistency of the device array in the main text. How would the results be affected if only some devices in the array failed to successfully enter Secure Mode? Relevant explanations could be provided.

Reviewer #3

(Remarks to the Author)
The authors report a ferroelectric–ionic–trapping field-effect transistor (FIT-FET) that integrates neuromorphic synaptic functionality with a hardware-native data concealment mechanism based on reversible memory window collapse.

The concept of bias-programmable plain and secure operation modes is timely and original, and the manuscript is supported by a comprehensive set of experimental and system-level analyses, including atomic-scale characterization, in-situ low-frequency noise spectroscopy, array-level demonstrations, and security-oriented neural-network simulations.

Overall, the work addresses an important challenge at the intersection of neuromorphic hardware and data security.

I can recommend this work for publication in Nature Communications after the following points are clarified.

Comment 1.
The terms "plain mode" and "secure mode" are central to the manuscript and are used repeatedly throughout the text. However, their definitions are somewhat distributed across multiple sections. For improved clarity, the authors are encouraged to explicitly define these two modes at their first appearance in the main text, clearly stating the corresponding read-bias conditions and the resulting electrical behavior.

Comment 2.
The role of the TiO_2 interlayer is discussed across several sections, including oxygen vacancy modulation, ionic switching, and interface engineering. While the explanations are thorough, the key takeaway may be diluted by repetition. For reader convenience, the authors may consider adding a single concise sentence summarizing the primary function of the TiO_2 layer in the FIT-FET, for example in the device structure section or concluding paragraph of the mechanism discussion.

Comment 3.
It should be noted that the p1/p2 intensity ratio reflects a relative trend in VO concentration rather than an absolute quantitative concentration. Explicitly acknowledging this limitation in the manuscript or the supplementary information would help readers interpret the EELS results more appropriately and avoid potential over-quantification.

Comment 4.
The authors are encouraged to expand the XPS-related discussion, particularly by more clearly describing the peak deconvolution procedure of the O 1s spectra, the physical meaning of each fitted component, and how the extracted oxide-deficient fraction is correlated with VO concentration.

Comment 5.
In general, most figures clearly specify the applied voltage conditions, such as amplitude and pulse width, which helps readers understand the measurement protocols. However, in some figures, this information is missing or only partially described, making it difficult for readers to immediately grasp the experimental conditions. This issue is particularly noticeable in the array-level measurement data, where multiple bias conditions are used for program, read, inhibit, and erase operations.

Version 1:

Reviewer comments:

Reviewer #1

(Remarks to the Author)
The authors have comprehensively answered my questions and addressed most of my concerns. The manuscript is probably publishable after further revisions below:

1. For question (1), the estimate of 3% accuracy drop is based on simulation of devices in good condition. If the device work continously in the safe mode with a lot of interface trapping effects, is it possible to cause further degradation of the accuracy due to Vth shift or increased low-frequency noise? Please provide some further discussions in this issue.

2. For question (8), the authors accepted part of my suggestion by adding more benchmark figures. While the safety of different PUF designs are hard to evaluate, I sitll recommend the authors to evaluate the energy or power consumption the PUF devices, especially in the safe mode, and compare it with literatures.

Reviewer #2

(Remarks to the Author)
The authors have addressed my comments and concerns.

Reviewer #3

(Remarks to the Author)
The authors have addressed all of my comments from the previous round. I now recommend for publication in Nature Communications as-is.

Version 2:

Reviewer comments:

Reviewer #1

(Remarks to the Author)
The authors have addressed my comments. There are no further issues on my part.

Open Access This Peer Review File is licensed under a Creative Commons Attribution 4.0 International License, which permits use, sharing, adaptation, distribution and reproduction in any medium or format, as long as you give appropriate credit to the original author(s) and the source, provide a link to the Creative Commons license, and indicate if changes were made.
In cases where reviewers are anonymous, credit should be given to 'Anonymous Referee' and the source.
The images or other third party material in this Peer Review File are included in the article's Creative Commons license, unless indicated otherwise in a credit line to the material. If material is not included in the article's Creative Commons license and your intended use is not permitted by statutory regulation or exceeds the permitted use, you will need to obtain permission directly from the copyright holder.
To view a copy of this license, visit https://creativecommons.org/licenses/by/4.0/

Revision and Correction (NCOMMS-25-98264A)

Dear Reviewers,

We, the authors, appreciate the time and effort that the reviewers dedicated to providing feedback on our manuscript and are grateful for the insightful comments on our work. We read the comments and questions carefully and prepared a response sheet for them. Please refer to the responses below. The responses that need to be addressed in the revised manuscript are highlighted so that the reviewers can trace them with more ease. Also, the overall sentences have been refined with more concise expressions and better English grammar. We heartily thank all the comments and coordination again.

Reviewer #1

This manuscript presents a ferroelectric-ionic-trapping transistor, featuring a switching between two modes, which enable low power synaptic and security function in one device. This is based on the trapping of charges by the inserted TiO_2 layer to mask the stored information at a specific range of gate voltage. The designed gate structure also allows for ionic migration at elevated gate biases to enhance the memory window. While the proposed device and working mechanism is interesting for the community of ferroelectric memory, the paper currently suffers from several issues, both in application and device characterization perspective, that need to be addressed before publication.

Response:

We sincerely thank reviewer for the careful evaluation of our manuscript and for the constructive comments. We appreciate the reviewer's recognition of the potential interest of the proposed ferro-ionic-trapping transistor to the ferroelectric memory community, as well as the insightful concerns raised from both application and device-characterization perspectives.

The comments have been very helpful in identifying points that require further clarification and strengthening. In the revised manuscript, we have addressed each issue in detail by providing additional discussions, analyses, and clarifications, as described in the point-by-point responses below.

We, the authors, send the deepest appreciation for the encouragement and comments again.

1. It is open to question whether the device can safeguard the stored information, just by masking the MW at a higher gate voltage. The key issue is how the hardware can distinguish between a normal user from an attacker. This probably needs an authentication system that adapts with the proposed hardware. The authors should provide more information on the corresponding security check working flow.

Response:

Thank you for your comment. We sincerely thank the reviewer for this insightful feedback regarding the practical implementation of our security mechanism. We agree that traditional security frameworks often rely on active authentication to distinguish between authorized and unauthorized users. However, the unique advantage of the FITFET is its 'Safe-by-Design' capability, which effectively suppresses image reconstruction vulnerability with marginal impact on inference accuracy. This approach provides hardware-native protection without the computational overhead or complexity of a real-time authentication layer. Instead of a dynamic security check, we propose a streamlined workflow based on a fixed hardware configuration as follows: To achieve this, the system can be configured to permanently shift the operating window of sensitive layers (e.g., L1) into the Secure Mode ($V_{GS} \approx 1.25\text{~}1.5$ V) by adjusting the relative Source Line (V_{SL}) and Bit Line (V_{BL}) voltage. This ensures that even when input data is encoded in a standard analog range (0 ~ 1 V), the effective V_{GS} of the sensitive layer remains within the MW collapse region, while other layers continue to operate in Plain Mode. Notably, our system-level simulations confirm that while this permanent concealment effectively obscures synaptic weights from adversarial readout, the resulting classification accuracy drop is only approximately 3% (maintaining an overall accuracy of ≈ 95%). Consequently, since performance degradation is negligible compared to the robust security gained, the hardware can be pre-set to this secure state for all users. In this scenario, the weights remain physically concealed from any reconstruction attempt (such as MI attacks) for everyone, while authorized inference continues with high fidelity. In accordance with the reviewer's comment, we have added a detailed discussion regarding this 'Safe-by-Design' workflow and its efficiency in the revised manuscript. We believe this provides a more intrinsically secure route for privacy-preserving neuromorphic hardware. Thank you again for your careful review.

Corresponding change in manuscript: Yes

Page 20 in the manuscript:

Unlike traditional frameworks that require active authentication to distinguish between users, the FITFET-based system offers a 'Safe-by-Design' approach that eliminates the need for a complex, real-time security check workflow. This workflow can be achieved by adjusting the relative V_{SL} and V_{BL} voltages to permanently shift the operating window of sensitive layers (e.g., L1) into Secure Mode ($V_{GS} \approx 1.25 - 1.5$ V). Such a fixed hardware configuration ensures that synaptic weights remain physically concealed from any adversarial reconstruction for all users, while maintaining high-fidelity authorized inference with negligible computational overhead.

Location of Change: Page 20 in the manuscript.

2. Following the question above, even though the hardware can identify a potential attacker and only offers the safe mode, it looks that the attacker can easily extract the stored information by manipulating the gate voltage, or by applying negative pulses. Please discuss on such possibilities.

Response:

Thank you for your comment. We appreciate the reviewer's constructive feedback regarding the robustness of our system against adversarial physical manipulation, which is indeed a critical aspect of hardware security. However, we argue that extracting meaningful information from the FITFET array is practically unfeasible due to several hardware-level constraints and the inherent physical properties of the device as follows: First, a potential attacker does not possess specific information regarding the system architecture, such as the network size, layer structure, or the number of layers utilized. Furthermore, device-specific characteristics, array configuration, and precise operating voltages are not disclosed, creating a significant information asymmetry that poses a fundamental barrier to any unauthorized readout. Beyond these structural barriers, to reconstruct original data, an attacker must obtain precise input (V_{GS}) and synaptic weights. Since the exact voltage difference between Secure and Plain Mode is unknown, searching for the specific gate voltage window that reveals the memory window would require an extreme expenditure of time and computational resources. Moreover, this security margin is further reinforced by modulating the source line (V_{SL}) and bit line (V_{BL}) voltage. This allows the system to dynamically shift the voltage difference between modes, effectively making the target operating window a moving target for any brute-force attempt. Crucially, applying any voltage that differs significantly from the calibrated operating range results in the permanent loss or perturbation of stored data. Rather than revealing information, such adversarial bias manipulation triggers the destructive erasure or depression of the ferroelectric and ionic states, effectively self-protecting the data from being compromised. In summary, the combination of physical data concealment and the lack of systemic information makes the FITFET a highly robust platform against adversarial readout. In accordance with the reviewer's comment, we have updated the manuscript to include a detailed discussion on these security aspects to enhance the clarity of our hardware's protective mechanisms. Thank you again for your careful review.

Corresponding change in manuscript: Yes

Page 20 in the manuscript:

Furthermore, the FITFET array is highly robust against physical adversarial manipulation due to significant information asymmetry regarding the system architecture; a potential attacker lacks critical knowledge of the network size, layer structure, and particularly the exact voltage difference between Plain and Secure Modes. The security is further reinforced by modulating the relative V_{SL} and V_{BL} voltages, which allows the system to shift the operating window and effectively present a 'moving target' for any brute-force attempt. Crucially, such indiscriminate voltage manipulation outside the calibrated range carries the inherent risk of triggering the destructive erasure or perturbation of ferroelectric and ionic states, thereby physically self-protecting the stored information from being compromised.

Location of Change: Page 20 in the manuscript.

3. The characterization of the device is mostly based on DC sweeping of the I_d-V_g curve. While the hysteresis can reflect the memory behavior, the DC sweeping is still inherently different from the actual operation scheme, which is based on positive and negative gate pulses. The authors need to test more *I-V* results of the device based on pulsed program and erase, and show whether the shifted *I-V* curves merge up at higher V_g.

Response:

We thank the reviewer for the insightful comment regarding the difference between DC-sweep-based characterization and the actual pulse-based operation scheme. First, we note that the pulse-based programming characteristics of the device are already reflected in Supplementary Figs. 16 and 17, which present retention and speed measurements performed using pulsed program and erase operations. These results allow one to infer that, under elevated gate bias conditions, the device undergoes a memory-window collapse similar to that observed in the DC I_D–V_{GS} characteristics, and that the memory window can re-emerge in the hybrid (ionic-contribution) regime at higher bias conditions.

We acknowledge, however, that in these measurements the read-voltage range was intentionally limited to below 1 V, and therefore the full merging of the shifted *I–V* curves at higher V_{GS} were not explicitly shown. This choice was made because excessively extending the read-voltage range after pulsed programming can itself perturb the stored state, which is undesirable for accurately evaluating retention and speed characteristics. As such, a conservative read range is required to avoid read-disturb effects in these measurements.

That said, we fully recognize the reviewer's concern regarding the need to explicitly demonstrate the merging behavior of the *I–V* curves at higher V_{GS}. In response, we have performed additional measurements in which the read-voltage range was deliberately extended for selected cases to clearly show the convergence of the *I–V* curves (Revised Fig. S17f). These results have been added to the revised manuscript and Supplementary Information to provide a more complete and intuitive understanding of the pulse-based operation and its correspondence with the DC characteristics.

We believe that these additions clarify the relationship between DC and pulsed measurements and help readers better understand the device operation across different bias regimes.

Corresponding change in manuscript: Yes

Fig. S17 in the supplementary:

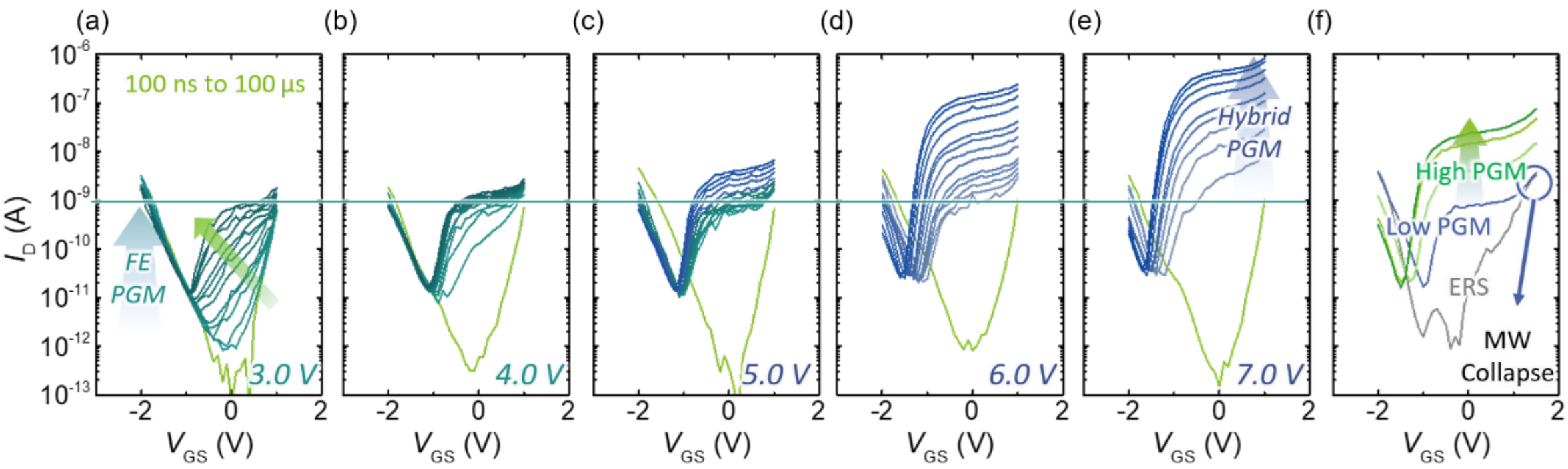

S17. Pulse bias-dependent transition from ferroelectric to hybrid ferro-ionic switching

Transfer characteristics (I_D-V_{GS}s) of the FITFET measured under V_{PGM} voltages of (a) 3.0 V, (b) 4.0 V, (c) 5.0 V, (d) 6.0 V, and (e) 7.0 V. (f) Transfer characteristics measured over an extended read range, confirming the MW collapse is also observed under pulse-based operation at elevated V_{GS} conditions. The FITFET exhibits a distinct transition from ferroelectric-dominant switching at low V_{PGM}s to hybrid ferro-ionic switching at higher V_{PGM}s, indicating the progressive activation of ionic migration and the coexistence of dipole- and vacancy-driven conduction mechanisms.

Location of Change: Fig. S17 in the supplementary.

4. It is open to question whether the switching is damage free. By applying a larger read-bias, the erased state of the transistor may be disturbed. The authors need to show tests results of the read disturb.

Response:

We thank the reviewer for the constructive comment regarding the possibility of read disturb under a larger read bias. To directly address this concern, we performed a dedicated read disturb evaluation at the array-level, focusing on potential disturbance mechanisms that may arise during practical read operation under the same bias conditions used for concealment.

As noted by the reviewer, cells sharing the same word line (WL) with the target cell are exposed to the secure-read voltage and may therefore be susceptible to unintended state changes. In particular, the most critical concern is the (i) disturbance of an erased (ERS) cell toward the programmed (PGM) state under repeated application of the secure-read bias (1.5 V) on the selected WL. To comprehensively evaluate read-disturb behavior under array-level operation, we additionally examined (ii) possible current drift of a PGM cell sharing the selected WL under the same secure-read stress, and (iii) unintended erasure of a PGM cell caused by the small negative bias (-0.5 V) applied to unselected WLs, which is used during read operation to suppress residual off-current contributions from cells sharing the same bit line (BL).

For this evaluation, cells in the array were first initialized to either the ERS or PGM state. Repeated pulses with a width of 1μs were then applied up to 10^6 cycles using the WGFMU (B1500A, Keysight), following the actual array read conditions. A secure-read voltage of V_{READ} = 1.5 V was applied to the selected WL, while a negative bias of V_{UNSEL} = -0.5 V was applied to the unselected WLs. After a given number of pulses, the drain current was measured under a non-disturbing read condition (V_{READ} = 0.5 V, V_D = 0.1 V) to monitor any change in the stored state. The measurement results show that, in all three cases, the initially programmed and erased states remain stable throughout the repeated pulse application, with no evidence of unintended programming, erasure, or state degradation. These results confirm that the larger read bias used for secure read operation does not induce read disturbance under array-level operating conditions. The corresponding schematic illustration and experimental results have been added as Fig. S24.

Corresponding change in manuscript: Yes

Fig. S24 in the supplementary:

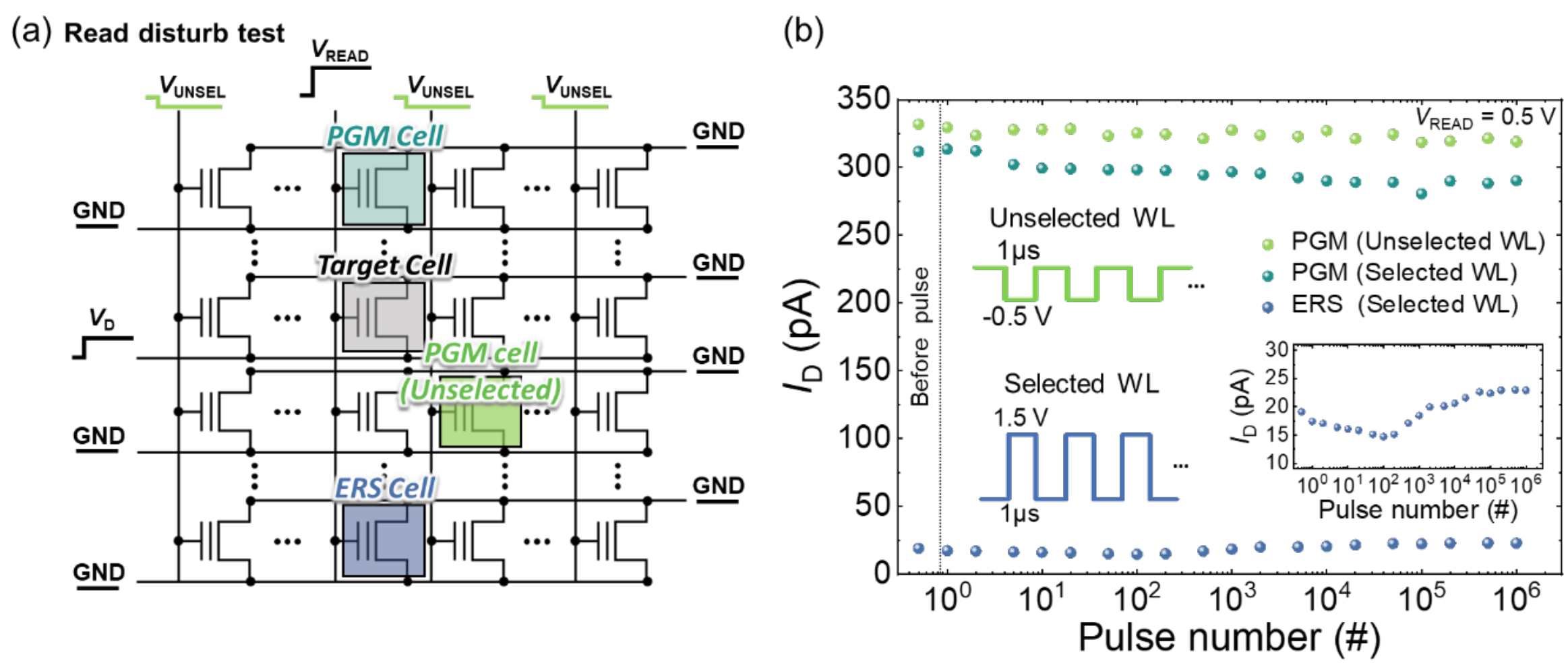

S24. Read-disturb evaluation under array-level read operation

(a) Schematic illustration showing different read-disturb contributions during the read operation in the FITFET array, including repeated secure-read bias on the selected WL and negative bias on unselected WLs. (b) Drain current stability (V_{READ} = 0.5 V) under repeated pulses (1 µs) up to 10^6 cycles, where a secure-read voltage (1.5 V) was applied to the selected WL and a negative bias (V_{UNSEL}= -0.5 V) was applied to the unselected WLs. The results confirm that neither bias condition induces disturbance of the erased or programmed states.

Page 16 in the manuscript:

Furthermore, array-level disturbance measurements confirm that repeated read operations do not induce unintended state changes in neighboring or unselected cells, demonstrating disturbance-free operation at the array levels (Supplementary Fig. 24).

Location of Change: Page 16 in the manuscript. Fig. S24 in the supplementary.

5. Since the switching of the two modes is modulated by charge trapping, the authors also need to characterize the dynamics and the reliability of the switching. For the dynamics, the stabilization time of the modes after changing the read bias should be checked. For the reliability, the memory property of the device after extended switching cycles (like up to 1e9) should be studied.

Response:

We thank the reviewer for the valuable comment regarding the dynamics and reliability of the mode switching governed by charge trapping. To address this point, we performed additional measurements in which the device was repeatedly toggled between the plain mode and the secure mode to examine the stability and dynamics of each mode. These results confirm that both modes stabilize reproducibly after changes in the read bias and operate in a stable manner during repeated mode transitions (Fig. S19). For the ferroelectric-dominant and trap-mediated states, the mode-toggling stability was evaluated using repeated DC transfer measurement. In contrast, for the hybrid state involving ionic contributions, repeated DC sweeping becomes unstable; therefore, AC pulse-based measurements were employed to assess repeated mode toggling. The consistent behavior observed under both DC- and AC-based evaluations confirms that the mode transitions remain stable and reversible regardless of the measurement scheme. We verified that simple variations within the read-bias range do not induce unintended transitions or instability between modes, confirming robust mode selectivity. Furthermore, we performed a statistical analysis across multiple devices that were carried out, confirming that device-to-device variation is well controlled and remains minimal (Fig. S18).

In addition, as suggested by the reviewer, we further evaluated the reliability of the device by examining its memory characteristics under extended switching cycles (Fig. S20). The supplementary results demonstrate that the memory properties of the FITFET remain well preserved after repeated mode switching, supporting the reliability of the proposed trapping-modulated operation scheme. These additional data and discussions have been incorporated into the revised manuscript and Supplementary Information. Lastly, we acknowledge the reviewer's suggestion of evaluating endurance up to 10^9 switching cycles. However, reaching 10^9 cycles is highly challenging for Si-channel FeFET because it requires further process/stack optimization and dedicated reliability engineering [R1-R4], which is beyond the scope of this study. Importantly, our demonstrated 10^7-cycle endurance already covers a practically relevant regime for neuromorphic inference, since mode switching occurs at the level of access control (e.g., session-based or time-limited reveal), not at every MAC operation. These points and the corresponding data are included in the revised manuscript and supplementary (Figs. S19–S20).

[R1] Suzuki, Kunifumi, et al. "High-endurance FeFET with metal-doped interfacial layer for controlled charge trapping and stabilized polarization." 2023 International Electron Devices Meeting (IEDM) (2023).

[R2] Zhou, Yuejia, et al. "Ferroelectric and interlayer co-optimization with in-depth analysis for high endurance FeFET." 2022 International Electron Devices Meeting (IEDM) (2022).

[R3] Tan, Ava Jiang, et al. "Ferroelectric HfO_2 memory transistors with high-κ interfacial layer and write endurance exceeding 10^{10} cycles." IEEE Electron Device Letters 42.7 (2021).

[R4] Zhou, Yuejia, et al. "A Reliable 2 bit MLC FeFET with High Uniformity and 10^9 Endurance by Gate Stack and Write Pulse Co-optimization." 2024 IEEE European Solid-State Electronics Research Conference (ESSERC) (2024).

Corresponding change in manuscript: Yes

Fig. S18 in the supplementary:

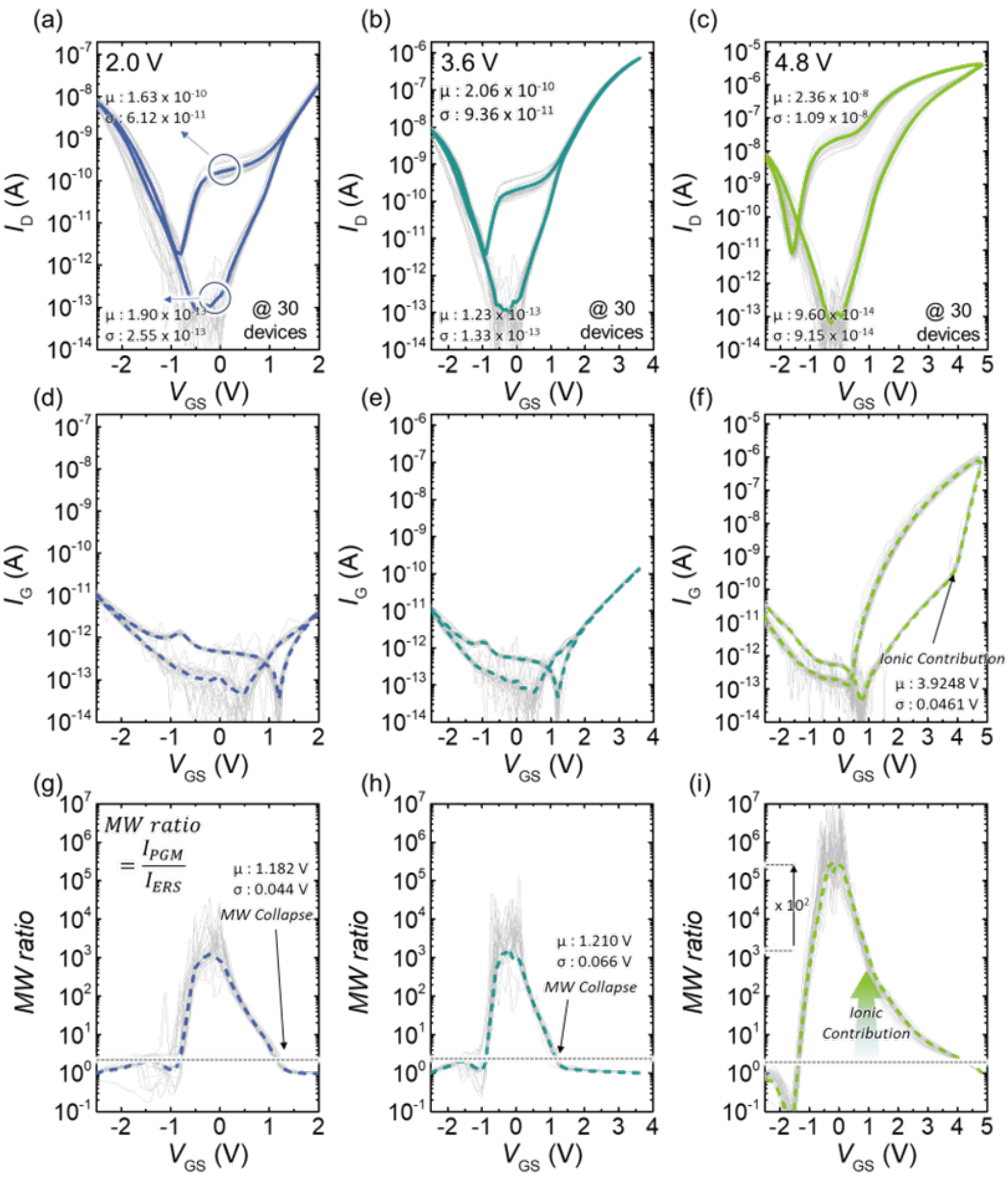

S18. Device-to-device uniformity of ferroelectric and hybrid ferro-ionic switching

Transfer characteristics ($I_{D,G}$-V_{GS}s) measured from 30 individual devices under (a, d) the FE-dominant, (b, e) trap-mediated and (c, f) hybrid ferro-ionic regimes, respectively. MW ratio measured at V_{GS} = 0 V under (g) FE-dominant, (h) trap-mediated, and (i) hybrid regime, showing consistent MW collapse at V_{GS} ≈ 1.18–1.21 V and re-opening of the MW beyond ~3.9 V across devices

Fig. S19 in the supplementary:

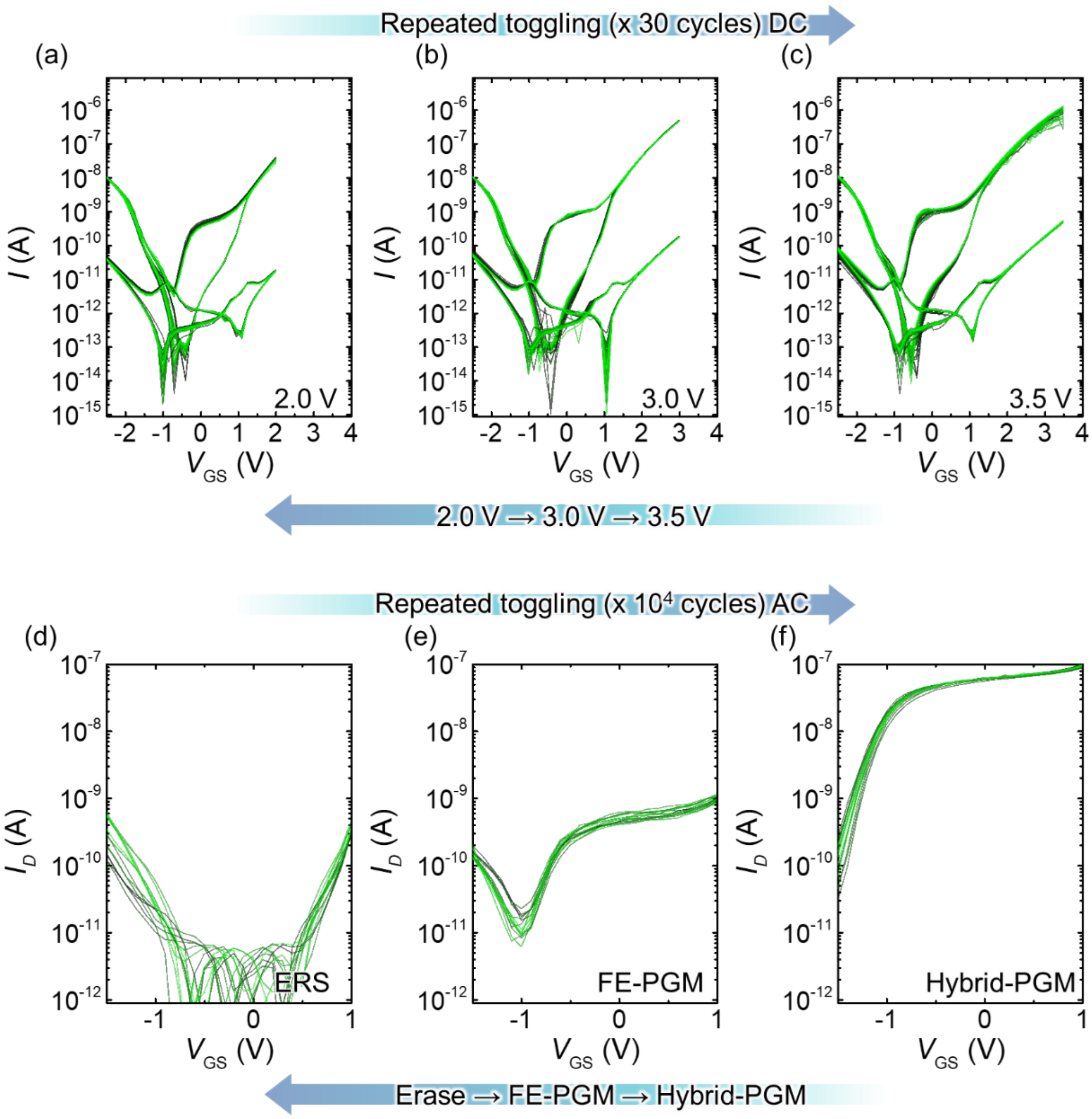

S19. Repeated mode-toggling characteristics of the FITFET

(a-c) DC Transfer characteristics measured under repeated toggling between 2.0 V, 3.0 V, and 3.5 V. The bias sequence 2.0 V → 3.0 V → 3.5 V was consecutively applied and repeated 30 cycles. (d-f) Pulse based transfer characteristics measured under repeated toggling between Erase, FE-PGM, and Hybrid-PGM states. Unlike the FE-dominant and trap-mediated states, the hybrid-PGM state involves ionic contributions, making repetitive DC sweeping impractical. Therefore, AC pulse operation was used as the method to repeatedly toggle. The negligible variation among the transfer curves confirms stable and reversible mode transitions under repeated DC and AC modulation.

Fig. S20 in the supplementary:

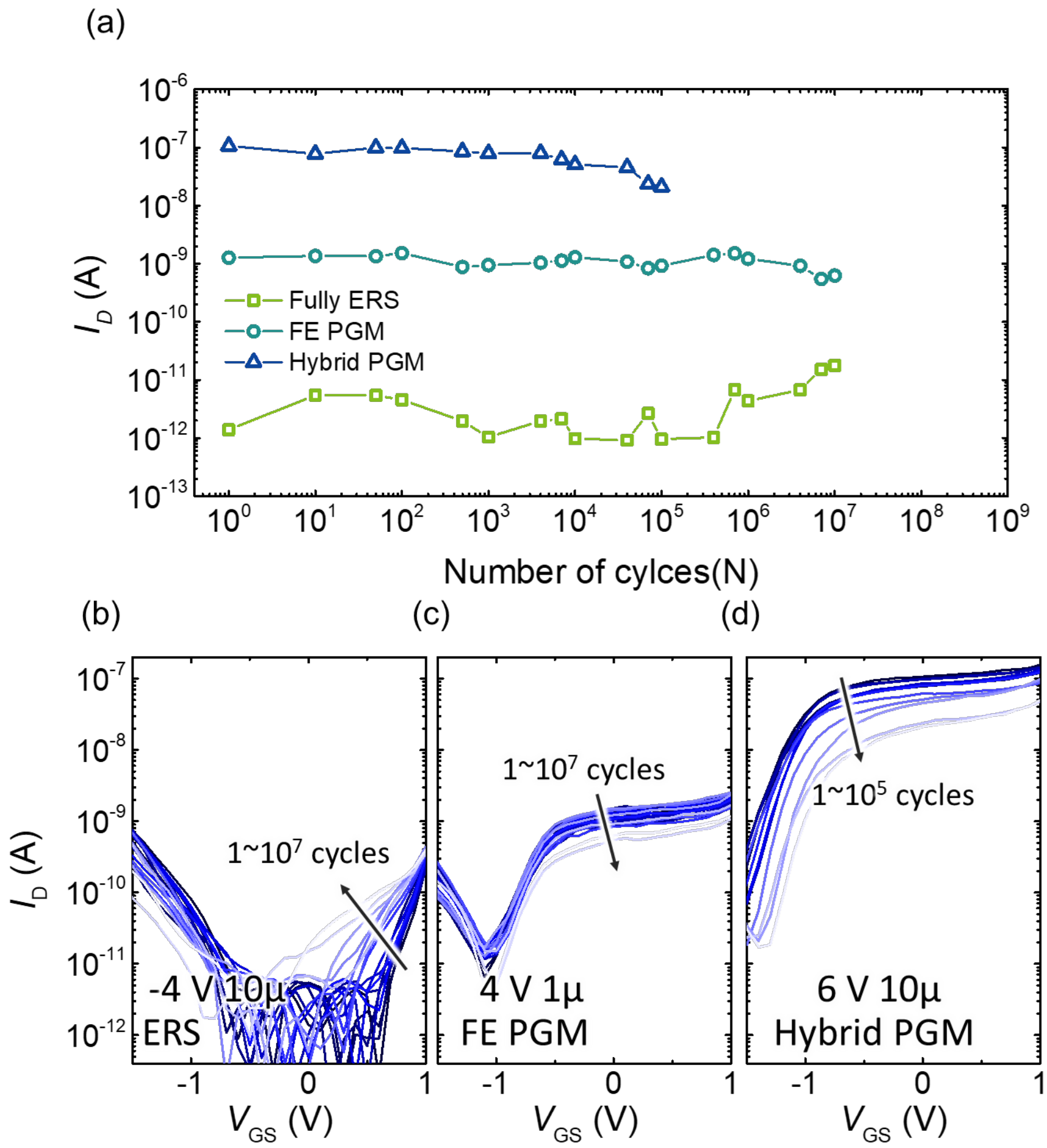

S20. Endurance characteristics of FITFET under different programming modes

(a) I_D evolution as a function of the number of switching cycles for fully ERS (-4 V, 10 μs), FE PGM (4 V, 1 μs), and Hybrid PGM (6 V, 10 μs), demonstrating stable states without noticeable degradation. (b-d) Corresponding I_D-V_{GS} measured during endurance cycling for (b) fully ERS, (c) FE PGM, and (d) Hybrid PGM states.

Page 13 in the manuscript:

The robustness of the bias-controlled mode transition was further confirmed through repeated mode-toggling measurements and long-term endurance measurements in Supplementary Fig. S19 and S20.

Location of Change: Page 13 in the manuscript. Figs. S18-20 in the supplementary.

6. From Fig.4e, it looks that ionic-enhanced mode cannot enable information masking, is that right? If so, the authors should clarify on this to avoid confusing the readers.

Response:

We thank the reviewer for pointing out the potential confusion regarding the ionic-enhanced mode (hybrid mode) shown in Fig. 4e. The reviewer is correct that the ionic-enhanced mode does not enable information masking. We would like to clarify that this mode is not intended to serve as the secure (concealment) operation mode of the FITFET. Instead, it is deliberately accessed under high-voltage conditions to demonstrate the presence and electrical activity of ionic species, such as oxygen vacancies, within the device stack. We acknowledge that in the original manuscript, the role and purpose of this ionic-enhanced (hybrid) regime were not sufficiently explained, which may have contributed to this confusion. In this regime, the device exhibits enhanced conductance modulation driven by ionic migration, which can be utilized as an additional synaptic modulation mechanism available only under elevated bias conditions. This operating region is therefore shown to illustrate the ferroelectric-ionic nature of the FITFET and its extended functional space, rather than to realize memory concealment.

The primary operating modes of the FITFET remain the plain mode (ferroelectric switching) and the secure mode (trap-mediated memory-window collapse), both of which operate at lower bias conditions and do not rely on ionic migration. In typical operation scenarios, only these two modes are used to achieve accurate inference and protected readout. The ionic-enhanced mode represents a special, high-bias operating condition that may be exploited in applications requiring a wider dynamic range through ionic-assisted synaptic functionalities.

To avoid confusion, we have clarified this distinction in the revised manuscript and figure discussion, explicitly stating that information concealment is exclusively realized through the secure mode and not through the ionic-enhanced regime.

Corresponding change in manuscript: Yes

Page 13 in the manuscript:

At higher pulse voltages (Fig. 4e), incremental pulse measurements were performed using 5.5 V to 7 V for potentiation and -3.5 V to -5 V for depression to probe the hybrid ferro-ionic switching operation beyond the purely ferroelectric switching observed in Fig. 4c. It should be noted that this hybrid ferro-ionic regime is not intended to enable information masking, but rather to explicitly probe the contribution of ionic species under high-bias conditions and to demonstrate an additional synaptic modulation—potentially useful for applications requiring a wider dynamic range—that is accessible only in this elevated-bias regime. Under these conditions, the FITFET exhibits linear and symmetric current responses even within the secure mode region, which is attributed to excessive field-driven V_O migration across the HZO. In the plain mode region, the first pulse induces a steep current increase due to ferroelectric polarization switching, followed by a gradual and nearly linear evolution,

indicating a transition toward the ferro-ionic hybrid state. These results confirm that when the applied bias is low, the FITFET can reliably store and conceal synaptic weights, whereas under sufficiently high bias, V_O migration becomes activated—serving as a supplementary high-bias operation mode rather than a concealment mechanism and providing direct physical evidence of the underlying ionic contribution to the encryption mechanism.

Location of Change: Page 13 in the manuscript.

7. The authors need to provide more details for the simulation of the neuro-network. Is the non-linearity and the D2D variation of the device considered? If so, how to decide the parameters? Are they from literatures or extracted from the measurement?

Response:

Thank you for your comment. We appreciate the reviewer's insightful observation that incorporating device non-idealities into system-level simulations is essential for accurately predicting hardware-level performance. We clarify that both device non-linearity and device-to-device (D2D) variations were comprehensively considered in our simulation framework as follows: Specifically, all parameters used in the simulation were extracted directly from our experimental measurements of the fabricated FITFET devices, rather than being sourced from general literature. This approach ensures that the simulation results closely reflect the actual physical behavior of our hardware. Furthermore, the D2D variations were modeled based on the statistical distribution, specifically using the coefficient of variation (σ/μ) derived from the measured conductance states of the FITFET array. Finally, as detailed in Fig. S18, which provides statistical data and the extraction process for these parameters, we ensured that the reported accuracy (98% for Plain Mode and 95% for Secure Mode) is a realistic representation of our hardware's capability. By applying these measured variations and non-linearities to the weight mapping process, our framework provides a robust prediction of system-level performance under real-world constraints. In accordance with the comment pointed out by the reviewer, we have added further clarifications regarding these simulation parameters and the parameter extraction process in the revised manuscript. We believe this enhances the transparency and reliability of our performance evaluation. Thank you again for your careful review.

Corresponding change in manuscript: Yes

Page 17 in the manuscript:

To ensure a realistic prediction of hardware-level performance, our system-level simulation framework comprehensively incorporates measured device non-idealities, including non-linearity and device-to-device (D2D) variations. Rather than relying on literature-sourced values, all simulation parameters were directly extracted from experimental measurements of the fabricated FITFET devices. Specifically, D2D variations were modeled using a statistical distribution based on the coefficient of variation (σ/μ) derived from the measured conductance states (see Supplementary Fig. 18 for details). This measurement-driven approach confirms that the reported recognition accuracies faithfully reflect the actual physical capabilities of the FITFET array under real-world constraints.

Location of Change: Page 17 in the manuscript.

8. The benchmark in Table S2 should be supplemented with more comprehensive evaluations, like area, power efficiency, and security etc.

Response:

Thank you for this constructive suggestion. We agree that a benchmark table is most informative when it includes not only functional demonstrations but also practical metrics. At the same time, we note that the literature addressing hardware security spans different problem definitions and threat models. In particular, many prior works based on physical unclonable functions (PUFs) or concealment primitives focus on device authentication, key generation, or challenge–response protocols, whereas our work targets a different but complementary objective: physical concealment of analog synaptic weights at the device/array level to mitigate readout-driven model inversion. Therefore, a strict 1-to-1 quantitative comparison across all metrics is not always possible, because the system roles, required peripherals, and evaluation protocols differ across categories. Nevertheless, to address the reviewer's request, we have expanded Table S2 to provide a more comprehensive and transparent comparison.

Specifically, we added the following items where they can be meaningfully reported or estimated on a consistent basis. First, we added array size (demonstrated scale) for each work, because scalability is a key practical metric for neuromorphic/hardware-security implementations. Second, we added the concealment voltage required to activate the security function. In our case, concealment is realized purely by the read-bias condition (i.e., secure read), and therefore the relevant comparison parameter is the bias magnitude; the scheme is compatible with short read pulses (including ns-scale reads), so we report the voltage level used for concealment rather than a specific pulse width/energy-per-operation. Third, because the prior works in Table S2 target different security objectives (e.g., PUF-based authentication/key generation, obfuscation, concealment primitives, etc.), a single universal security score is not meaningful. Instead, we supplemented the benchmark using the security metrics that each paper reports and is appropriate for its threat model, and we listed those metrics side-by-side (e.g., reliability/uniqueness for PUF-type works, reconstruction/privacy-related metrics for concealment/obfuscation-type works). Overall, the updated Table S2 provides a clearer and more comprehensive benchmark that reflects both device/array practicality and security relevance, while transparently acknowledging differences in security definitions across the literature.

Corresponding change in manuscript: Yes

Device Structure	Material system	Encryption source	Concealment mechanism	CMOS compatibility	Array size	Concealment Voltage	Security	Ref. No
2-terminal memristor	HfO_x/TaO_x	Oxygen vacancies	Filament formation	Good	64 x 128 array	2.2 V	Concealed BER > 30%	[51]
2-terminal memristor	HfO_x	Oxygen vacancies	Filament formation	Good	256 x 64 array	3.0 V	Concealed BER > 40.51%	[52]
2-terminal memristor	HfO_x	Oxygen vacancies	Filament formation	Good	4 x 4 array	-3.5 V	Inter-HD 50.6%	[53]
2-terminal memristor	$R\text{-}(MBA)_2PbI_{4(0.7)}Br_{4(0.3)}$	Halide vacancies	Filament formation	Poor	10 x 10 array	- 2.0 V	Inter-HD 42.3%	[54]
3D vertical RRAM	$Al{:}HfO_2/Ta_2O_5$	Oxygen vacancies	Filament formation	Good	4 x 4 x 3 3D array	12 V	Inter-HD 50.63%	[55]
3D vertical NAND Flash	ONO	BL doping depth	Weak-GIDL erase	Good	> 100 layers	> 15 V	Concealed BER 49.4%	[56]
3-terminal FET	$HfO_2/TiO_2/SiO_2$	Interface traps	Trap-modulated screening	Good	24 x 12 array	1.25 V	CosSim ≈ 0.0	This work

Location of Change: Table S2 in the supplementary.

Reviewer #2

This manuscript reports a ferroelectric-ionic-trapping field-effect transistor (FITFET) based on HZO and a SiO_2/TiO_2 interface layer to achieve both energy-efficient neuromorphic computing and hardware-level security.

Response:

We thank reviewer for the thorough and technically insightful assessment of our manuscript. We appreciate the reviewer's valuable comments on device reliability, switching mechanisms, and the interpretation of the LFN analysis. These points have helped us to further clarify the physical mechanisms and strengthen the experimental and system-level discussions.

In the revised manuscript, we address each of the reviewer's comments in detail and provide additional explanations and clarifications where necessary. Point-by-point responses are provided below, and corresponding revisions have been made in the manuscript.

We, the authors, send the deepest appreciation for the encouragement and comments again.

1. How stable and non-destructive is the memory-window collapse under repeated mode toggling? Please offer evidence and clarify how long the concealed state persists after high-voltage removal.

Response:

We thank the reviewer for the valuable comment regarding the stability and non-destructive nature of the memory-window collapse under repeated mode toggling.

In response, we performed additional measurements to evaluate the stability of the memory-window collapse during repeated toggling between the plain mode and the secure mode. These results demonstrate that both operating modes stabilize reproducibly after changes in the read bias and remain robust under repeated mode transitions (Fig. S19). For the ferroelectric-dominant and trap-mediated regimes, mode stability was evaluated using repeated DC transfer measurements. In contrast, because repeated DC sweeping is not suitable for the hybrid regime involving ionic contributions, AC pulse-based measurements were instead employed to assess its mode stability. The consistent and reversible responses observed across both measurement schemes confirm that the mode transitions are inherently stable. Furthermore, we confirmed that variations within the designated read-bias range do not trigger unintended mode switching or instability, underscoring the strong selectivity of each operating mode. In addition, to assess long-term reliability, we additionally conducted endurance measurements under extended switching cycles, as shown in Fig. S20. The results confirm that the Fully ERS, FE-PGM, and Hybrid-PGM states are all well maintained even after repeated cycling.

With respect to the reviewer's question regarding the device behavior after removal of the secure mode voltage, Fig. 4b shows that all programmed states maintain stable nonvolatile retention, indicating that the stored memory state remains unchanged after the secure-mode voltage is removed. When the read condition is returned to the plain-mode bias, the original memory window is reliably recovered without degradation.

Overall, these results demonstrate that the memory-window collapse induced in the secure mode is stable, reversible, and non-destructive, and that the concealment operation does not adversely affect the long-term data integrity of the device.

Corresponding change in manuscript: Yes

Fig. S19 in the supplementary:

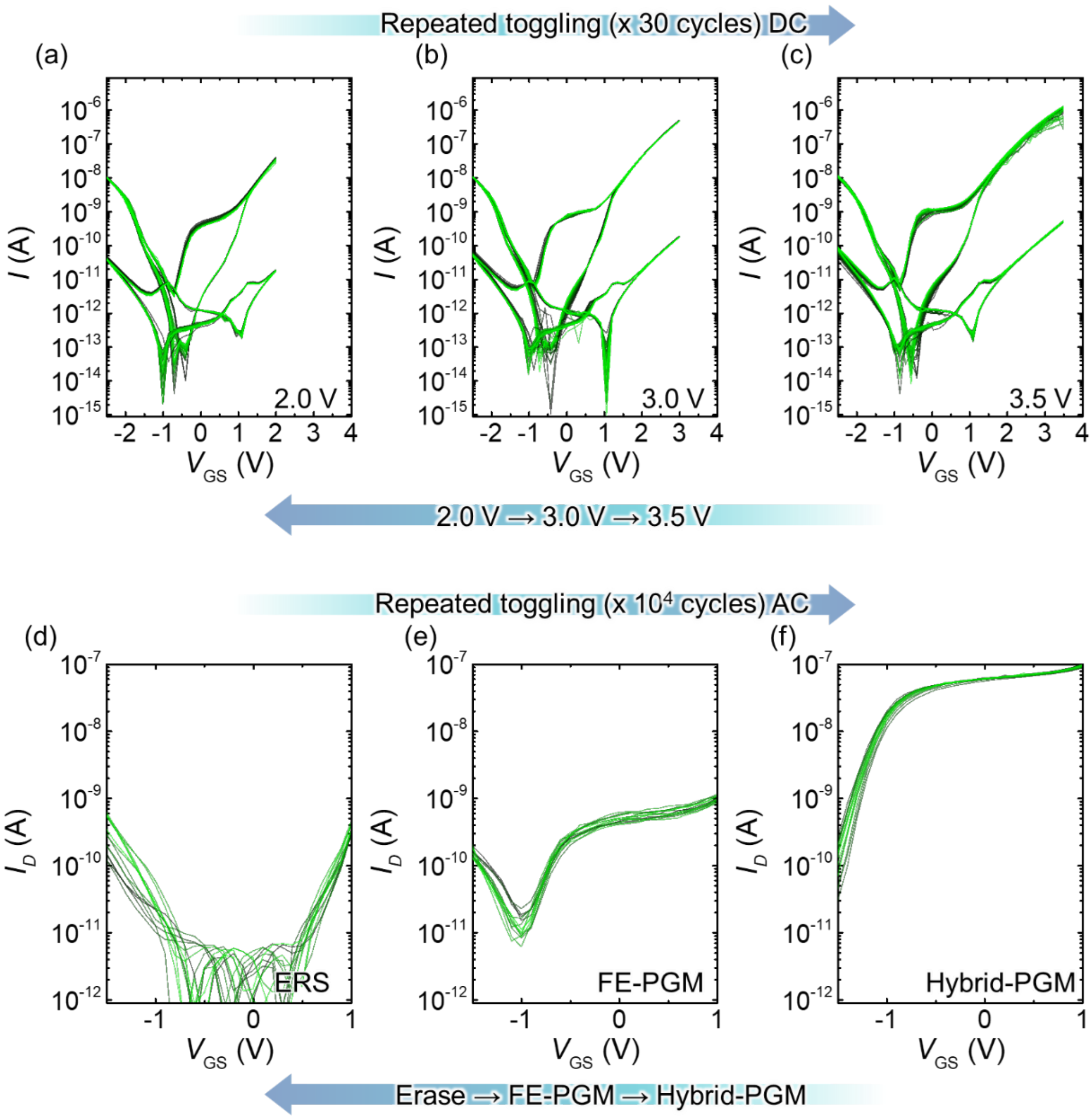

S19. Repeated mode-toggling characteristics of the FITFET

(a-c) DC Transfer characteristics measured under repeated toggling between 2.0 V, 3.0 V, and 3.5 V. The bias sequence 2.0 V → 3.0 V → 3.5 V was consecutively applied and repeated 30 cycles. (d-f) Pulse based transfer characteristics measured under repeated toggling between Erase, FE-PGM, and Hybrid-PGM states. Unlike the FE-dominant and trap-mediated states, the hybrid-PGM state involves ionic contributions, making repetitive DC sweeping impractical. Therefore, AC pulse operation was used as the method to repeatedly toggle. The negligible variation among the transfer curves confirms stable and reversible mode transitions under repeated DC and AC modulation.

Fig. S20 in the supplementary:

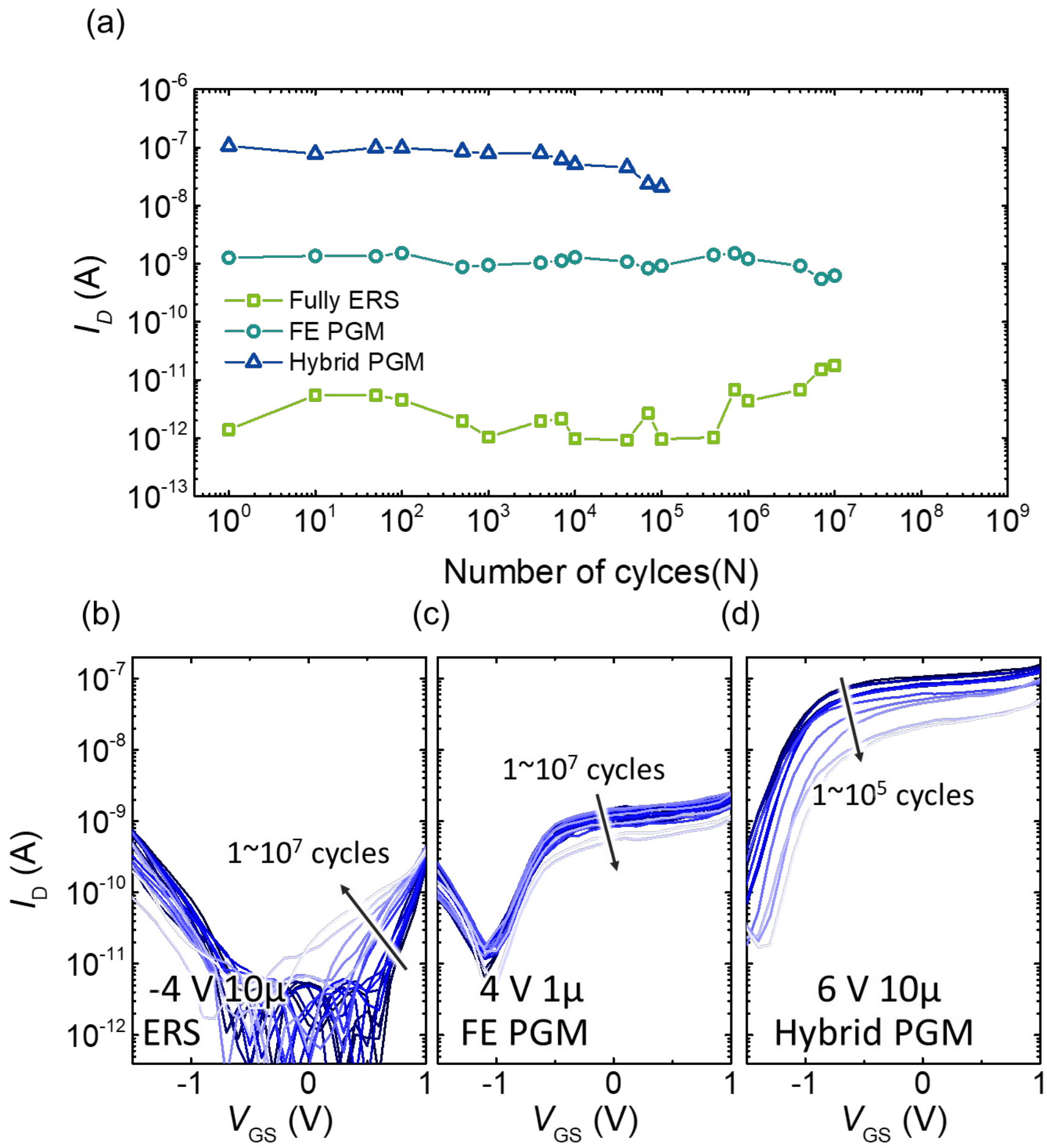

S20. Endurance characteristics of FITFET under different programming modes

(a) I_D evolution as a function of the number of switching cycles for fully ERS (-4 V 10 µs), FE PGM (4V 1 µs), and Hybrid PGM (6V 10 µs), demonstrating stable states without noticeable degradation. (b-d) Corresponding I_D-V_{GS} measured during endurance cycling for (b) fully ERS, (c) FE PGM, and (d) Hybrid PGM states.

Page 13 in the manuscript:

The robustness of the bias-controlled mode transition was further confirmed through repeated mode-toggling measurements and long-term endurance measurements in Supplementary Fig. S19 and S20.

Location of Change: Page 13 in the manuscript. Fig. S19 and 20 in the supplementary.

2. Are the ferroelectric, trapping, and ionic mechanisms truly separable and reproducible across devices? The manuscript implicitly treats the three mechanisms as bias-addressable and independent, yet lacks quantitative analysis of their mutual cross-coupling. Please provide bias-boundary statistics and discuss cross-coupling effects across multiple devices.

Response:

We thank the reviewer for the important comments regarding separability, device-to-device reproducibility, and potential cross-coupling the ferroelectric, trapping, and ionic mechanisms.

First, regarding the distinguishability between the ionic regime and the ferroelectric/trapping regimes across devices, Figs. S16 and S17 (retention and speed characteristics) indicate that each mechanism can be selectively activated depending on the applied voltage amplitude and pulse width. In particular, the ionic-motion-dominated regimes emerge only under high-voltage and long-pulse conditions, whereas under low-voltage conditions, the ferroelectric and trapping mechanisms predominantly govern the device behavior.

We emphasize, however, that the ionic regime presented in this study (hybrid PGM region) is not intended to define the primary operating mode of the FITFET. Rather, this regime is intentionally included to conceptually and electrically demonstrate that ionic species, such as oxygen vacancies, are physically present within the device and can be electrically activated. In this sense, the ionic regime serves as a supplementary operating region to illustrate the ferroelectric-ionic nature of the FITFET, rather than as its main functional mode.

The core operating modes of the FITFET are the plain mode (ferroelectric switching) and the secure mode (trap-mediated electrostatic screening), neither of which relies on ionic migration. Importantly, these two modes can be selectively addressed within the same device state simply by adjusting the read bias, and this bias-only mode control constitutes the central operating concept of the proposed device. This point has been insufficiently clarified in the original manuscript and has been explicitly addressed in the revised manuscript through additional clarification and discussion.

Nevertheless, as correctly pointed out by the reviewer, the original manuscript lacked sufficient quantitative analysis regarding bias boundaries and device-to-device reproducibility. In response, we have performed additional repeated sweep measurements focusing on the ferroelectric and trapping modes. The revised results demonstrate that these modes are well preserved over multiple consecutive sweeps within a single device and exhibit consistent behavior across multiple devices (revised Fig. S18). As shown in Fig. S18g and h, the MW collapse occurs reproducibly at V_{GS} in the range of approximately 1.18–1.20 V across multiple devices. In contrast, the onset of ionic contribution is identified from Fig. S18f, where a pronounced deviation associated with ionic transport emerges at higher V_{GS} of approximately 3.92 V. Based on these statistical results obtained from multiple devices, the trapping-dominated regime can be quantitatively defined in the V_{GS} range of 1.18–3.92 V, where bias conditions beyond this range correspond to the hybrid ferro-ionic regime.

These additional data and discussions have been incorporated into the revised manuscript and the supplement to strengthen the quantitative basis and reproducibility of the proposed bias-addressable operation.

Corresponding change in manuscript: Yes

Page 12 in the manuscript:

Both polarization- and ionic-driven switching behaviors exhibit high uniformity and reproducibility across the FITFET wafer, as confirmed by the statistical distributions in Supplementary Fig. 18. Specifically, the MW collapse associated with the trap-mediated secure mode occurs reproducibly at $V_{GS} \approx 1.18–1.21$ V across devices, while the onset of ionic contribution is observed beyond ~3.9 V, clearly defining the bias boundaries between the ferroelectric, trapping, and hybrid regimes.

Figure S18 in the supplementary:

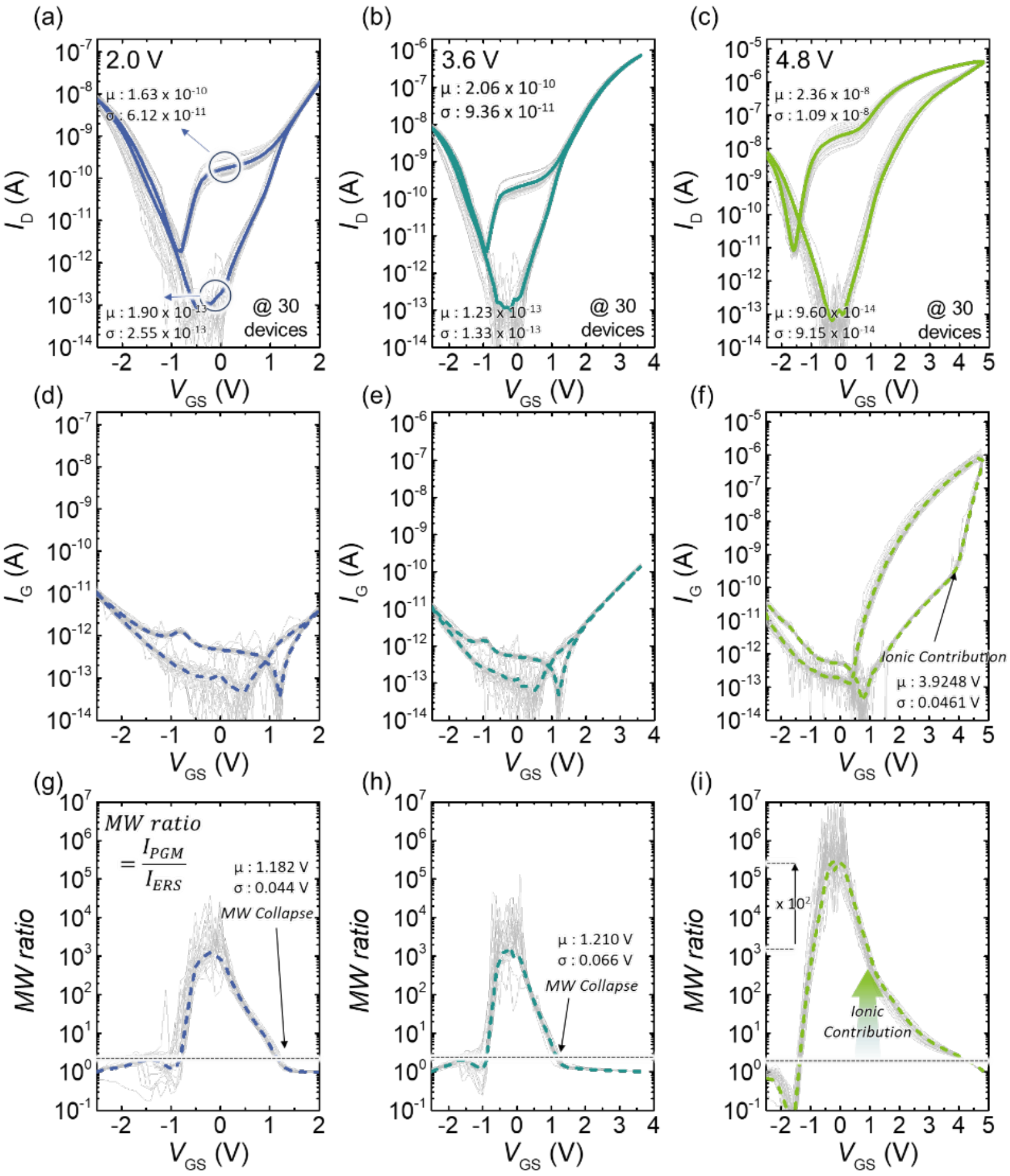

S18. Device-to-device uniformity of ferroelectric and hybrid ferro-ionic switching

Transfer characteristics ($I_{D,\,G}$-V_{GS}s) measured from 30 individual devices under (a, d) the FE-dominant, (b, e) trap-mediated and (c, f) hybrid ferro-ionic regimes, respectively. MW ratio measured at V_{GS} = 0 V under (g) FE-dominant, (h) trap-mediated, and (i) hybrid regime, showing consistent MW collapse at $V_{GS} \approx 1.18–1.21$ V and re-opening of the MW beyond ~3.9 V across devices.

Page 13 in the manuscript:

At higher pulse voltages (Fig. 4e), incremental pulse measurements were performed using 5.5 V to 7 V for potentiation and -3.5 V to -5 V for depression to probe the hybrid ferro-ionic switching operation beyond the purely ferroelectric switching observed in Fig. 4c. It should be noted that this hybrid ferro-ionic regime is not intended to enable information masking, but rather to explicitly probe the contribution of ionic species under high-bias conditions and to demonstrate an additional synaptic modulation mechanism accessible only in this elevated-bias regime. Under these conditions, the FITFET exhibits linear and symmetric current responses even within the secure mode region, which is attributed to excessive field-driven V_O migration across the HZO. In the plain mode region, the first pulse induces a steep current increase due to ferroelectric polarization switching, followed by a gradual and nearly linear evolution, indicating a transition toward the ferro-ionic hybrid state. These results confirm that when the applied bias is low, the FITFET can reliably store and conceal synaptic weights, whereas under sufficiently high bias, V_O migration becomes activated—serving as a supplementary high-bias operation mode rather than a concealment mechanism and providing direct physical evidence of the underlying ionic contribution to the encryption mechanism.

Location of Change: Page 12 and 13 in the manuscript. Fig. S18 in the supplementary.

3. Can the LFN-based defect depth interpretation be independently validated? Please correlate the LFN analysis with control structures or an additional experimental technique to support the proposed defect depth and mechanism assignment in this mixed ferroelectric–ionic system.

Response:

Thank you for this insightful comment. To independently validate the LFN-based defect-depth interpretation, we performed control-structure measurements on a reference FeFET with the same HZO ferroelectric stack, but with a thicker SiO_2 interlayer and without the TiO_2 insertion (i.e., SiO_2/HZO gate stack). As shown in the provided control data, the control FeFET exhibits conventional 1/*f* noise with exponent close to one that remains nearly unchanged with V_{GS}. Importantly, no bias-dependent humps, Lorentzian-like components, or regime-specific spectral features are observed. This is the expected behavior of a conventional carrier number fluctuation model with a broad distribution of time constants, where the dominant fluctuators are located near the SiO_2/Si channel interface and do not exhibit additional defect dynamics associated with ionic motion. Therefore, the control-structure comparison provides independent validation by establishing a baseline (pure 1/*f*, V_{GS}-independent) and showing that the extra noise features of the FITFETs originate from the engineered interface/ionic contributions. These additional data and discussions have been incorporated into the revised manuscript. Please refer to the revised manuscript to find the modifications.

Corresponding change in manuscript: Yes

Page 10 in the manuscript:

Note that this bias-dependent LFN behavior differs from conventional FeFETs, which typically exhibit a gate-bias-insensitive $1/f^{\gamma}$ spectrum with $\gamma = 1$, consistent with standard interface-trap-dominated fluctuations in a SiO_2–HZO stack (Supplementary Fig. 11).

Fig. S11 in the supplementary:

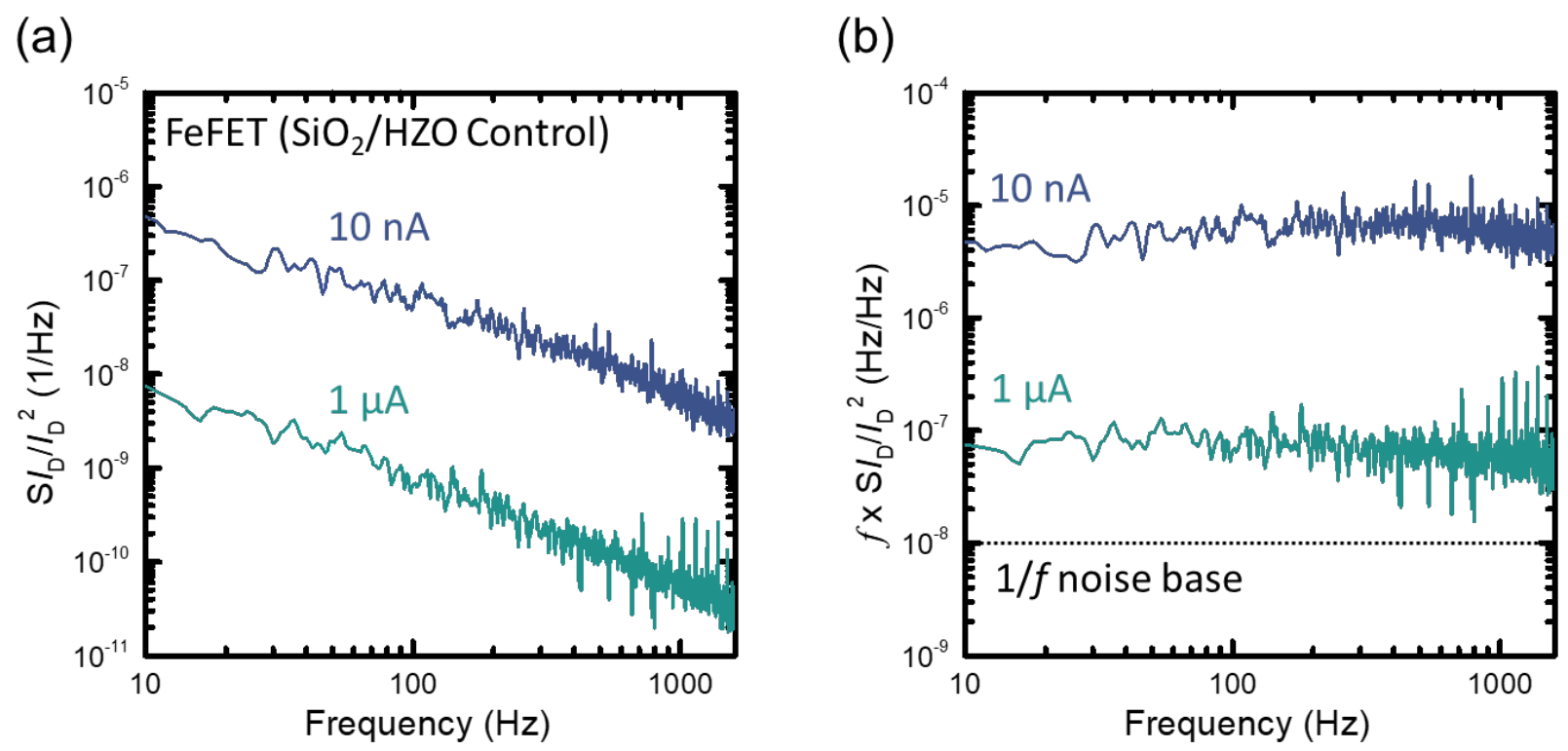

S11. LFN characteristics of control SiO_2/HZO FeFET.

(a) SI_D/I_D^2 and (b) $f \times SI_D/I_D^2$ versus frequency of the control SiO_2/HZO FeFET with a thicker SiO_2 interlayer and without the TiO_2 insertion

Location of Change: Page 10 in the manuscript. Fig. S11 in the supplementary.

4. Since the rate of ion movement is significantly slower than dipole flipping, the operating speed of the device becomes an issue. What is the minimum pulse width for the Hybrid PGM?

Response:

As correctly pointed out by the reviewer, ionic motion is slower than ferroelectric dipole flipping. As shown in Fig. 4a and Fig. S17, the ferroelectric PGM region and the hybrid PGM region can be clearly distinguished depending on the pulse amplitude and width. In the high-bias regime, hybrid PGM can be activated with relatively short pulse widths on the order of ~300 ns, whereas at lower bias conditions, much longer pulse widths in the range of several hundred microseconds are required due to the involvement of ionic motion.

However, it should be emphasized that the hybrid PGM regime is not the primary operating mode of the proposed FITFET. In the plain mode, which corresponds to the ferroelectric switching region, the device operates at high speed governed by dipole flipping, and no ionic motion is involved. Similarly, the secure mode does not rely on ionic migration but instead on trap-mediated electrostatic screening. Therefore, the overall operating speed of the FITFET is not fundamentally limited by ionic dynamics.

The hybrid PGM regime is intentionally accessed under high-bias conditions in this study to electrically demonstrate that ionic species, such as oxygen vacancies, are present and active within the FITFET stack. Building on this observation, the ionic-enhanced mode represents a special, high-bias operating condition that may be exploited in applications requiring a wider dynamic range through ionic-assisted synaptic functionalities; however, it is not required for the core plain/secure operation scheme. Therefore, this regime serves to illustrate the coexistence of ferroelectric-ionic phenomena in the device rather than to define its primary operating speed.

Corresponding change in manuscript: Yes

Page 13 in the manuscript:

It should be noted that this hybrid ferro-ionic regime is not intended to enable information masking, but rather to explicitly probe the contribution of ionic species under high-bias conditions and to demonstrate an additional synaptic modulation—potentially useful for applications requiring a wider dynamic range—that is accessible only in this elevated-bias regime. Under these conditions, the FITFET exhibits linear and symmetric current responses even within the secure mode region, which is attributed to excessive field-driven V_O migration across the HZO. In the plain mode region, the first pulse induces a steep current increase due to ferroelectric polarization switching, followed by a gradual and nearly linear evolution, indicating a transition toward the ferro-ionic hybrid state. These results confirm that when the applied bias is low, the FITFET can reliably store and conceal synaptic weights, whereas under sufficiently high bias, V_O migration becomes activated—serving as a supplementary high-bias operation mode rather than a concealment mechanism and providing direct physical evidence of the underlying ionic contribution to the encryption mechanism.

Location of Change: Page 13 the manuscript.

5. The author did not evaluate the consistency of the device array in the main text. How would the results be affected if only some devices in the array failed to successfully enter Secure Mode? Relevant explanations could be provided.

Response:

Thank you for your comment. We appreciate the reviewer's insightful observation that operational consistency is a critical factor for ensuring robust hardware-level security. To address this concern, we conducted additional system-level simulations to evaluate the impact of potential device-to-device (D2D) inconsistencies, specifically modeling scenarios where 20% of the FITFETs randomly failed to transition to Secure Mode. Notably, our simulation results demonstrate that even with a 20% failure rate, the reconstructed images via Model Inversion (MI) attacks remain highly distorted. Compared to the full Secure Mode, the Peak Signal-to-Noise Ratio (PSNR) increased by only 1.5%, and the Mean Squared Error (MSE) decreased by only 5.3%. These results indicate that a high level of pixel-level noise is still preserved, effectively concealing sensitive information from adversarial reconstruction. This resilience stems from the fact that input pixel information is spatially distributed across multiple synaptic weights within a layer. Therefore, the partial leakage of individual weights is insufficient for an attacker to recover the complete structural features of the input image. Consequently, our system provides reliable protection even in the presence of minor operational inconsistencies across the array. In accordance with the reviewer's comment, we have included these sensitivity analysis results and an expanded discussion on the statistical robustness of the FITFET array in the revised manuscript. We believe these additions further validate the practical reliability of our security mechanism. Thank you again for your careful review.

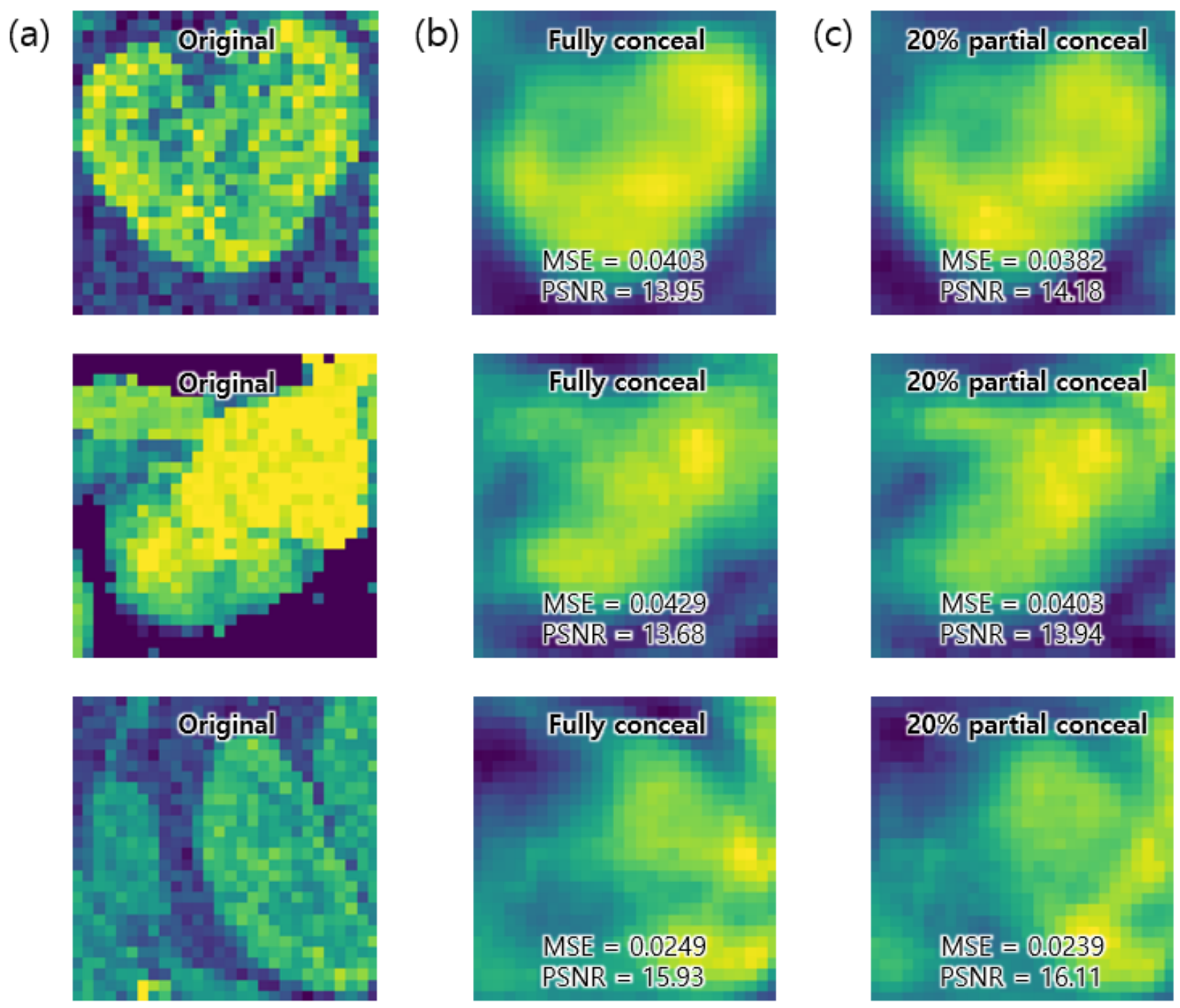

Figure R1. Robustness of the FITFET-based security mechanism against partial operational failures.

(a) Original input images used for simulation. (b) Reconstructed images under the ideal Full Secure Mode (100% transition). (c) Reconstructed images under a 20% random failure rate (partial Plain Mode operation).

Corresponding change in manuscript: Yes

Page 16 in the manuscript:

Furthermore, the FITFET-based security remains robust even in the presence of minor operational inconsistencies, such as a subset of devices failing to transition into Secure Mode. This resilience is fundamentally rooted in the fact that input information is spatially distributed across a multitude of synaptic weights within the network architecture. Consequently, even if individual weights are partially leaked due to device-level limitations, the incomplete information is insufficient for an adversary to reconstruct the full structural features of the original input. The inherent architectural robustness allows the system to provide reliable data protection while maintaining high operational stability, effectively mitigating the impact of physical device-level non-idealities.

Location of Change: Page 16 in the manuscript.

Reviewer #3

The authors report a ferroelectric–ionic–trapping field-effect transistor (FIT-FET) that integrates neuromorphic synaptic functionality with a hardware-native data concealment mechanism based on reversible memory window collapse. The concept of bias-programmable plain and secure operation modes is timely and original, and the manuscript is supported by a comprehensive set of experimental and system-level analyses, including atomic-scale characterization, in-situ low-frequency noise spectroscopy, array-level demonstrations, and security-oriented neural-network simulations. Overall, the work addresses an important challenge at the intersection of neuromorphic hardware and data security. I can recommend this work for publication in Nature Communications after the following points are clarified.

Response:

We sincerely thank reviewer for the thorough and constructive assessment of our manuscript. We greatly appreciate the reviewer's positive remarks regarding the originality of the bias-programmable plain and secure operation modes and the breadth of experimental and system-level validation. The recognition of the importance of this work at the interface of neuromorphic hardware and data security is particularly encouraging.

Below, we respond point by point to reviewer's comments and clarify the manuscript accordingly. Revisions have been made to enhance clarity and ensure that the key concepts and mechanisms are more clearly conveyed.

We, the authors, send the deepest appreciation for the encouragement and comments again.

1. The terms "plain mode" and "secure mode" are central to the manuscript and are used repeatedly throughout the text. However, their definitions are somewhat distributed across multiple sections. For improved clarity, the authors are encouraged to explicitly define these two modes at their first appearance in the main text, clearly stating the corresponding read-bias conditions and the resulting electrical behavior.

Response:

We thank the reviewer for the constructive comment regarding the definition of the plain and secure modes. In response to this suggestion, we have revised the Introduction to explicitly clarify the operating regimes of the plain and secure modes at their first appearance. Specifically, the plain mode is now clearly described as the low read-bias ferroelectric operating regime the enables reliable synaptic weight updates, while the secure mode is defined as the elevated read-bias regime in which interface charge trapping collapses the memory window to conceal the stored state. These revisions improve the clarity and consistency of the mode definitions throughout the manuscript.

Corresponding change in manuscript: Yes

Page 6 in the manuscript:

In the plain mode synapses, operated under a low read bias regime corresponding to the ferroelectric operating region, the minimal thickness of the IL ensures that the applied gate bias couples almost entirely into the HZO layer, yielding ultra-low-voltage synaptic weight updates (Fig. 1c). Furthermore, when the read bias is elevated, the device transitions into the secure mode (ii), in which charging traps at the IL to collapse the MW and thereby conceal the stored weight during readout without perturbing the non-volatile state (Fig. 1d).

Location of Change: Page 6 in the manuscript.

2. The role of the TiO_2 interlayer is discussed across several sections, including oxygen vacancy modulation, ionic switching, and interface engineering. While the explanations are thorough, the key takeaway may be diluted by repetition. For reader convenience, the authors may consider adding a single concise sentence summarizing the primary function of the TiO_2 layer in the FIT-FET, for example in the device structure section or concluding paragraph of the mechanism discussion.

Response:

We thank the reviewer for the helpful suggestion regarding the role of the TiO_2 interlayer. In response, we have added a concise summary sentence in the Conclusion that explicitly highlights the primary function of the TiO_2 interlayer in the FITFET. We believe that this revision helps clarify the central message of the manuscript and improves its overall readability.

Corresponding change in manuscript: Yes

Page 22 in the manuscript:

By integrating an ultrathin SiO_2/TiO_2 interfacial stack with an HZO ferroelectric layer, the FITFET exploits a coupled ferro-ionic–trapping complex that is selectively addressable by gate bias. In this architecture, the TiO_2 interlayer functions as a multifunctional interface that simultaneously maintains effective ferroelectric-to-channel coupling at low bias and enables bias-programmable charge trapping for non-destructive memory concealment.

Location of Change: Page 22 in the manuscript.

3. It should be noted that the p1/p2 intensity ratio reflects a relative trend in VO concentration rather than an absolute quantitative concentration. Explicitly acknowledging this limitation in the manuscript or the supplementary information would help readers interpret the EELS results more appropriately and avoid potential over-quantification.

Response:

We thank the reviewer for pointing out this important limitation regarding the interpretation of the p_1/p_2 intensity ratio in the EELS analysis. We fully agree that the p_1/p_2 ratio reflects relative trends in V_O concentration rather than an absolute quantitative measure. In response, we have explicitly acknowledged this limitation in both the main text and supplement to avoid potential over-quantification and to guide appropriate interpretation of the EELS results. We believe that these clarifications improve the rigor and transparency of the EELS analysis and help readers more accurately interpret the defect-related trends discussed in this work.

Corresponding change in manuscript: Yes

Page 7 in the manuscript:

EELS analysis revealed a clear reduction in the p_1/p_2 intensity ratio near the HZO/IL interface, indicating a higher relative concentration of V_Os in the transistors with TiO_2 layer compared to the FeFET without TiO_2.

Note S2 in the Supplement:

It should be noted that the p_1/p_2 intensity ratio reflects relative trends in V_O concentration rather than an absolute quantitative measure and is therefore used here as a comparative indicator of spatial variation.

Location of Change: Page 7 in the manuscript. Note S2 in the Supplementary.

4. The authors are encouraged to expand the XPS-related discussion, particularly by more clearly describing the peak deconvolution procedure of the O 1s spectra, the physical meaning of each fitted component, and how the extracted oxide-deficient fraction is correlated with VO concentration.

Response:

We thank the reviewer for the constructive comment regarding the XPS analysis. We have expanded the XPS-related discussion in supplement to improve clarity and rigor. Specifically, we now describe the O 1s peak deconvolution process, explain the meaning of each fitted component, and clarify how the extracted oxide-deficient fraction is interpreted in relation to V_O. These revisions are intended to help readers more appropriately interpret the XPS results.

Corresponding change in manuscript: Yes

Note S3 in the Supplement:

To evaluate the chemical states and depth-dependent oxygen stoichiometry in the fabricated transistors, X-ray photoelectron spectroscopy (XPS) was performed using a Thermo Fisher Scientific NEXSA system. All spectra were calibrated to the C 1s reference peak at 284.8 eV. The O 1s core-level spectra were deconvoluted into two Gaussian components following established fitting procedures reported for hafnia-based oxides[10,11]. The lower binding energy peak represents oxygen in Hf-O or Zr-O coordination, whereas the higher-binding-energy component corresponds to oxygen-deficient or non-lattice oxygen species, which are commonly associated with V_Os.

Location of Change: Note S3 in the Supplementary.

5. In general, most figures clearly specify the applied voltage conditions, such as amplitude and pulse width, which helps readers understand the measurement protocols. However, in some figures, this information is missing or only partially described, making it difficult for readers to immediately grasp the experimental conditions. This issue is particularly noticeable in the array-level measurement data, where multiple bias conditions are used for program, read, inhibit, and erase operations.

Response:

We thank the reviewer for pointing out this issue. Following the reviewer's suggestion, we carefully reviewed the figures and identified instances where voltage conditions and pulse paraments were missing or only partially described. These details have now been added or clarified, particularly for the array-level measurements involving program, read, inhibit, and erase operations. We believe that these revisions improve the clarity of the experimental conditions and help readers more easily understand the measurement protocols. We thank the reviewer again for this helpful comment.

Corresponding change in manuscript: Yes

Fig. S21 in the supplementary:

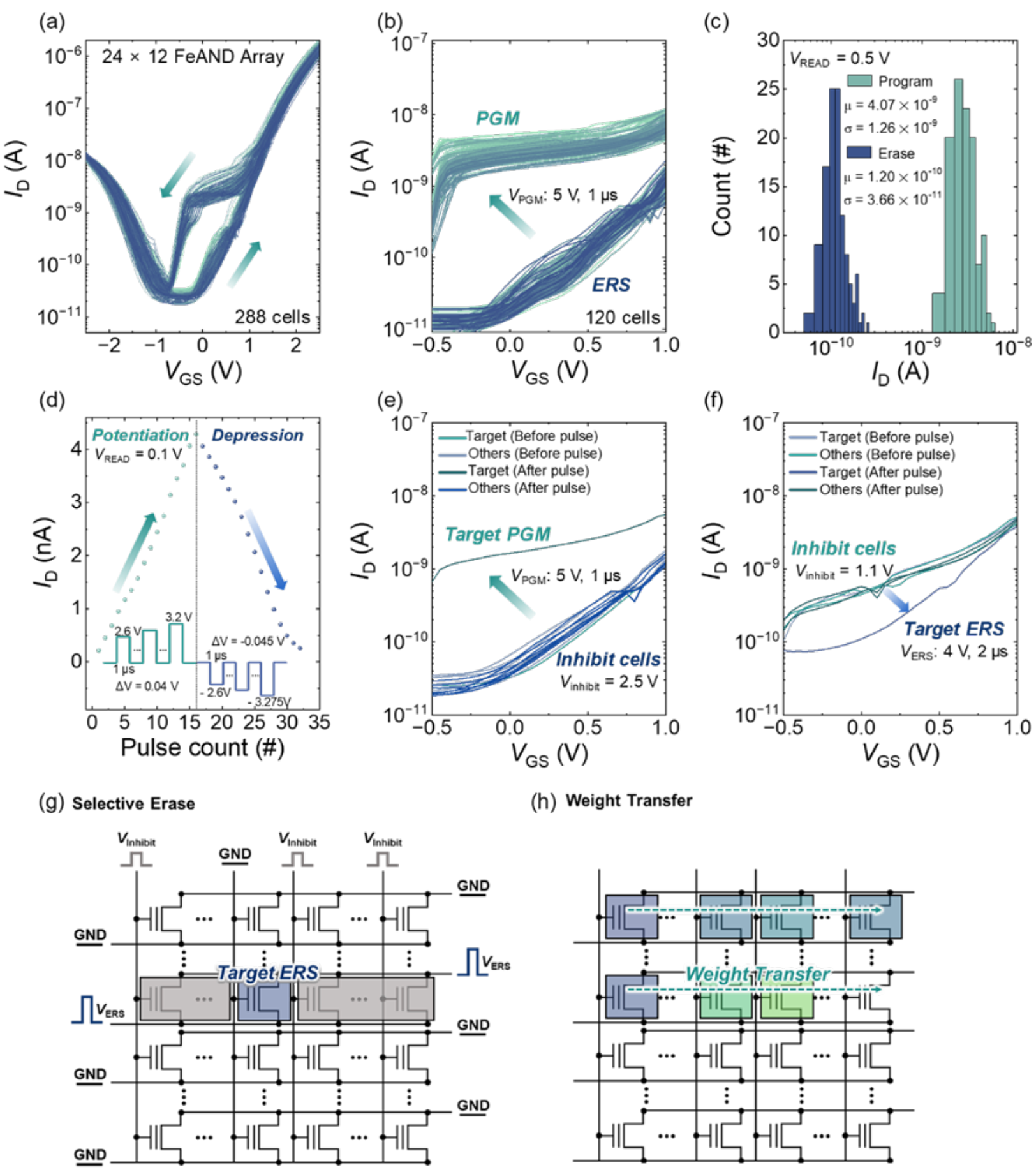

Fig. S22 in the supplementary:

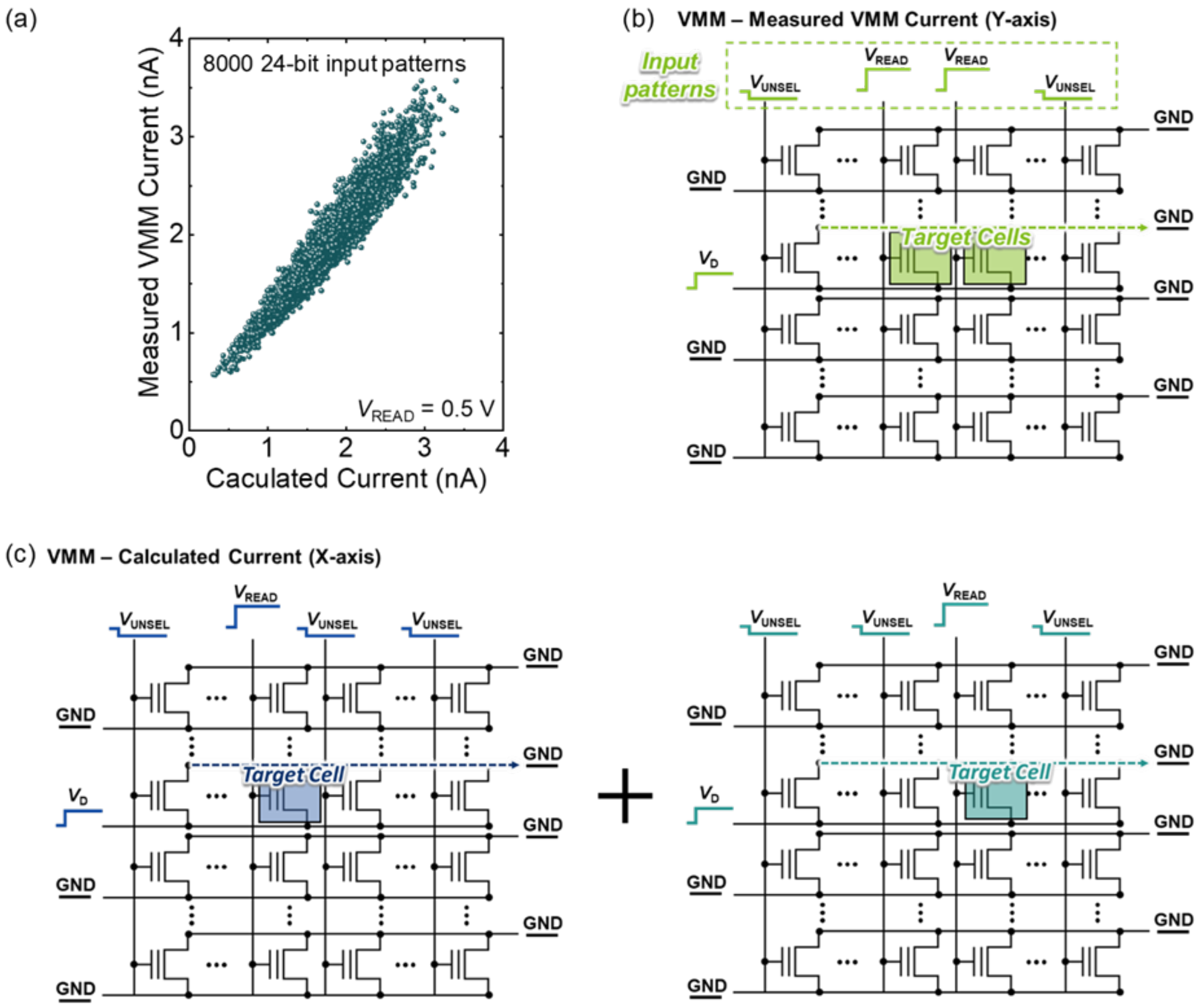

Location of Change: Fig. S21 and S22 in the supplementary.

Revision and Correction (NCOMMS-25-98264B)

Dear Reviewers,

We, the authors, appreciate the time and effort that the reviewers dedicated to providing feedback on our manuscript and are grateful for the insightful comments on our work. We read the comments and questions carefully and prepared a response sheet for them. Please refer to the responses below. The responses that need to be addressed in the revised manuscript are highlighted so that the reviewers can trace them with more ease. Also, the overall sentences have been refined with more concise expressions and better English grammar. We heartily thank all the comments and coordination again.

Reviewer #1

1. For question (1), the estimate of 3% accuracy drop is based on simulation of devices in good condition. If the device work continuously in the safe mode with a lot of interface trapping effects, is it possible to cause further degradation of the accuracy due to Vth shift or increased low-frequency noise? Please provide some further discussions in this issue.

Response:

Thank you for the insightful comment. To address the concern regarding potential accuracy degradation under prolonged safe-mode operation, we experimentally investigated both V_{th} and LFN behavior under repeated secure-mode stress. First, to examine the V_{th} shift induced by continuous secure-mode operation, we performed additional measurements by repeatedly applying 1.25 V, 50 ns pulses for secure-mode operation, in addition to the results shown in Figure 4f. As shown in Figure R1, a slight current increase is observed after 10^6 cycles; however, the magnitude is on the order of ~1 pA. Considering that the read-current window spans from the fully erased state (~1 pA) to the fully programmed ferroelectric state (~1 nA) (Figure 4a), this variation is negligible. Furthermore, as observed in Figure 4f, a similar minor increase appears not only under repetitive pulses but also during long-term measurement. However, this change is too small to be interpreted as unintended programming, and the current rapidly recovers to its initial level. These results indicate that although minor trapping effects may occur under prolonged secure-mode operation, they do not lead to meaningful shift or degradation in device operation.

In addition, regarding the reviewer's comment on LFN, no evidence of noise increment was observed under repeated secure-mode read operation (Figure R2). An increase in LFN typically requires a significant increase in active oxide/interface defect density. In such cases, accompanying signatures such as pronounced V_{th} shift, noticeable I_D drift, or deterioration of the read-current window are generally expected. However, none of these signatures were observed in our repeated secure-mode stress measurements (Fig. R1). This implies that the physical mechanisms responsible for LFN enhancement, such as substantial generation or activation of additional oxide/interface defects, are not triggered under the secure-mode operation conditions used in this work.

Therefore, both experimental V_{th} stability and LFN analysis consistently indicate that continuous secure-mode operation does not introduce additional degradation mechanisms and thus is unlikely to cause further accuracy loss beyond the estimated level.

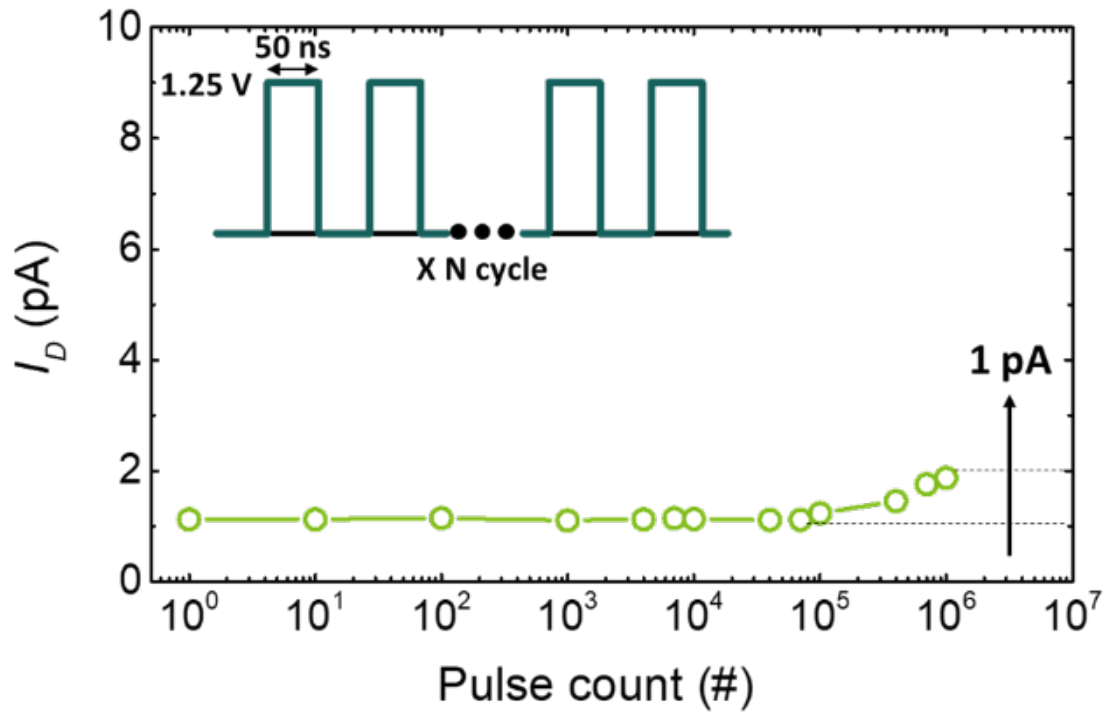

Figure R1. I_D evolution as a function of pulse count under repeated application of 1.25 V, 50 ns pulses.

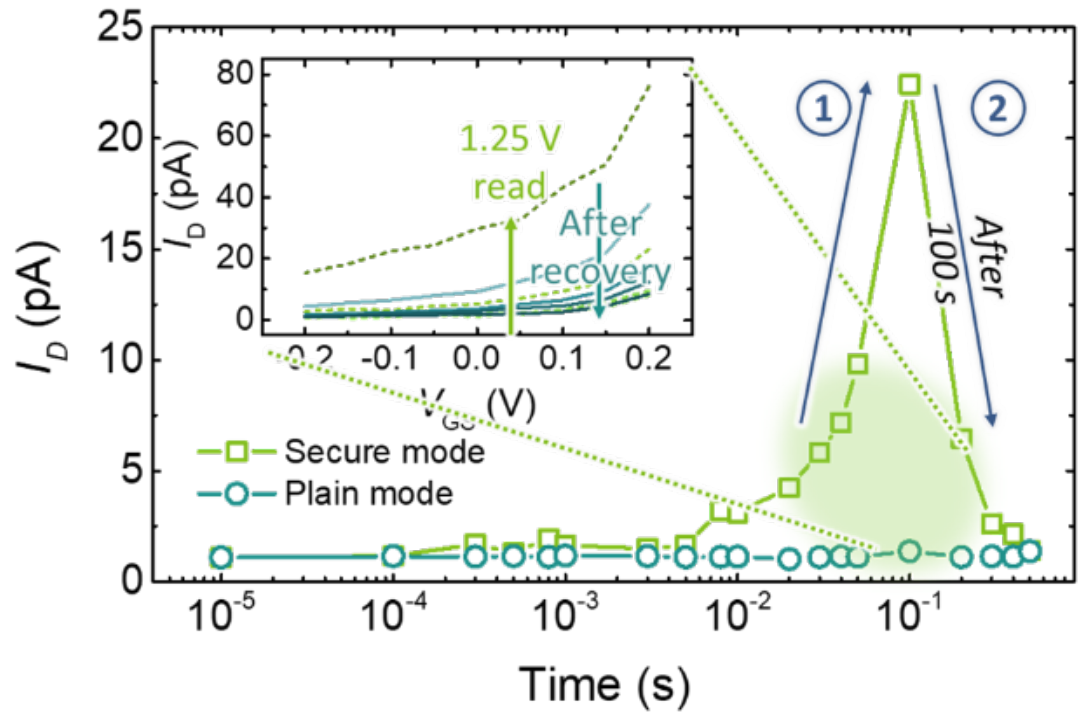

Figure 4f. Read-state stability test under plain and secure modes. Secure mode read induces a temporary polarization disturbance followed by full recovery after removing the read bias.

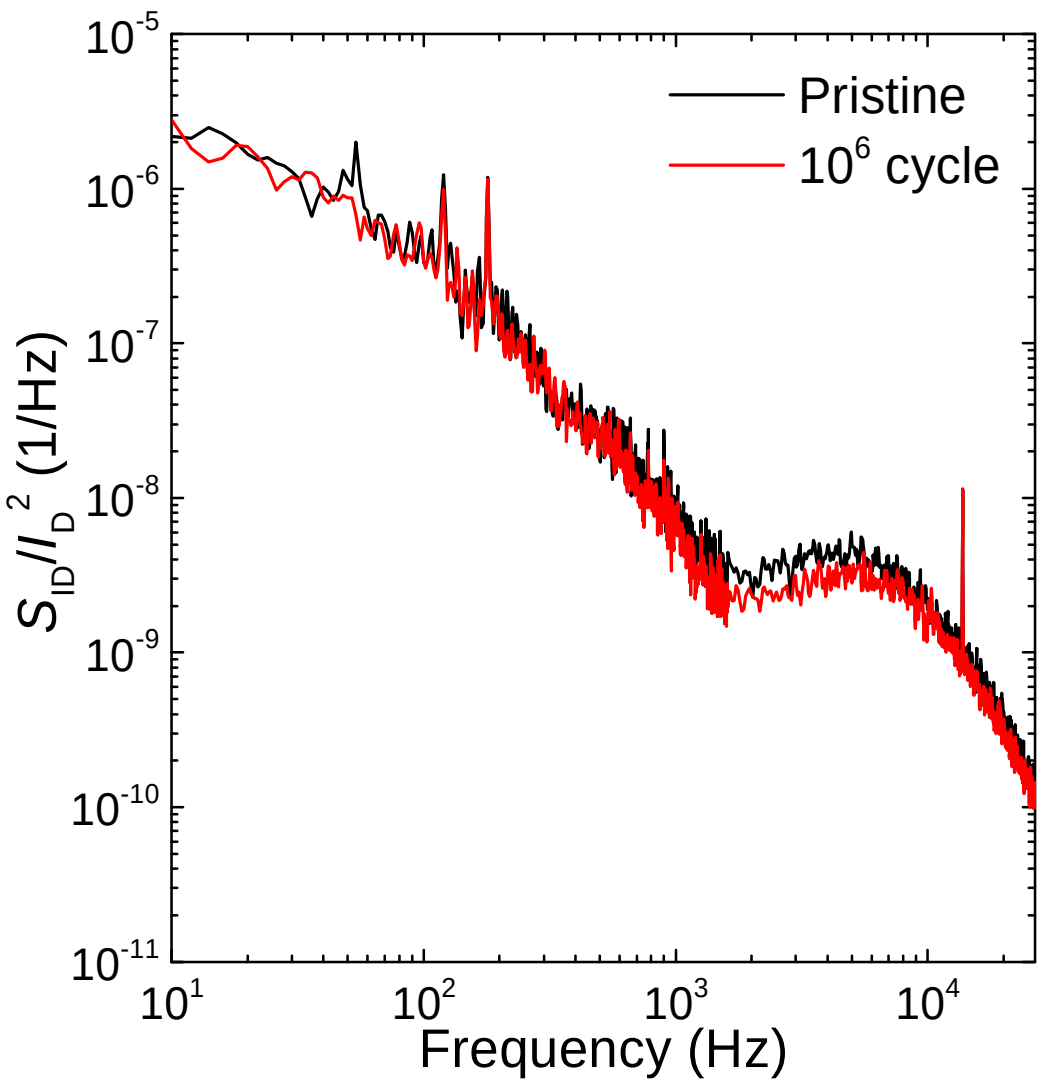

Figure R1. S_{ID}/I_D^2 versus frequency of the FITFET before and after 10^6 cycles of 1.25 V, 50 ns pulses.

Corresponding change in manuscript: Yes

Supplementary Figure 21:

S21. Repeated secure mode stress test of the FITFET

Evolution of ID as a function of pulse count under repeated application of secure mode pulses (1.25 V, 50 ns), showing negligible current variation (~1 pA) and stable operation up to 106 cycles without cumulative degradation or unintended programming.

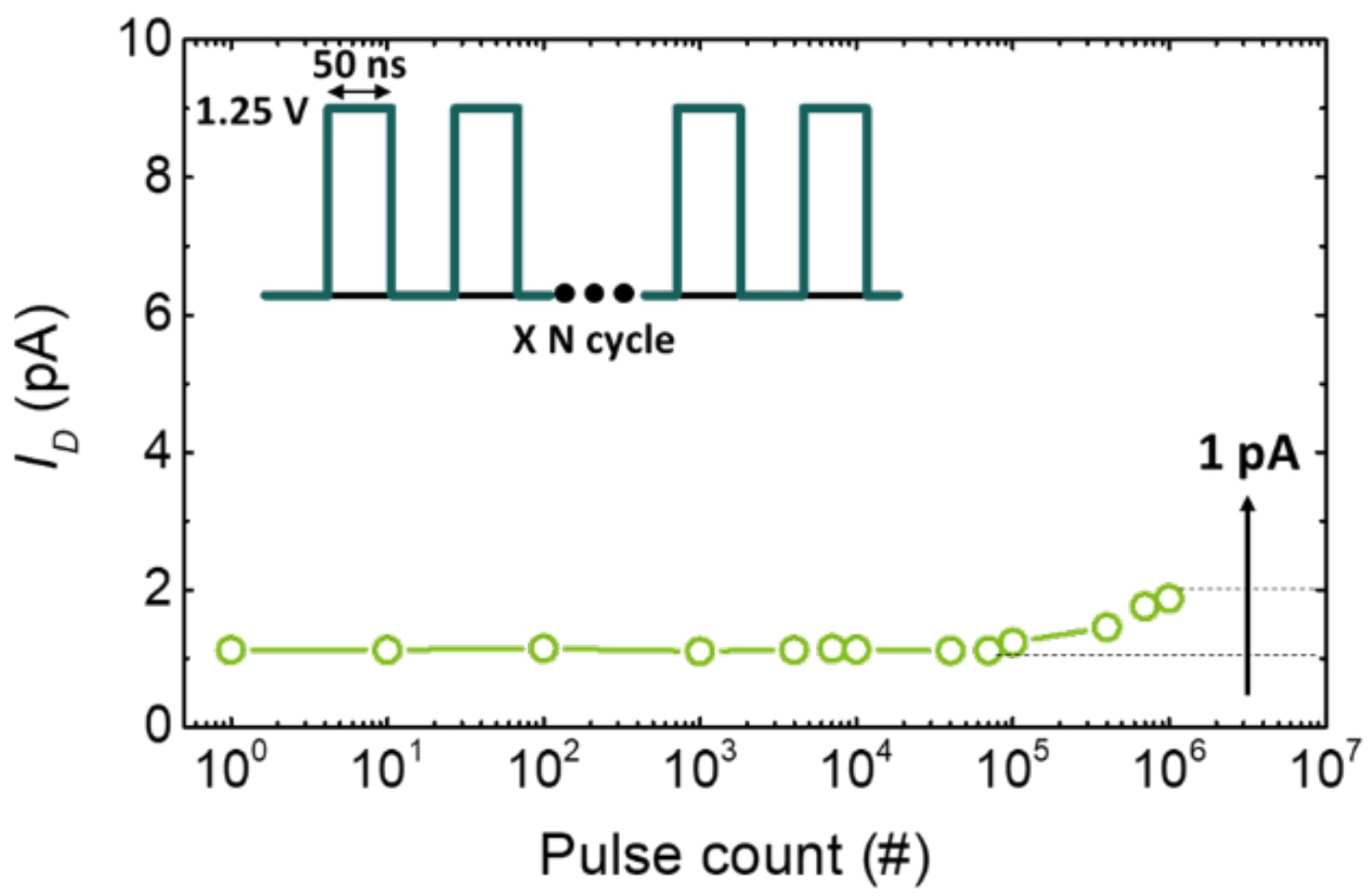

Page 14 in the manuscript:

This stability is further supported by the negligible current evolution under repeated secure-mode pulse stress (Supplementary Fig. 21).

Location of Change: Page 14 in the manuscript. Supplementary Figure 21 in the supplement.

2. For question (8), the authors accepted part of my suggestion by adding more benchmark figures. While the safety of different PUF designs are hard to evaluate, I still recommend the authors to evaluate the energy or power consumption the PUF devices, especially in the safe mode, and compare it with literatures.

Response:

Thank you for this valuable suggestion. We agree that an energy-oriented comparison can further strengthen the practical significance of the benchmark table. At the same time, however, we would like to emphasize that a strict one-to-one comparison of absolute energy across the prior works is not fully fair, because the compared devices address substantially different system roles and operation schemes. In particular, most prior concealment-oriented PUF studies are dedicated security primitives, whereas the proposed FITFET intrinsically combines synaptic functionality and hardware-level concealment within the same transistor. Accordingly, many previous works require an explicit conceal/recover sequence, such as SET/RESET, overwrite/re-generation, or a separate security-only operation, on top of storage or computation. In contrast, the FITFET enters the secure state simply by shifting the read bias of the same synaptic transistor, without requiring a dedicated PUF block or an additional conceal/recover programming step. To address the reviewer's comment in a more transparent and academically rigorous manner, we revised the benchmarking methodology and supplemented both Table S2 and the Supplementary Information accordingly. Specifically, instead of forcing a single universal energy number, we separated the comparison into two descriptors: (i) synaptic/compute functionality and (ii) additional security-function energy overhead. Under this framework, PUF-only devices are distinguished from monolithically integrated synapse/security devices, and the reported or reconstructable energy terms are presented according to the actual operating principle of each work. For papers that directly report energy or provide sufficient operating conditions, we summarize the corresponding energy expressions or reported values. For papers in which essential parameters such as pulse width, transient current, or active optical area were not quantitatively disclosed, we intentionally avoided over-interpreting the data and instead described the governing energy expressions and benchmarking rationale in **Supplementary Note 9**.

Corresponding change in manuscript: Yes

Supplementary Note 9 :

Supplementary Note 9. Methodology for energy benchmarking of concealment-oriented security devices in Table S2

The prior works compared in Supplementary Table S2 address heterogeneous device roles, including security-only PUFs, unified PUF/TRNG chips, in-memory encryption engines, and optoelectronic synapse/PUF hybrids. Therefore, a single universal energy number is not physically meaningful. In particular, the proposed FITFET differs fundamentally from most prior works in that synaptic functionality and hardware-level concealment are co-implemented in the same transistor, whereas many previous devices realize security through a dedicated PUF primitive or through an explicit conceal/recover programming sequence, which is separated from synaptic devices. Therefore, the benchmark was organized using two complementary descriptors: (1) synaptic/compute

functionality, and (2) additional security-function energy overhead. For PUF-only devices, the synaptic term is not applicable. For each literature entry, we either adopted the energy value directly reported in the primary paper or conservatively reconstructed the governing energy expression from explicitly disclosed operating conditions. When essential parameters such as pulse width, transient current, or active illuminated area were not quantitatively mentioned, we intentionally avoided back-calculating an absolute energy number and reported only the corresponding governing expression.

In general, the operation energy is defined by

$$E = \int_0^{t_{\mathrm{op}}} V(t)I(t)\, dt.$$

For a resistive read operation under quasi-static bias, this reduces to

$$E_{\mathrm{read}} \approx VIt = \frac{V^2}{R}t = GV^2t,$$

where R and G denote effective resistance and conductance, respectively. For a multi-element circuit, the total energy can be written as

$$E_{\mathrm{tot}} = \sum_k \int V_k(t)I_k(t)\, dt.$$

If the original paper reports only a power trace, the energetically faithful quantity is

$$E = \int P(t)\, dt.$$

For optoelectronic devices, the total operation energy is more appropriately separated into electrical and optical contributions

$$E_{\mathrm{tot}} = E_{\mathrm{elec}} + E_{\mathrm{opt}},$$

with

$$E_{\mathrm{elec}} = \int V(t)I(t)\, dt, and\ E_{\mathrm{opt}} = \mathcal{I}_{\mathrm{opt}}At,$$

where $\mathcal{I}_{\mathrm{opt}}$ is the optical intensity and A is the illuminated area. Unless otherwise stated in the original paper, device-level estimates exclude peripheral circuits, line charging, and controller overhead.

This work: FITFET

For the proposed FITFET, the security function is realized by read-bias selection in the same synaptic transistor, rather than by a separate PUF block or by an explicit conceal/recover write sequence. Accordingly, the incremental security-step energy overhead at the device-architecture level is effectively negligible, because no additional SET/RESET-style concealment step is required to enter the secure state. Nevertheless, to provide a device-level number, we estimated the selected-cell drain-side secure-read energy using the experimentally used array-level read condition and the conductance range observed during the conceal–reveal operation. The corresponding expression is

$$E = \int_0^{t_r} V_{DS}\, I_D(t)\, dt \approx G_{\mathrm{sec}}V_{DS}^2t_r.$$

Using V_{DS} = 0.1 V and t_r = 1 μs, and G = 0.45 μS, the selected-cell secure-read energy is estimated to be 4.5 fJ. This estimate is intentionally restricted to the selected-cell, drain-side read path and does not include array-size-dependent peripheral contributions such as WL/BL line charging, decoder overhead, or the bias applied to unselected cells. This separation is important because the key architectural advantage of the FITFET lies not in a dedicated security-writing event, but in the fact that the secure state is accessed without a separate security-only operation.

Gao et al. (ref. 51)

Gao et al. reported a concealable memristive PUF in which readout mode and secure mode are achieved by a one-pulse RESET and SET operation, respectively. The paper explicitly presents the power track during the recovery and concealing of a 128-bit ID and the distribution of power consumption for one PUF bit, while also stating that only the power consumed in the memristor array is considered. Therefore, the physically faithful energy expressions for this work are

$$E_{\text{Gao,128-}bit} = \int P_{\text{array}}(t)\, dt$$

and

$$E_{\text{Gao,}bit} = \frac{1}{128}\int P_{\text{array}}(t)\, dt.$$

Because the main text does not tabulate the full numerical time-axis data for the power traces, we did not back-calculate a single absolute energy number for Table S2. Instead, we retained the operation principle that the security function requires an explicit SET/RESET-based conceal/recover step.

Li et al. (ref. 52)

Li et al. demonstrated a unified 28-nm 2T2R memristor-based PUF/TRNG chip with a concealable function. The paper explicitly reports that the PUF/TRNG read operation uses V_{read} = 0.2 V and that valid outputs are obtained within 100 ns, while chip-level PUF throughput reaches 67.6 Mbps. It also reports that concealment and recovery are implemented by SET/RESET operations over the entire array, with V_{set} = 2.7 V and V_{reset} = 3.0 V both using 4 μs pulses. On this basis, the pair-read energy of one 2T2R entropy cell can be written as

$$E_{\text{Li,read}} = \int_0^{t_r} V_{\text{READ}}\, I_{\text{pair}}(t)\, dt \approx \frac{V_{\text{READ}}^2}{R_{\text{top}}+R_{\text{bot}}} t_r,$$

where R_{top} and R_{bot} are the instantaneous read-state resistances of the two memristors that define the PUF bit. The explicit security-step overhead is

$$E_{\text{Li,conceal}} = \int_0^{4\mu s} V_{\text{SET}}\, I_{\text{SET}}(t)\, dt,$$

$$E_{\text{Li,recover}} = \int_0^{4\mu s} V_{\text{RESET}}\, I_{\text{RESET}}(t)\, dt.$$

Because the dynamic switching current traces and machine-readable read-state resistance values are not numerically tabulated in the main text, we did not force a single absolute number for the conceal/recover energy.

Nevertheless, this paper clearly differs from the FITFET in that concealment incurs a nonzero additional security-operation overhead, since the entire array must be explicitly SET/RESET to conceal and recover the PUF state.

Woo et al. (ref. 53)

Woo et al. reported task-level energies directly for a memristor-based platform combining unclonable key generation, logic, and encryption/decryption. The paper already tabulates total energy and latency for each task. The reported values are 99.9 pJ for encryption/decryption, and 134.3 pJ for SUM. Since the work does not operate as an analog synapse with bias-programmable secure readout, it was classified separately from the FITFET in the monolithic synapse/security descriptor

Kim et al. (ref. 54)

Kim et al. presented a chiroptical synaptic perovskite memristor that functions as a reconfigurable PUF, thus representing the closest prior example of a monolithic synapse/security concept. However, this platform is fundamentally optoelectronic, and its reconfiguration relies on a simultaneous electrical and polarized-light stimulus. The paper reports that the 10 × 10 CSM array was reset by a 2.0 V electrical sweep and programmed to HRS using a -1.0 V pulse for 0.5 s under a selected polarized-light condition at a constant optical intensity of 100 μW/cm^2. Therefore, the appropriate total reconfiguration energy is 50 μJ/cm^2. However, because the active illuminated area and the full electrical transient during the 0.5-s programming event are not numerically tabulated, we did not extract a per-device or per-bit total energy.

Cho et al. (ref. 55)

Cho et al. introduced a vertical self-rectifying memristor platform in which PUF key generation and XOR-based in-memory encryption are realized in the same V-RRAM structure. Importantly, this study primarily benchmarked spatiotemporal cost, rather than providing a directly tabulated electrical energy value. The paper states that key generation is performed through a voltage-divider stage using a series resistance. The voltage applied across the (i,j)-th device may therefore be written in the standard voltage-divider form as

$$V_{A,i,j} = V_{G,i} \frac{R_{i,j}}{R_S+R_{i,j}},$$

where $V_{G,i}$ is the generation voltage applied to the i-th WL and $R_{i,j}$ is the resistance of the selected cell. During XOR operation, the paper reports a NIMP voltage V_{NIMP} = 5.0 V, a read voltage of 6 V, and a compliance current of I_{CC} = 50 nA. For the anti-serial NIMP pair, the voltage across the reverse-biased memristor follows the voltage-divider relation

$$V_{M1} = V_{\mathrm{NIMP}} \frac{R_{M1}}{R_{M1}+R_{M2}}.$$

Accordingly, the NIMP energy may be bounded by

$$E_{\mathrm{NIMP}} \leq V_{\mathrm{NIMP}} I_{CC} t_p,$$

where t_p is the NIMP pulse width. For a single XOR pair, the corresponding operation-level expression can be written as

$$E_{\mathrm{XOR,pair}} \approx 2E_{\mathrm{NIMP}} + E_{\mathrm{OR}}.$$

However, because the main text does not numerically tabulate the NIMP pulse width or the OR-operation current/time product, we did not extract a single absolute electrical energy. Moreover, the encryption function in this platform is inherently realized through a multi-step XOR logic sequence rather than a bias-selectable secure-read mechanism. Therefore, a fair energy interpretation should include not only the device-level switching event itself, but also the associated logic-flow overhead required for XOR evaluation, including intermediate logic operations and their supporting peripheral control. In this sense, the effective system-level overhead for security operation is intrinsically larger than that of a bias-programmable secure-read device such as the FITFET, even though a directly comparable absolute energy value is not explicitly provided in the original paper.

Park et al. (ref. 56)

Park et al. reported a concealable PUF using commercial V-NAND flash memory, in which the entropy source is generated by weak GIDL erase. The paper explicitly shows that a weak GIDL condition around V_{GIDL} = 3.0 V produces a suitable bimodal threshold-voltage distribution for PUF formation and also emphasizes that GIDL erase is a native operation of V-NAND flash memory, such that no additional functionality or circuitry is required. The relevant operation energies can therefore be written generically as

$$E_{\mathrm{Park,GIDL}} = \int V_{\mathrm{GIDL}} I_{\mathrm{BL}}(t)\, dt,$$
$$E_{\mathrm{Park,conceal}} = \int V_{\mathrm{prog}}(t) I_{\mathrm{prog}}(t)\, dt,$$
$$E_{\mathrm{Park,read}} = \int V_{\mathrm{read}} I_{\mathrm{BL}}(t)\, dt.$$

Here, the concealment process corresponds to overwriting the PUF with user data, while the reveal process corresponds to re-establishing the PUF data through GIDL erase. Since the main text does not provide full numerical current transients or pulse widths for these operations, we did not back-calculate a single absolute energy value. The key distinction from the FITFET is that, although no extra circuitry is required, concealment/recovery still relies on an overwrite-and-reveal sequence, rather than on a bias-only secure read condition in the same synaptic state.

Page 21 in the manuscript :

As summarized in Table S2, the unique combination of voltage-tunable synaptic and trap-mediated data concealment of FITFET distinguishes it from conventional filament-based memories, offering CMOS-compatible platform where computation and protection coexist within the single device[51–56] (Supplementary Note 9).

Location of Change: Page 21 in the manuscript. Supplementary Note 9 in the supplement.
